# The prevention of heterotopic ossification around the knee: a scoping review

**DOI:** 10.1186/s12891-026-10318-w

**Published:** 2026-08-01

**Authors:** Maximilian Römer, Georg Wurschi, Matthias Mäurer, Klaus Pietschmann

**Affiliations:** Department of Radiotherapy and Radiation Oncology, Jena University Hospital, Am Klinikum 1, Jena, 07747 Germany

**Keywords:** Heterotopic ossification, Knee, Prophylaxis, Prevention, Scoping review, Continuous passive motion, Non-steroidal anti-inflammatory drugs, Bisphosphonates, Radiotherapy, Surgery

## Abstract

**Background:**

Evidence on prophylactic interventions to prevent heterotopic ossification (HO) around the knee is scarce. We conducted a scoping review to map reported indications, interventions, and outcome reporting.

**Methods:**

We followed a prospectively developed protocol and searched four major databases (MEDLINE via PubMed, Embase, Cochrane Library, and Web of Science), complemented by searches of trial registries and grey literature using a high-sensitivity strategy. Data extraction was performed in duplicate and summarized descriptively by modality.

**Results:**

The search yielded 2,958 records. We included 78 unique publications, comprising 18 expert-opinion sources and 62 clinical publications reporting data on 3,321 patients, of whom 1,579 received the evaluated interventions, with two publications contributing to both syntheses. Modalities comprised continuous passive motion (CPM) in 8, pharmacological prophylaxis in 21, radiotherapy (RT) in 18, surgical techniques in 3, and combination therapy in 14 studies. Most identified studies were case reports or case series. Pharmacological data were largely driven by one large ASA cohort, whereas RT and combination-therapy studies were small and mainly addressed recurrence prophylaxis. One late fatal, potentially RT-induced sarcoma was reported. Because of substantial clinical and methodological heterogeneity, crude reported event proportions should not be compared directly across modalities. Outcome reporting was inconsistent, with limited use of standardized patient-reported outcomes and incomplete adverse-event reporting.

**Conclusions:**

Knee HO prophylaxis evidence is dominated by low-level, heterogeneous studies with substantial reporting gaps. Comparative knee-specific studies with standardized outcome definitions, functional outcomes, and systematic safety reporting are needed.

**Trial registration:**

This review was prospectively registered on the Open Science Framework (OSF registration ID 5328k).

**Graphical Abstract:**

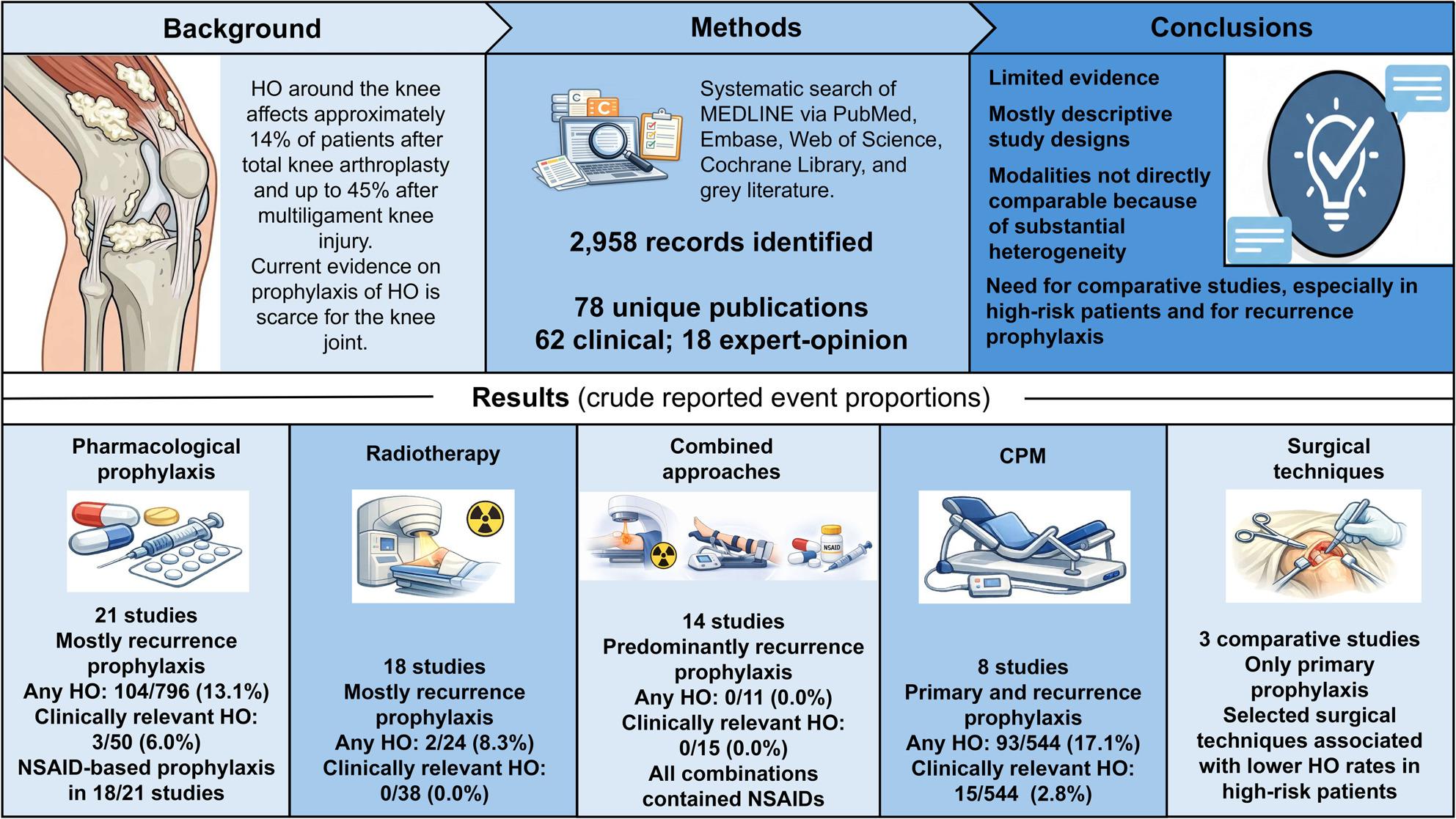

**Supplementary Information:**

The online version contains supplementary material available at 10.1186/s12891-026-10318-w.

## Background

Heterotopic ossification (HO) refers to the ectopic formation of extraskeletal bone in soft tissues like muscle, skin, and ligaments as a result of a complex and multifactorial pathologic process that is still incompletely understood. Depending on its size and location, HO may be asymptomatic, only diagnosed radiographically, or result in severe symptoms and functional limitations. The early symptoms of HO include local swelling and pain followed by progressive and often persistent reduction in range of motion (ROM) due to mechanical obstruction [1]. Traumatic HO is of particular interest for orthopedic surgeons, as HO may develop after trauma or orthopedic surgery, including total joint arthroplasty. There have been several randomized controlled trials (RCTs) and meta-analyses investigating prophylactic interventions to prevent HO around the hip [2]. HO around the knee joint appears to be less common than around the hip joint, with a meta-analysis reporting a relatively low HO rate of 14% after total knee arthroplasty (TKA) [3]. However, more than 700,000 knee arthroplasty procedures were performed during inpatient hospital stays in the USA in 2018, indicating that HO around the knee may still affect a substantial number of individuals [4]. Additionally, for other conditions, the incidence of HO around the knee appears to be much higher. For example, for multiligament injuries of the knee joint, an incidence of symptomatic HO between 26% and 45% has been reported [5]. To our knowledge, no previous systematic or scoping review has specifically evaluated prophylactic interventions to prevent HO around the knee, and the available evidence appears fragmented across clinical contexts and prophylactic modalities. We therefore conducted a scoping review to systematically map this evidence and address three main questions:

i) Which indications are reported for HO around the knee?
ii) Which interventions are used to prevent HO around the knee?
iii) Which outcomes are reported to assess the effectiveness of the prophylaxis methods used?

In addition, we aimed to characterize reported intervention regimens, including dose, schedule, duration, and timing.

## Methods

### Protocol and search string

This scoping review was conducted in accordance with the Preferred Reporting Items for Systematic Reviews and Meta-analysis (PRISMA) extension for scoping reviews (PRISMA-ScR) guidelines [6]. This review followed an analysis protocol that was prospectively developed based on the Joanna Briggs Institute (JBI) methodological recommendations for scoping reviews [7] and the PRISMA-P template [8]. MEDLINE via PubMed (National Library of Medicine), Embase (Elsevier), the Cochrane Library (Wiley), and the Web of Science Core Collection (Clarivate) were systematically searched. To identify current trials, protocols, and grey literature, additional searches were conducted using ClinicalTrials.gov, the World Health Organization (WHO) International Clinical Trials Registry Platform (ICTRP), Google Scholar, and ProQuest Dissertations and Theses Open. The search strategy was developed around the broad combination of HO AND knee for maximum sensitivity, using MeSH terms and all identified synonyms. Each search string was independently evaluated by two authors (MR and GW) according to the Peer Review of Electronic Search Strategies (PRESS) Guidelines [9]. The completed search strings for each database, as well as source-specific search details, are provided in Supplement S1. We did not apply any language restrictions. The process of de-duplication and title and abstract screening was performed by one reviewer (MR), and all records and screening decisions were subsequently reviewed by a second reviewer (GW). Full-text screening was independently performed by two reviewers (MR and GW). Disagreements at either stage were resolved through discussion. If consensus could not be reached, a third reviewer (KP) was consulted. Additionally, reference checking of all retrieved full-text articles was employed.

### Selection criteria

Our selection criteria focused on maximum sensitivity to include all available evidence. We included all types of research and grey literature. For studies reporting patient data, we included studies in which at least 75% of the participants were adults, and that directly evaluated a predefined, modifiable intervention or treatment strategy in relation to the development or recurrence of HO around the knee, irrespective of modality or the strategy’s primary clinical indication. Exploratory studies assessing multiple potential risk factors without a predefined comparison of alternative treatment strategies were summarized separately. The complete inclusion criteria are provided in the protocol and summarized in a tabular format in Supplement S2.

### Data extraction

Data extraction was performed in duplicate (MR and GW) using a standardized extraction template. Sources containing expert recommendations were summarized descriptively in a separate table. The characteristics of the identified literature reporting patient data were summarized by prophylaxis modality. We categorized modalities based on our findings as continuous passive motion (CPM), pharmacological prophylaxis, radiotherapy (RT), surgical techniques, and combination therapy. We considered predefined intraoperative technical measures or alternative surgical approaches as potential prophylactic strategies when the studies directly compared strategy-specific HO outcomes. If two or more modalities were applied concurrently, we extracted data for each modality where available and classified the study under combination therapy. If modalities were applied sequentially (e.g., one for primary prophylaxis and another later on for recurrence prophylaxis), we extracted data for each intervention separately and listed the study in each relevant modality category. Any reported HO event was classified as “any HO”, whereas “clinically relevant HO” was restricted to events described as symptomatic, explicitly deemed clinically relevant by the study authors, or those requiring further intervention. Because terminology was inconsistent across the included literature, this classification was partly based on author-reported descriptions and may therefore be subject to misclassification. This operational definition was established before outcome aggregation and applied consistently across all included studies. The need for further intervention due to HO was additionally extracted as a separate outcome. Publications available only as abstracts were flagged using a dedicated extraction marker (‡). When information was reported only for the overall cohort, and not separately for the population of interest, we attempted to derive subgroup-specific values from the available data. If this was not feasible, the values for the whole cohort were extracted and appropriately marked (*). Whole-cohort estimates were retained for descriptive evidence mapping only, but did not contribute to the numerical HO event proportions. A sensitivity analysis restricted the modality-specific summaries to full-text reports.

### Risk of bias assessment

The corresponding JBI levels of evidence were extracted for each study. Risk of bias (RoB) was independently assessed by two authors (MR, GW) using the appropriate JBI critical appraisal tool, and reported conflicts of interest and funding were reviewed. Disagreements were resolved by consensus or, if necessary, by consultation with a third author (KP).

### Statistical analysis

Statistical analyses were conducted using R version 4.5.2 [10] with RStudio version 2025.09.02 as an integrated development environment [11]. We summarized publication characteristics, including year, country, design, and JBI levels of evidence. Participant characteristics were summarized descriptively. Age and follow-up were summarized using one study-level central estimate: the reported mean, the median if no mean was available, or a mean calculated from individual values. The resulting medians and IQRs therefore reflect study-level rather than patient-level distributions. Sex reporting was summarized where extractable. Prophylaxis indications/contexts and prophylaxis intent (e.g., primary vs. recurrence prophylaxis) were mapped descriptively and considered in the narrative interpretation. Given the sparse, heterogeneous, and partly overlapping evidence, no formal subgroup analyses by clinical scenario were performed. Furthermore, regimens were tabulated by modality (timing, duration, dose, and schedule). Crude reported event proportions for any HO, clinically relevant HO, and the need for further intervention due to HO were calculated only for cohorts with extractable, cohort-specific numerators and denominators for the population of interest, and summarized separately. However, studies were considered reporting on an outcome if any information on it was provided, including overall cohort data, not specific for the population of interest. Accordingly, the number of studies reporting outcomes may exceed the number of studies contributing to the crude event proportions. These descriptive aggregates did not involve study-level weighting, variance estimation, confidence interval calculation, or modelling of between-study heterogeneity and should not be interpreted as meta-analytic pooled estimates or used for direct comparisons between modalities.

## Results

### Review statistics

**Fig. 1:**
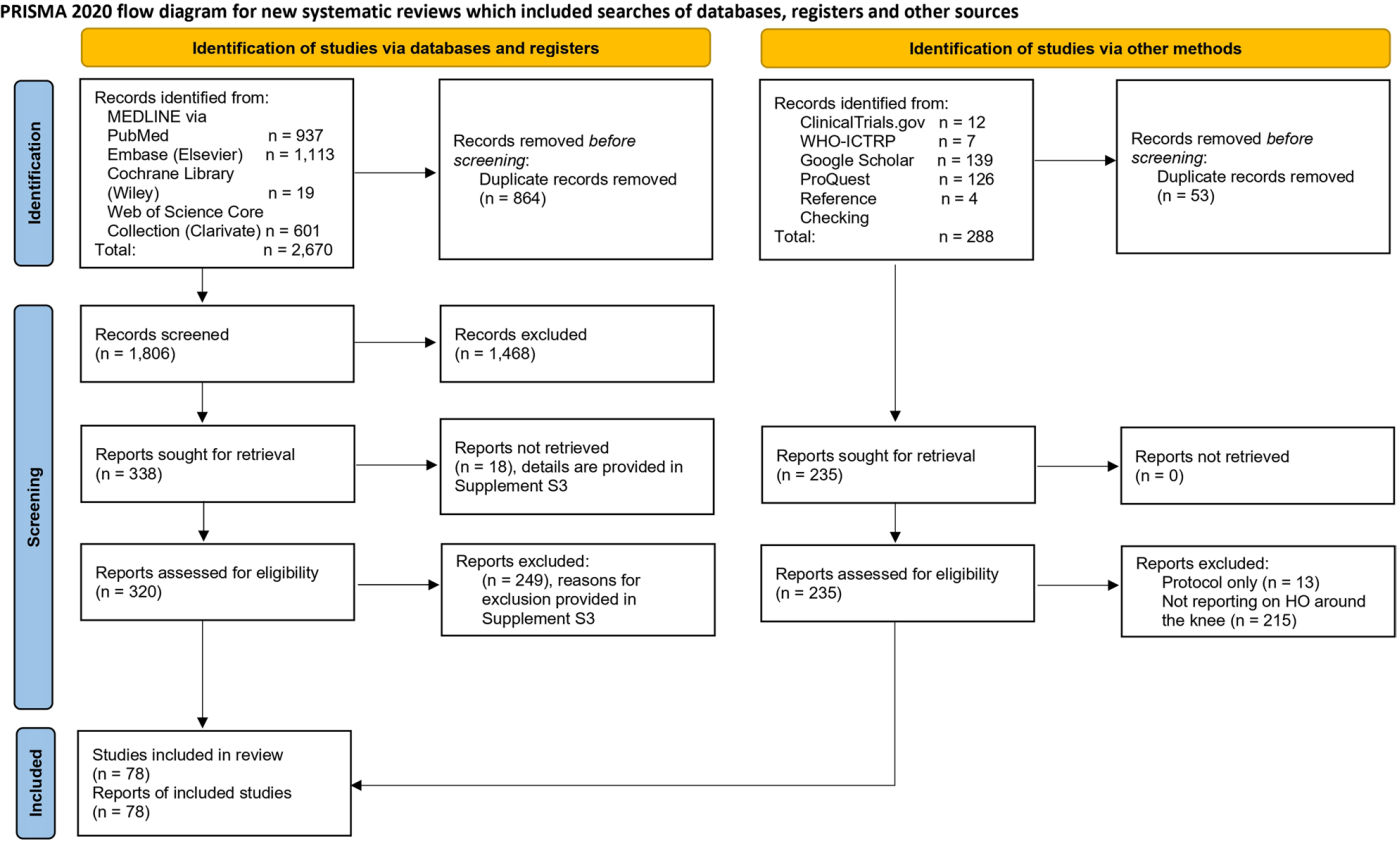
PRISMA 2020 flow diagram of study selection. The flow diagram summarizes the identification, deduplication, screening, eligibility assessment, and final inclusion of publications reporting on prophylactic interventions for heterotopic ossification around the knee. Abbreviations: PRISMA: Preferred Reporting Items for Systematic Reviews and Meta-Analyses; WHO-ICTRP: World Health Organization International Clinical Trials Registry Platform. From: Page MJ, McKenzie JE, Bossuyt PM, Boutron I, Hoffmann TC, Mulrow CD, et al. The PRISMA 2020 statement: an updated guideline for reporting systematic reviews. BMJ 2021;372:n71. doi: 10.1136/bmj.n71. For more information, visit: http://www.prisma-statement.org/

We conducted a systematic search on January 31, 2026, and updated it on March 18, 2026. Our search yielded 2,958 records before deduplication, comprising 2,670 records from the database searches, 284 records from grey-literature sources, and 4 additional records identified through reference checking. The entire selection process is summarized as a PRISMA flowchart [12], presented in Fig. 1. All of the studies that were excluded during full-text screening and the reasons for exclusion, as well as studies that could not be retrieved, are summarized in Supplement S3. If we could not identify or retrieve the full-text article, we also included abstract-only publications. In the end, we identified 18 publications reporting expert opinions and 62 publications reporting on 3321 patients, of whom 1579 received the evaluated interventions or treatment strategies. Concerning the study design, these 62 publications comprised 1 RCT [13], 2 comparative cohort studies [14, 15], 1 observational cohort study [16], 2 case-control studies [17, 18], 19 case series [19–37], and 37 case reports [38–74]. Two of the identified publications are abstract-only reports [25, 26]. 

**Fig. 2:**
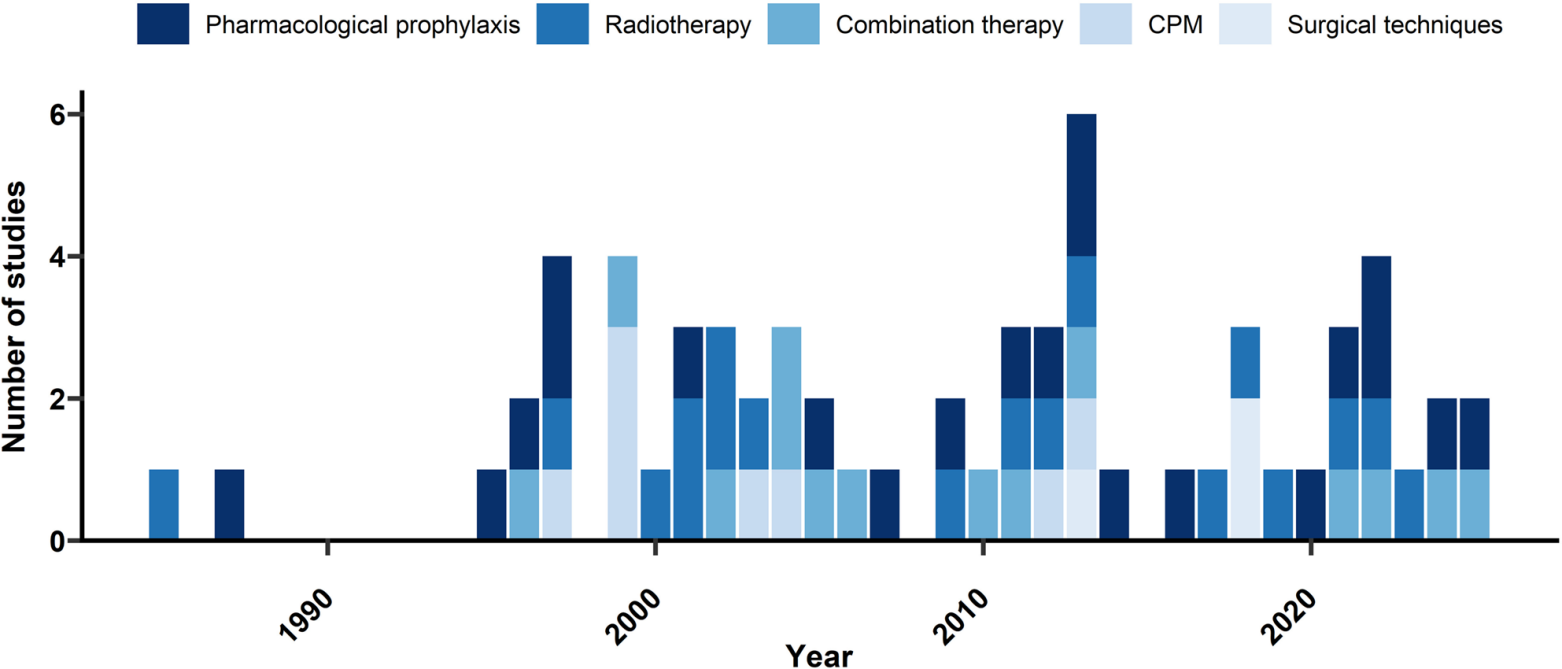
Number of included studies by publication year and prophylactic modality. Stacked bar chart showing the temporal distribution of included publications according to prophylactic modality. Abbreviations: CPM, continuous passive motion

**Fig. 3:**
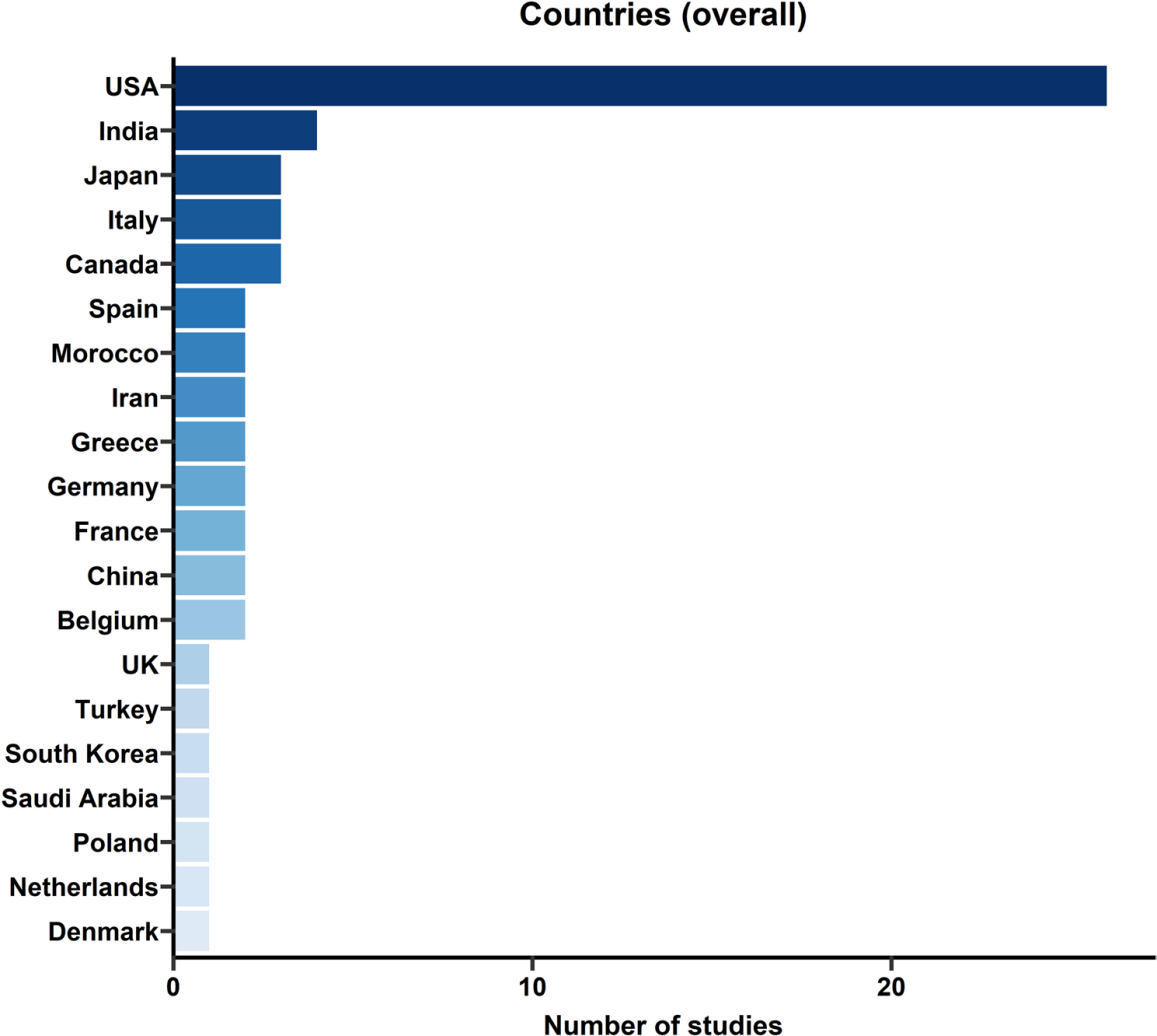
Countries of origin of included studies. Horizontal bar chart showing the geographic distribution of included publications by country. The bars indicate the total number of studies contributed by each country across all prophylactic modalities

**Fig. 4:**
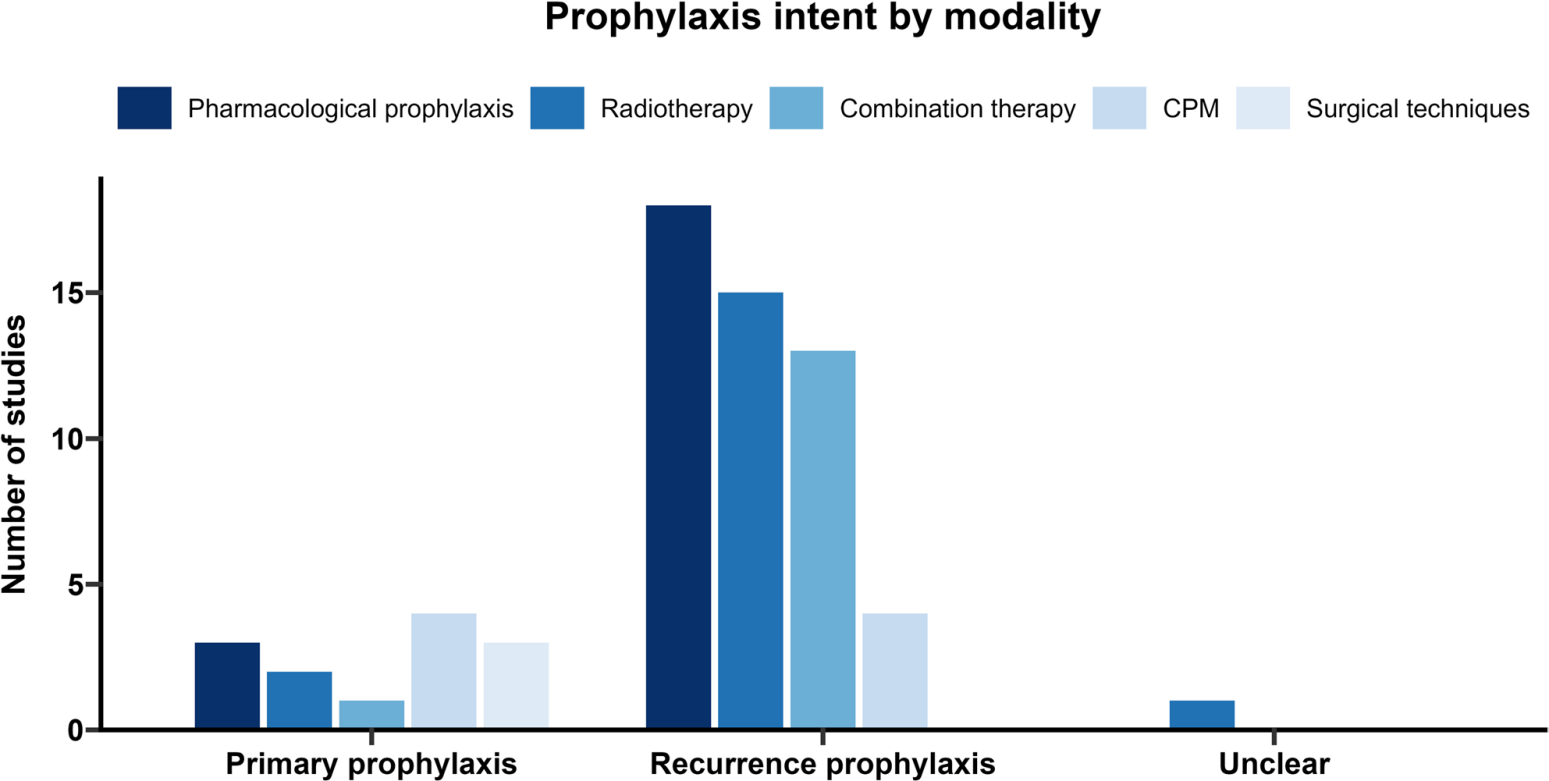
Prophylaxis intent by modality. Bar chart showing the reported intent of prophylaxis across modalities, categorized as primary prophylaxis, recurrence prophylaxis, or unclear indication. Abbreviations: CPM, continuous passive motion

**Fig. 5:**
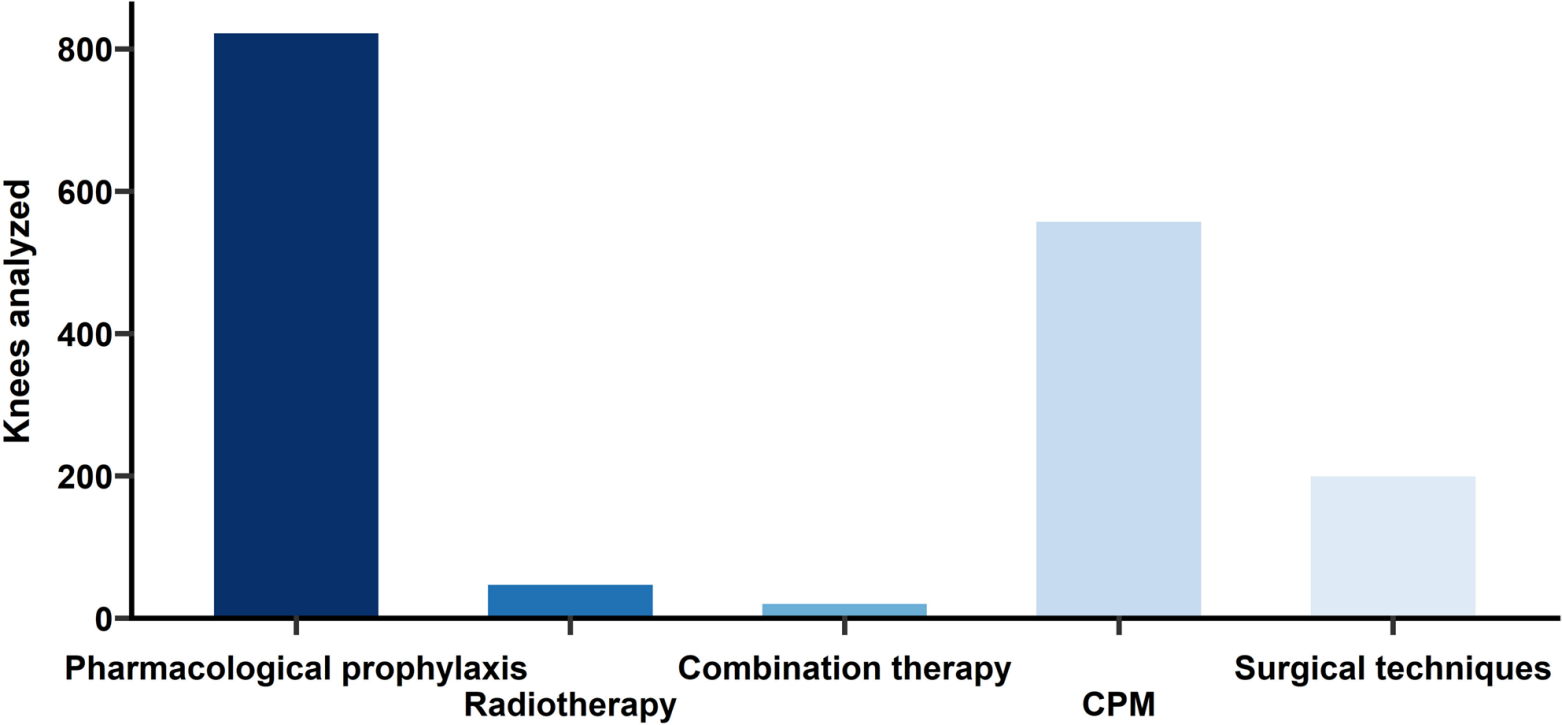
Number of knees analyzed by prophylactic modality. Bar chart showing the total number of knees analyzed across included studies for each prophylactic modality. Abbreviations: CPM, continuous passive motion

We identified 8 studies reporting on CPM, including the two abstract-only reports, 21 reporting on pharmacological prophylaxis, 18 reporting on RT, 3 reporting on dedicated surgical techniques, and 14 reporting on combination therapy. Modality counts were not mutually exclusive: Mills et al. [23] contributed to both the CPM and RT categories, and Pham et al. [40] to both the CPM and pharmacological-prophylaxis categories. Thus, the 62 unique clinical publications generated 64 modality assignments. Furthermore, Rader et al. [36] and Stannard et al. [16] were included in both the expert-opinion and clinical evidence syntheses. Overall, 78 unique publications were identified. The number of studies per year of publication is presented in Fig. 2, and the number of studies per country in Fig. 3. The prophylaxis intent and the number of knees analyzed per modality are presented in Figs. 4 and 5, respectively. An overview of the prophylactic strategies identified for HO around the knee and key signals from the included studies is provided in Table 1.

**Table 1:** Overview of prophylactic strategies and key signals from the included studies

Strategy	Studies (design profile and knees analyzed)	Predominant clinical setting	Typical regimen or comparison	Main signal from included studies	Interpretation
CPM	8 studies: 5 case series, 3 case reports 557 knees analyzed	Equal numbers of studies addressed primary and recurrence prophylaxis. The majority of knees were derived from primary arthroplasty cohorts.	Usually started immediately postoperative; duration per day often reported incompletely; used up to 6 weeks	In a 500-knee TKA series, any HO was 76/500 (15.2%), but clinically relevant HO was only 7/500 (1.4%). In a knee dislocation series, HO remained frequent despite CPM (15/36, 41.7% any HO; 6/36, 16.7% clinically relevant HO).	Mostly employed as a rehabilitation adjunct rather than as a stand-alone prophylactic strategy against HO.
Pharmacological prophylaxis	21 studies: 1 comparative cohort study, 7 case series, 13 case reports 822 knees analyzed	Majority of studies: recurrence prophylaxis after HO excision; majority of knees analyzed in one cohort study on primary prophylaxis after TKA	Most often indomethacin; also celecoxib, ibuprofen, ASA, and bisphosphonates (etidronate)	Most small recurrence series reported no or few new HO events. In the largest comparative TKA cohort, ASA-based thromboprophylaxis was associated with lower HO incidence than non-ASA regimens (100/747, 13.4% vs. 56/304, 18.4%).	Most frequently reported non-surgical modality. The majority of studies are small case series and case reports. Comparative evidence only available for ASA after primary TKA.
Radiotherapy	18 studies: 1 case-control, 1 observational cohort, 6 case series, 10 case reports 47 knees analyzed	Predominantly recurrence prophylaxis after HO excision; few reports on primary prophylaxis for high-risk scenarios	Usually single-fraction 7.0 Gy perioperatively or within 72 h postoperative; occasionally fractionated therapy	Low crude reported event proportions for any HO reported. No clinically relevant HO was reported among the 38 knees with extractable data (0/38, 0.0%).	Mostly employed for recurrence prophylaxis, knee-specific efficacy data remain scarce with potentially high risk of publication bias and small sample sizes.
Surgical techniques	3 comparative studies: 1 RCT, 1 case-control study, 1 comparative cohort study 199 knees analyzed	Primary prophylaxis only: after ACL reconstruction, proximal tibia fracture fixation, and intramedullary nailing for floating knee injury	Femoral canal lavage and bone plug removal vs. no measures; external fixation vs. plate fixation; antegrade vs. retrograde femoral nailing	Lower HO rates were reported with lavage (2/130, 1.5% vs. 4/155, 2.6%), external fixation vs. plate fixation (1/62, 1.6% vs. 9/68, 13.2%), and antegrade vs. retrograde femoral nailing (3/7, 42.9% vs. 17/19, 89.5%).	Highest-yield evidence, due to the availability of comparative studies. However, surgical scenarios are heterogeneous and certain techniques increase the risk for adverse events.
Combined prophylaxis	14 studies: 2 case series, 12 case reports 20 knees analyzed	Almost exclusively recurrence prophylaxis after HO excision	Combinations included RT + NSAID, NSAID + bisphosphonate, and CPM + NSAID + RT	No new HO was reported among the small number of knees with extractable data. Functional outcomes were inconsistently reported.	Mostly employed for recurrence prophylaxis. Very limited, anecdotal evidence that is highly susceptible to selective reporting and publication bias.

This table summarizes the main prophylactic strategies identified in the scoping review. For each strategy, the table reports the number and design profile of included studies, the number of knees analyzed, the predominant clinical setting, the typical regimen or comparison, the main signal from the included studies, and the overall interpretation. Detailed study-level characteristics and outcome data are provided in the Supplement

*Abbreviations*: *ACL* anterior cruciate ligament, *ASA* acetylsalicylic acid, *CPM* continuous passive motion, *Gy* gray, *HO* heterotopic ossification, *NSAID* non-steroidal anti-inflammatory drug, *RCT* randomized controlled trial, *ROM* range of motion, *RT* radiotherapy, *TKA* total knee arthroplasty

### Expert opinion

We identified 18 expert-opinion sources. Recommendations on HO prophylaxis were largely risk-adapted. Routine arthroplasty was considered not to require prophylaxis in 3/3 (100%) of the sources reporting on the issue [75–77]. Primary prophylaxis was suggested for high-risk scenarios (e.g., open knee dislocation, multiligament injury, extensive debridement, hypertrophic arthrosis, periosteal damage, and prior HO in another joint) in 11/12 studies (91.7%) addressing the matter [3, 5, 16, 36, 75, 77–83], with one advocating against it [84]. Recurrence prophylaxis after HO resection was endorsed, with varying strength, in 10/10 (100%) articles reporting on the issue [5, 16, 75, 76, 78, 79, 81, 85–87]. The most commonly recommended modality for HO prophylaxis was non-steroidal anti-inflammatory drugs (NSAIDs) in 15/18 (83.3%) articles [3, 5, 16, 75, 76, 78–83, 85–88], with indomethacin as the preferred option in 10/18 (55.6%) of the studies [5, 16, 75, 76, 78, 79, 81, 85–87], and cyclooxygenase-2 inhibitors recommended as an alternative in 1/18 articles (5.6%) [79]. RT was recommended as an option in 12/18 publications (66.7%) [3, 16, 75, 76, 78–81, 83, 85, 87, 88], with 2/18 (11.1%) [3, 75] of the sources proposing combined RT+NSAID for recurrence prophylaxis. CPM was primarily described as an adjunct to maintain ROM rather than to prevent recurrence. CPM was recommended in 2/18 (11.1%) [85, 86] of the articles. Some authors also described surgical technique modifications (e.g., thorough irrigation, minimizing periosteal trauma) as potential preventive strategies [80]. The key outcomes addressed in these texts were prevention of HO and maintenance of ROM and function. Potential harms of these interventions were discussed. These included: NSAID-related bleeding, gastrointestinal and renal effects, as well as RT-related wound-healing issues, gonadal toxicity, and potential secondary malignancy risk. Formal evidence grading was usually absent, and recommendations were frequently extrapolated from hip literature. These expert-opinion sources therefore provide useful clinical context, but only limited direct knee-specific evidence (Supplement S4).

### CPM

We identified 8 studies comprising 5 case series [23, 25–28] and 3 case reports [38–40] reporting on the use of CPM in 557 knees of 508 patients. All CPM evidence was observational and descriptive, spanning publications from 1997 [40] to 2013 [26]. The participant age was reported in 7 studies (median 38.5 years, IQR 31.6–60.3), and follow-up was reported in 6 studies (median 21.0 months, IQR 13.5–31.5). Sex was reported in 6 studies with 198/492 (40.2%) males. The reported indication was primary prophylaxis after arthroplasty in 3/8 studies (37.5%) with 467 participants/502 knees, recurrence prophylaxis of neurogenic HO in 3 studies (37.5%) with ≥ 4 participants/≥17 knees, as well as primary prophylaxis after knee dislocation (35 participants/ 36 knees) and recurrence prophylaxis of posttraumatic HO (2 participants/2 knees) in one study each. Additional information is available in Supplement S5. Reporting of CPM regimen details was limited. Information regarding the duration of CPM per day was available in 2 studies (25.0%), and regarding the timing and duration of CPM in 4 studies (50.0%), but parameter reporting was incomplete. In 7/8 studies (87.5%), CPM was used in conjunction with physical therapy. The occurrence of HO was reported for a total of 544/557 (97.7%) knees, with any HO reported in 93/544 (17.1%) and clinically relevant HO in 15/544 (2.8%). A total of 16/544 knees (2.9%) needed further intervention due to HO. The ROM was assessed in 7/8 studies (87.5%), but only 5/8 studies (62.5%) provided pre- and postoperative values. Return to work/activity was assessed in 5/8 studies (62.5%), pain was assessed in 2/8 studies (25%), and none of the studies assessed PROMs. Further information is available in Supplement S6. All evidence regarding CPM was observational and descriptive. No comparative studies evaluating CPM effectiveness were identified.

### Pharmacological prophylaxis

We identified 21 studies (1 comparative cohort study [15], 7 case series [31–37], and 13 case reports [40, 53–64]) reporting on pharmacological prophylaxis for HO around the knee, comprising 822 knees in 805 patients. The earliest report was published in 1987, and the most recent in 2025. Age and follow-up were both reported in 21/21 studies (100%), with a median age of 35.2 years (IQR 30.8–52.0), and a median follow-up of 12.0 months (IQR 6.0–21.4). Sex was reported for 798/805 patients (99.1%), with ≥ 341/798 (≥ 42.7%) males. The indication was recurrence prophylaxis after HO excision in 18/21 studies (85.7%), whereas primary prophylaxis (mostly after TKA) was reported in 3/21 studies (14.3%). Further details are provided in Supplement S7.

NSAID-based prophylaxis was used in 18/21 studies (85.7%). The most commonly used drug was indomethacin in 14/21 studies (66.7%), while celecoxib was used in 2/21 (9.5%) studies, and ibuprofen and acetylsalicylic acid (ASA) were used in 1/21 studies (4.8%) each. Bisphosphonates (etidronate) were used in 3/21 studies (14.3%). The dose schedules were generally heterogeneous and incompletely reported. Prophylaxis was administered postoperatively in 19/21 studies (90.5%), although in 3 of these studies, therapy was initiated preoperatively with postoperative continuation. The duration of the pharmacological prophylaxis ranged from 2 weeks up to 11 months, with 14/21 studies (66.7%) reporting a duration ≤ 2 months. Physical therapy was a common co-intervention, used in 10/21 studies (47.6%), and in 3/21 studies (14.3%), a subgroup of patients received additional RT. Because outcome reporting varied across studies, the number of knees contributing to each endpoint differed. Any HO outcomes were reported in 18/21 studies (85.7%), with any HO reported in 104/796 knees (13.1%) with available data. However, this estimate was strongly influenced by one cohort study [15] that reported on 747 patients receiving ASA. Although ASA was administered for venous thromboembolism prophylaxis, the study was included because its explicit objective was to evaluate whether ASA influenced HO formation. This study reported that patients who received ASA were less likely to develop HO than patients who received other drugs for venous thromboembolism prophylaxis (13.4% vs. 18.4%; *p* = 0.047). Clinically relevant HO and HO requiring further intervention were reported in 18/21 studies (85.7%) each, and were observed in 3/50 (6.0%) and 2/50 (4.0%) knees, respectively. ROM was reported in 17/21 studies (81.0%), whereas pain and return to work/activity were each assessed in 12/21 (57.1%) studies. However, these outcomes were frequently incompletely reported and often lacking paired pre- and postoperative values. PROMs were reported in 5/21 studies (23.8%), but only 3/21 studies (14.3%) used standardized instruments. Instruments used included the Hospital for Special Surgery score (HSS), International Knee Documentation Committee score (IKDC), Knee Society Score (KSS), Lysholm score, and Tegner activity levels. A statement on AEs was reported in 8/21 studies (38.1%) and included, most commonly, wound infections and hematoma. One case report described elevated blood pressure leading to early termination of indomethacin. Additional information is provided in Supplement S8.

### Radiotherapy

We identified 18 studies (1 observational cohort study [16], 1 case-control study [18], 6 case series [19–24], and 10 case reports [65–74]) reporting on the use of RT for HO prophylaxis around the knee. These studies comprised 48 knees of 46 patients, with 47/48 knees (97.9%) analyzed. RT evidence was limited to very small cohorts and case-based reports, predominantly in recurrence prophylaxis settings. Age was reported in 16/18 studies (88.9%) with a median age of 43.6 years (IQR 35.5–52.0), and follow-up was reported in 12/18 studies (66.7%) with a median of 12.4 months (IQR 9.0–18.9). Sex was reported for the population of interest in 12 studies with 16/27 (59.3%) males. The reported indication was predominantly recurrence prophylaxis after HO excision in 15/18 studies (83.3%), whereas primary prophylaxis was reported in 2/18 studies (11.1%), and 1/18 studies (5.6%) did not clearly report the indication. Further details are provided in Supplement S9. RT was delivered as a single-fraction treatment in 10/18 studies (55.6%), most commonly 1 × 7.0 Gy (Gy) (8/18, 44.4%), and was delivered postoperatively in 13/18 studies (72.2%), typically on postoperative day 1 or within 72 h. Preoperative RT was used in 1/18 studies (5.6%), and fractionated schedules (e.g., 10 × 2.0 Gy, 2 × 5.0 Gy, 3 × 7.0 Gy) were used in 4/18 studies (22.2%). In 3/18 studies (16.7%) RT was combined with other interventions like physical therapy. Because outcome reporting varied across studies, the number of knees contributing to each endpoint differed. The occurrence of any HO was reported in 8/18 studies (44.4%), with 2/24 analyzed knees (8.3%) developing HO after RT (the occurrence of HO was not reported for 2 knees in Chidel, 2001) [20]. However, clinically relevant HO and HO requiring further intervention both occurred in 0/38 knees (0.0%) in 13 studies reporting on the issue, with 12 providing information specific for the population of interest. ROM was reported in 11/18 studies (61.1%), but pre- and postoperative ROM values were inconsistently provided. Pain was reported in 3/18 studies (16.7%), and return to work/activity was reported in 2/18 (11.1%) studies, but none of the studies used standardized instruments for the assessment. Information on AEs potentially related to prophylaxis was reported in 9/18 studies (50.0%) and included mostly cases of wound infection. However, one study reported a case of a fatal, potentially RT-induced sarcoma approximately 5 years after RT. Additional information is provided in Supplement S10.

### Surgical techniques

We identified 3 comparative studies (1 RCT [13], 1 comparative cohort study [14], and 1 case-control study [17]) evaluating surgical techniques in relation to HO risk around the knee. Although primarily selected for fracture management, the approaches evaluated by Kent et al. [14] and Berven et al. [17] were retained because both studies directly compared predefined surgical strategies and reported strategy-specific HO outcomes. We identified 3 additional studies [89–91] that did not report on HO prophylaxis, but rather on risk factors for HO during surgery. All three were excluded from primary synthesis. Summarized information on these studies is available in Supplement S11 and S12. Across studies, 464 participants were enrolled. Surgical techniques classified within this modality were employed in 208 participants, and 199/208 knees (95.7%) in these intervention groups were included in the analysis. The earliest report was published in 2013, and the most recent in 2018. Age and follow-up were reported in 3/3 studies (100%), while sex was reported in 2/3 studies (66.7%). All studies addressed primary prophylaxis in certain high-risk scenarios. Further details are provided in Supplement S13.

The evaluated techniques were heterogeneous and context-specific. The RCT by Bhandary et al. [13] evaluated intraoperative preventive measures during anterior cruciate ligament (ACL) reconstruction (copious femoral canal lavage after reaming, meticulous hemostasis, and removal/nibbling of excessive femoral bone plug) vs. no preventive measures in 285 patients. HO was reported in 2/130 patients (1.5%) receiving preventive measures and in 4/155 patients (2.6%) receiving no preventive measures. The original authors described this difference as significant [13]. However, no statistical test or p-value was reported. Kent et al. [14] conducted a cohort study comparing antegrade and retrograde femoral nailing after floating knee injury in 25 patients. Retrograde femoral nailing resulted in a significantly higher HO prevalence (*p* = 0.028) and severity (*p* = 0.004) than antegrade nailing, but there was no significant difference regarding knee ROM (*p* = 0.439) between the two groups [14]. The case-control study by Berven et al. [17] compared external fixation using an Ilizarov frame to internal fixation with locking plates in 154 patients with proximal tibia fractures with a complete metaphyseal component. External fixation resulted in a significantly lower HO prevalence (*p* = 0.013), but there was no significant difference regarding postoperative ROM. Furthermore, external fixation resulted in a higher rate of superficial infections (40.4% vs. 2.9%, *p* = 0.000) and a longer time of healing (*p* = 0.041) [17]. Additional information is presented in Supplement S14.

### Combined approaches

We identified 14 studies (2 case series [29, 30] and 12 case reports [41–52]) reporting on combined prophylaxis strategies for HO around the knee, comprising 20 knees in 16 patients. Combined prophylaxis was almost exclusively described in small case-based reports, mainly for recurrence prophylaxis after HO excision. Age and follow-up were reported in 14/14 studies (100%), with a median age of 40.5 years (IQR 32.8–51.3) and a median follow-up of 18.2 months (IQR 9.0–33.0). Sex was reported for 13/16 patients (81.3%), with 8/13 (61.5%) males. The indication was recurrence prophylaxis after HO excision in 13/14 studies (92.9%), predominantly following excision of neurogenic HO, whereas primary prophylaxis at the time of primary TKA in a high-risk patient was described in 1/14 (7.1%) studies. Further details are provided in Supplement S15.

All combined prophylaxis regimens contained pharmacologic interventions. NSAIDs were used in 14/14 studies (100%), most commonly indomethacin (11/14, 78.6%), while other NSAIDs included ibuprofen (1/14, 7.1%) and loxoprofen (1/14, 7.1%). In one report, the NSAID used was not specified. Bisphosphonates (mainly etidronate) were included in 6/14 studies (42.9%), and continuous passive motion (CPM) in 4/14 studies (28.6%). RT was used in 6/14 studies (42.9%), typically as single-fraction perioperative RT of 7–8 Gy delivered postoperatively in 3/6 (50.0%) studies using RT or preoperatively in 2/6 studies (33.3%). One study did not report whether RT was applied pre- or postoperatively. The most frequent dual combinations were NSAID + bisphosphonate (5/14, 35.7%), NSAID + RT (4/14, 28.6%), and NSAID + CPM (3/14, 21.4%). Triple-modality strategies were used in 2/14 studies (14.3%), with CPM + NSAID + RT in 1 study and bisphosphonate + NSAID + RT in 1 study each. Because outcome reporting varied across studies, the number of knees contributing to each endpoint differed. Any new HO was reported in 10/14 studies (71.4%), with 9/14 studies (64.3%) providing specific information for the population of interest. No HO was reported among the 11 knees with available population-specific data (0/11). Clinically relevant HO and the need for further interventions were both reported in 13/14 studies (92.9%). Clinically relevant HO developed in 0/15 knees (0%), and 0/18 knees (0%) required further interventions due to HO. ROM was assessed in 14/14 studies (100%), albeit incompletely reported in one case. PROMs were reported in 3/14 (21.4%) studies, with only 1/14 (7.1%) using a standardized instrument (IKDC). Pain and return to work/activity were reported in 10/14 (71.4%) and 9/14 (64.3%) studies, respectively, but often without complete paired pre–post data. A statement on AEs was included in 4/14 studies (28.6%). Additional information is provided in Supplement S16.

### Evidence base and reporting limitations

Overall, the evidence base was dominated by observational and descriptive reports. Comparative studies were mainly available for surgical techniques. For the use of CPM and combined approaches, only case series and case reports were identified. The RoB assessment identified recurrent methodological limitations, including: unclear randomization, blinding, follow-up, and statistical reporting in the single RCT; confounding, selection bias, and incomplete follow-up in comparative observational studies; and unclear consecutive or complete inclusion in several case series. Case reports most frequently showed gaps in intervention, outcome, or adverse-event reporting. No notable concerns arose from disclosed conflicts of interest or funding. Detailed results of the risk-of-bias assessment are provided in Supplement S17. Considerable heterogeneity was present regarding clinical scenarios, prophylactic regimens, and outcome assessment. Reporting of key endpoints was frequently incomplete, particularly for paired pre- and post-intervention ROM values and AEs, limiting cross-study comparability. Standardized instruments for the assessment of PROMs were only used in a minority of studies. This limitation was compounded by incomplete denominator reporting and by the need to infer subgroup-specific data in some studies. A modality-specific summary of HO requiring further intervention is provided in Supplement S18 and a sensitivity analysis restricted to full-text reports in Supplement S19.

## Discussion

This scoping review provides, to our knowledge, the first systematic evidence map of prophylactic interventions specifically for HO around the knee, integrating a fragmented literature across five prophylactic modalities and diverse clinical contexts. Three overarching findings emerged. First, the evidence base is dominated by descriptive reports, with only a single RCT and a few comparative observational studies identified. Second, indications for HO prophylaxis in the identified studies largely mirrored the identified expert recommendations. Most clinical reports addressed recurrence prophylaxis after HO excision. Primary prophylaxis was largely employed in high-risk scenarios only. Third, outcome reporting was heterogeneous and frequently incomplete, lacking standardization. Paired pre- and postoperative ROM values, PROMs evaluated with standardized instruments, and systematic reporting of AEs were particularly sparse. Hence, cross-study comparability is limited, and the available literature does not support robust conclusions regarding relative effectiveness across modalities.

NSAID-based prophylaxis was the most frequently reported pharmacological approach, mainly using indomethacin, typically administered postoperatively, yet dosing and duration were inconsistently described. RT was primarily used for recurrence prophylaxis and most commonly delivered as a single-fraction perioperative treatment. CPM was usually applied as an adjunct to rehabilitation and physical therapy, with limited reporting of device parameters and daily duration. Combined approaches were almost exclusively reported in small case-based literature, predominantly for recurrence prophylaxis following the excision of neurogenic HO. Although no new HO was reported, the small sample sizes and the high potential for selective reporting and publication bias mean that the absence of new HO cannot be interpreted as reliable evidence for high efficacy. Comparative evidence was most readily available for surgical technique modifications. These studies reported strategy-specific differences in HO rates in selected high-risk contexts. The single reported fatal, potentially RT-induced sarcoma represents an important safety signal. However, this isolated report does not permit estimation of the incidence of radiation-induced malignancy after RT for HO prophylaxis or establish a causal association.

Clinical applicability depended strongly on the underlying setting. Evidence specifically addressing dedicated HO prophylaxis in unselected primary TKA was limited, and expert sources generally did not advocate routine prophylaxis. Primary prophylaxis was otherwise concentrated in selected high-risk settings, including trauma and reconstructive surgery, as well as arthroplasty patients with additional risk factors. Recurrence prophylaxis after HO excision was the dominant context in the pharmacological, RT, and combined-prophylaxis literature, with post-traumatic and neurogenic HO representing prominent and partly overlapping subgroups. These clinical contexts are not interchangeable. Modality-specific findings and crude event proportions should therefore be interpreted within the respective indication and population rather than compared across settings.

Several limitations of the current scoping review need to be mentioned. The underlying studies were clinically heterogeneous with respect to etiology, index procedures, and prophylaxis intent. Outcomes were variably defined, often incompletely reported, and the literature mainly comprised descriptive study types. Furthermore, denominators for the subgroup of interest were not always available. Given the paucity of comparative trials, the diversity of study designs, and the limited reporting of relevant outcomes, quantitative meta-analysis was not feasible. The design-specific risk-of-bias assessment additionally identified methodological limitations that further constrain interpretation of the available evidence. Additionally, the distinction between dedicated HO prophylaxis and modifiable treatment strategies primarily used for other clinical indications was not always clear. Consistent with the broad scope of the review, we included predefined strategies that were directly evaluated in relation to HO formation. However, this classification was partly judgment-based, and the resulting observational comparisons should not be interpreted as evidence of causal preventive effectiveness.

Future research should prioritize knee-specific prospective multicenter registries and comparative evaluations in clearly defined high-risk populations, ideally through pragmatic randomized trials including a no-prophylaxis or usual-care control arm. Future studies should standardize HO assessment, clearly distinguish primary from recurrence prophylaxis, report paired pre- and post-intervention ROM values and other functional outcomes, assess PROMs using validated instruments, and use a standardized approach to assess and report AEs. Such harmonization would enable meaningful cross-study comparisons, allowing evidence-based recommendations tailored to the knee, reducing the need to extrapolate from hip literature.

## Conclusion

This scoping review mapped the available literature on prophylactic interventions to prevent HO around the knee and found that the evidence base is dominated by descriptive reports with few comparative studies, and that regimen details and outcome reporting are frequently incomplete. Overall, the available literature suggests that HO prophylaxis around the knee is mainly considered in selected high-risk primary settings and for recurrence prevention after HO excision. Routine prophylaxis after arthroplasty was generally not advocated in expert opinion sources and rarely evaluated outside selected higher-risk populations. Future research should prioritize knee-specific comparative studies with standardized outcome definitions and systematic AE reporting in selected high-risk primary settings and for recurrence prevention.

## Supplementary Information

Supplementary Material 1.

Supplementary Material 2.

Supplementary Material 3.

Supplementary Material 4.

Supplementary Material 5.

Supplementary Material 6.

Supplementary Material 7.

Supplementary Material 8.

Supplementary Material 9.

Supplementary Material 10.

Supplementary Material 11.

Supplementary Material 12.

Supplementary Material 13.

Supplementary Material 14.

Supplementary Material 15.

Supplementary Material 16.

Supplementary Material 17.

Supplementary Material 18.

Supplementary Material 19.

## Data Availability

All data generated or analysed during this study are included in this published article and its supplementary information files.

## Abbreviations

ACL: Anterior cruciate ligament
AE: Adverse event
ASA: Acetylsalicylic acid
CPM: Continuous passive motion
Gy: Gray
ICTRP: International Clinical Trials Registry Platform
IQR: Interquartile range
HO: Heterotopic ossification
JBI: Joanna Briggs Institute
NSAID: Non-steroidal anti-inflammatory drug
PRESS: Peer Review of Electronic Search Strategies
PRISMA: Preferred Reporting Items for Systematic Reviews and Meta-Analyses
PRISMA-ScR: PRISMA extension for scoping reviews
PROMs: Patient-reported outcome measure
RCT: Randomized controlled trial
RoB: Risk of bias
ROM: Range of motion
RT: Radiotherapy
THA: Total hip arthroplasty
TKA: Total knee arthroplasty
WHO: World Health Organization
