# Supplementary material for: The prevention of heterotopic ossification around the knee: a scoping review
Source: BMC Musculoskelet Disord. 2026 Aug 1;27:651. doi: 10.1186/s12891-026-10318-w (PMC13428452; doi:10.1186/s12891-026-10318-w)
Supplement: Supplementary file 1 — Supplementary Material 1. [file 12891_2026_10318_MOESM1_ESM.docx]

**Supplement S1:** complete search strategy:

| **Source** | **Platform/provider** | **Initial search** | **Updated search** | **Ordering and stopping rule** |
| --- | --- | --- | --- | --- |
| PubMed | National Library of Medicine | January 31, 2026 | March 18, 2026 | All retrieved records exported |
| Embase | Elsevier | January 31, 2026 | March 18, 2026 | All retrieved records exported |
| Cochrane Library | Wiley | January 31, 2026 | March 18, 2026 | All retrieved records exported |
| Web of Science Core Collection | Clarivate | January 31, 2026 | March 18, 2026 | All retrieved records exported |
| Google Scholar | Google | January 31, 2026 | March 18, 2026 | Ordered by relevance; all retrieved results were screened. |
| ProQuest Dissertations & Theses Open | ProQuest | January 31, 2026 | March 18, 2026 | Ordered by relevance; all retrieved results were screened. |
| ClinicalTrials.gov | National Library of Medicine | January 31, 2026 | March 18, 2026 | All retrieved records screened |
| WHO ICTRP | World Health Organization | January 31, 2026 | March 18, 2026 | All retrieved records screened |

**MEDLINE via PubMed (National Library of Medicine): 937 results**

(

"Ossification, Heterotopic"[Mesh]

OR heterotopic ossifica*[tiab]

OR ossification heterotopic[tiab]

OR "heterotopic bone"[tiab]

OR "Ossification, Ectopic"[tiab]

OR "Ossification, Pathologic"[tiab]

OR "Pathological Ossification"[tiab]

OR "Pathologic Ossification"[tiab]

OR "Ectopic Ossification"[tiab]

OR (ectopic[tiab] AND (ossification[tiab] OR "bone formation"[tiab] OR osteogenesis[tiab]))

OR "Myositis Ossificans"[Mesh]

OR "myositis ossificans"[tiab]

OR paraosteoarthropath*[tiab]

OR ((HO[tiab] OR "H.O."[tiab]) AND (ossification[tiab] OR bone[tiab]))

OR (calcif*[tiab] AND (heterotopic[tiab] OR ectopic[tiab] OR ossifica*[tiab] OR ossification[tiab] OR "bone formation"[tiab] OR "myositis ossificans"[tiab]))

OR (calcin*[tiab] AND (heterotopic[tiab] OR ectopic[tiab] OR ossifica*[tiab] OR ossification[tiab] OR "bone formation"[tiab] OR "myositis ossificans"[tiab]))

)

AND

(

"Knee"[Mesh]

OR "Knee Joint"[Mesh]

OR knee[tiab]

OR knee*[tiab]

OR tibiofemoral[tiab]

OR patellofemoral[tiab]

OR patell*[tiab]

OR "Joint, Knee"[tiab]

OR "Knee Joints"[tiab]

OR "Superior Tibiofibular Joint"[tiab]

OR "Superior Tibiofibular Joints"[tiab]

OR "Tibiofibular Joint, Superior"[tiab]

OR "Proximal Tibiofibular Joint"[tiab]

OR "Arthroplasty, Replacement, Knee"[Mesh]

OR (arthroplast*[tiab] AND knee[tiab])

OR (replace*[tiab] AND knee[tiab])

OR TKA[tiab]

OR TKR[tiab]

OR UKA[tiab]

OR UKR[tiab]

)

**Web of Science Core Collection (Clarivate): 601 results**

TS=(

"heterotopic ossification"

OR heterotopic ossifica*

OR "ossification heterotopic"

OR "heterotopic bone"

OR "ectopic ossification"

OR "pathologic ossification"

OR "pathological ossification"

OR "myositis ossificans"

OR paraosteoarthropath*

OR ((HO OR "H.O.") AND (ossification OR bone))

OR (calcif* AND (heterotopic OR ectopic OR ossifica* OR ossification OR "bone formation" OR osteogenesis OR "myositis ossificans"))

OR (calcin* AND (heterotopic OR ectopic OR ossifica* OR ossification OR "bone formation" OR osteogenesis OR "myositis ossificans"))

)

AND

TS=(

knee

OR "knee joint"

OR "knee joints"

OR "joint knee"

OR tibiofemoral

OR patellofemoral

OR patell*

OR "superior tibiofibular joint"

OR "superior tibiofibular joints"

OR "tibiofibular joint superior"

OR "proximal tibiofibular joint"

OR "knee arthroplasty"

OR (arthroplast* AND knee)

OR (replace* AND knee)

OR TKA OR TKR OR UKA OR UKR

)

**Cochrane Library (Wiley): 19 results**

(

[mh "Ossification, Heterotopic"]

OR ("heterotopic" NEXT ossifica*):ti,ab,kw

OR "ossification heterotopic":ti,ab,kw

OR "heterotopic bone":ti,ab,kw

OR "Ossification, Ectopic":ti,ab,kw

OR "Ossification, Pathologic":ti,ab,kw

OR "Pathological Ossification":ti,ab,kw

OR "Pathologic Ossification":ti,ab,kw

OR "Ectopic Ossification":ti,ab,kw

OR (ectopic:ti,ab,kw AND (ossification:ti,ab,kw OR "bone formation":ti,ab,kw OR osteogenesis:ti,ab,kw))

OR [mh "Myositis Ossificans"]

OR "myositis ossificans":ti,ab,kw

OR paraosteoarthropath*:ti,ab,kw

OR (neurogenic:ti,ab,kw AND heterotopic:ti,ab,kw AND ossification:ti,ab,kw)

OR ((HO:ti,ab,kw OR H.O.:ti,ab,kw) AND (ossification:ti,ab,kw OR bone:ti,ab,kw))

OR (calcif*:ti,ab,kw AND (heterotopic:ti,ab,kw OR ectopic:ti,ab,kw OR ossifica*:ti,ab,kw OR ossification:ti,ab,kw OR "bone formation":ti,ab,kw OR "myositis ossificans":ti,ab,kw))

OR (calcin*:ti,ab,kw AND (heterotopic:ti,ab,kw OR ectopic:ti,ab,kw OR ossifica*:ti,ab,kw OR ossification:ti,ab,kw OR "bone formation":ti,ab,kw OR "myositis ossificans":ti,ab,kw))

)

AND

(

[mh Knee]

OR [mh "Knee Joint"]

OR knee:ti,ab,kw

OR knee*:ti,ab,kw

OR tibiofemoral:ti,ab,kw

OR patellofemoral:ti,ab,kw

OR patell*:ti,ab,kw

OR "Joint, Knee":ti,ab,kw

OR "Knee Joints":ti,ab,kw

OR "Superior Tibiofibular Joint":ti,ab,kw

OR "Superior Tibiofibular Joints":ti,ab,kw

OR "Tibiofibular Joint, Superior":ti,ab,kw

OR "Proximal Tibiofibular Joint":ti,ab,kw

OR [mh "Arthroplasty, Replacement, Knee"]

OR (arthroplast*:ti,ab,kw AND knee:ti,ab,kw)

OR (replace*:ti,ab,kw AND knee:ti,ab,kw)

OR TKA:ti,ab,kw

OR TKR:ti,ab,kw

OR UKA:ti,ab,kw

OR UKR:ti,ab,kw

)

**Embase (Elsevier): 1,113 results**

(

'heterotopic ossification'/exp

OR heterotopic ossifica*:ti,ab,kw

OR 'ossification heterotopic':ti,ab,kw

OR 'heterotopic bone':ti,ab,kw

OR 'ossification, ectopic':ti,ab,kw

OR 'ossification, pathologic':ti,ab,kw

OR 'pathological ossification':ti,ab,kw

OR 'pathologic ossification':ti,ab,kw

OR 'ectopic ossification':ti,ab,kw

OR (ectopic AND (ossification OR 'bone formation' OR osteogenesis)):ti,ab,kw

OR 'myositis ossificans'/exp

OR 'myositis ossificans':ti,ab,kw

OR paraosteoarthropath*:ti,ab,kw

OR ((HO OR 'H.O.') AND (ossification OR bone)):ti,ab,kw

OR (calcif* AND (heterotopic OR ectopic OR ossifica* OR ossification OR 'bone formation' OR 'myositis ossificans')):ti,ab,kw

OR (calcin* AND (heterotopic OR ectopic OR ossifica* OR ossification OR 'bone formation' OR 'myositis ossificans')):ti,ab,kw

)

AND

(

'knee'/exp

OR 'knee joint'/exp

OR knee*:ti,ab,kw

OR tibiofemoral:ti,ab,kw

OR patellofemoral:ti,ab,kw

OR patell*:ti,ab,kw

OR 'joint, knee':ti,ab,kw

OR 'knee joints':ti,ab,kw

OR 'superior tibiofibular joint':ti,ab,kw

OR 'superior tibiofibular joints':ti,ab,kw

OR 'tibiofibular joint, superior':ti,ab,kw

OR 'proximal tibiofibular joint':ti,ab,kw

OR 'knee arthroplasty'/exp

OR (arthroplast* AND knee):ti,ab,kw

OR (replace* AND knee):ti,ab,kw

OR TKA:ti,ab,kw

OR TKR:ti,ab,kw

OR UKA:ti,ab,kw

OR UKR:ti,ab,kw

)

**https://scholar.google.de: 139 results**

("heterotopic ossification" OR "myositis ossificans" OR "ectopic ossification" OR "heterotopic bone")

(knee OR "total knee arthroplasty" OR TKA OR TKR OR "knee replacement")

(prophylaxis OR prevention OR prophylactic OR CPM OR radiotherapy OR irradiation OR NSAID OR indomethacin)

**ProQuest Dissertations & Theses Open: 126 results**

(TI,AB("heterotopic ossification" OR ("heterotopic" NEAR/3 ossif*) OR "ectopic ossification" OR "myositis ossificans" OR paraosteoarthropath* OR "heterotopic bone")) AND

(TI,AB(prophylax* OR prevent* OR prophylactic OR preventive OR NSAID* OR indomethacin OR radiotherap* OR irradiat*)) AND

(knee OR "knee arthroplasty" OR "knee replacement" OR "total knee" OR TKA OR TKR)

**https://clinicaltrials.gov: 12 results**

Heterotopic ossification (Condition)

Prophylaxis (other term)

**https://trialsearch.who.int: 7 results**

heterotopic ossification AND prophylaxis
