# Supplementary material for: The prevention of heterotopic ossification around the knee: a scoping review
Source: BMC Musculoskelet Disord. 2026 Aug 1;27:651. doi: 10.1186/s12891-026-10318-w (PMC13428452; doi:10.1186/s12891-026-10318-w)
Supplement: Supplementary file 2 — Supplementary Material 2. [file 12891_2026_10318_MOESM2_ESM.docx]

**Supplement S2:** Tabular inclusion- and exclusion criteria by PCC domain

| PCC domain | Inclusion criteria | Exclusion criteria |
| --- | --- | --- |
| Population | Studies enrolling ≥75% adult participants. Studies including other HO localizations were eligible if knee-specific data were reported separately or if ≥75% of the relevant study population concerned HO around the knee. | Studies with <75% adult participants will be excluded. |
| Concept | Any predefined, modifiable intervention or treatment strategy in relation to the development or recurrence of HO around the knee, irrespective of modality. This includes, but is not limited to, NSAIDs, RT, physiotherapy, and specific surgical techniques employed to reduce HO risk at the knee. | No restrictions by intervention type, dose/schedule, timing, or combination strategies.  Studies that did not directly evaluate a predefined, modifiable intervention or treatment strategy in relation to HO around the knee. |
| Context | No geographical restrictions; evidence from any country, region, healthcare system, or cultural context will be included. | None. |

The eligibility criteria follow the Population–Concept–Context (PCC) framework recommended for scoping reviews. Inclusion is intentionally broad to maximize sensitivity and capture the full range of evidence on prophylactic strategies to prevent HO around the knee.

Further abbreviations: HO, heterotopic ossification; NSAIDs, non-steroidal anti-inflammatory drugs; RT, radiotherapy
