# Supplementary material for: The prevention of heterotopic ossification around the knee: a scoping review
Source: BMC Musculoskelet Disord. 2026 Aug 1;27:651. doi: 10.1186/s12891-026-10318-w (PMC13428452; doi:10.1186/s12891-026-10318-w)
Supplement: Supplementary file 3 — Supplementary Material 3. [file 12891_2026_10318_MOESM3_ESM.docx]

**Supplement S3:** Articles that could not be retrieved and articles excluded after full-text screening

**Articles that could not be retrieved:**

| **Author, Year** | **Type and title of the article** |
| --- | --- |
| Chao, 2007 | Review on the treatment of HO |
| Denormandie, 2012 | Review on the surgical treatment of neurological HO |
| Freedman, 1996 | Case report on the resection of HO around the knee |
| Gacon, 1975 | Review type article on the surgical management of HO in general |
| Gacon, 1978 | Report on the resection of neurogenic HO in 70 cases, including twelve knees |
| Garcia, 1994 | Case report of two cases of neurogenic HO |
| Genta, 1975 | Review on the surgical treatment of neurological HO |
| Guillaumat, 1976 | Case series reporting the results of surgery for neurogenic HO |
| Hartmann, 1953 | Article on HO of the patellar ligament |
| Holstein, 1964 | No further information available, as no abstract could be located. |
| Howell, 2011 | Case report on HO of the patellar tendon after trauma |
| Jonasch, 1957 | Article on HO on the internal side of the knee |
| Meoli, 1994 | Case report on ossifying macroenthesopathy of the patellar tendon |
| Pizon, 1955 | No further information available, as no abstract could be located |
| Rooney, 1996 | Case report and review on the arthroscopic excision of intra-articular ossification |
| Schenck, 2006 | Technical guide focusing on the surgical reconstruction of the dislocated knee |
| Tigani, 2004 | Retrospective study with the purpose of ascertaining the incidence of heterotopic ossifications after total knee arthroplasty |
| Zeng, 2020 | Article on the prevention and treatment of HO in general |

**Articles excluded after full-text screening:**

| **Author, Year** | **Reason for exclusion and additional comments if applicable** |
| --- | --- |
| Abdlslam, 2003 | No intervention was directly evaluated in relation to the development or recurrence of knee HO. |
| Abdul-Jabar, 2013 | No intervention was directly evaluated in relation to the development or recurrence of knee HO. |
| Achour, 2016 | No intervention was directly evaluated in relation to the development or recurrence of knee HO. |
| Adiguzel, 2015 | No intervention was directly evaluated in relation to the development or recurrence of knee HO. |
| Agarwal, 2015 | Preclinical analysis only |
| Ahmed, 2013 | No sufficient knee-specific information, one potentially eligible patient, but only fracture localisation (femur shaft) reported, not the location of HO |
| Ahrengart, 1988 | Preclinical analysis only |
| Aikebaier, 2022 | No intervention was directly evaluated in relation to the development or recurrence of knee HO. |
| Aldhilan, 2024 (1) | No intervention was directly evaluated in relation to the development or recurrence of knee HO. |
| Aldhilan, 2024 (2) | No intervention was directly evaluated in relation to the development or recurrence of knee HO. |
| Ali, 2019 | No intervention was directly evaluated in relation to the development or recurrence of knee HO. |
| Aljasim,2023 | No intervention was directly evaluated in relation to the development or recurrence of knee HO. |
| Alkhuzaei, 2021 | No intervention was directly evaluated in relation to the development or recurrence of knee HO. |
| Altindag, 2010 | No intervention was directly evaluated in relation to the development or recurrence of knee HO; only pharmacological therapy of established HO |
| Amstalden, 2010 | Abstract only, No intervention was directly evaluated in relation to the development or recurrence of knee HO. |
| Arabi, 2016 | No intervention was directly evaluated in relation to the development or recurrence of knee HO. |
| Argyropoulou, 2006 | No intervention was directly evaluated in relation to the development or recurrence of knee HO. |
| Atamaz, 2006 | No intervention was directly evaluated in relation to the development or recurrence of knee HO. |
| Aubut, 2011 | No separate information regarding HO around the knee, besides mentioning studies that were already assessed |
| Austin 1995 | No intervention was directly evaluated in relation to the development or recurrence of knee HO. |
| Axelrod 2011 | No intervention was directly evaluated in relation to the development or recurrence of knee HO; only pharmacological therapy of established HO |
| Ayhan, 2026 | No HO prophylaxis, analysis on risk factors for HO around the knee, not included in primary synthesis, but key data on risk factors extracted |
| Baier, 2012 | RT for the prevention of arthrofibrosis, not HO |
| Barei, 2004 | No intervention was directly evaluated in relation to the development or recurrence of knee HO. |
| Ben Hamida, 2004 | No intervention was directly evaluated in relation to the development or recurrence of knee HO; only therapy of established HO |
| Ben-Youssef, 2015 | No intervention was directly evaluated in relation to the development or recurrence of knee HO. |
| Berndt, 2008 | No intervention was directly evaluated in relation to the development or recurrence of knee HO. |
| Bhavani, 2024 | No intervention was directly evaluated in relation to the development or recurrence of knee HO. |
| Biosca Florensa, 1956 | No intervention was directly evaluated in relation to the development or recurrence of knee HO. |
| Biotti, 2009 | Insufficient details to affirm when pamidronate was applied and if it was used for HO prophylaxis or treatment of established HO |
| Bodley, 1997 | No intervention was directly evaluated in relation to the development or recurrence of knee HO. |
| Bong, 2004 | No intervention was directly evaluated in relation to the development or recurrence of knee HO. |
| Booth, 1979 | No intervention was directly evaluated in relation to the development or recurrence of knee HO. |
| Botzoris, 2009 | No intervention was directly evaluated in relation to the development or recurrence of knee HO; only pharmacological therapy of established HO |
| Boukhris, 2014 | No intervention was directly evaluated in relation to the development or recurrence of knee HO. |
| Boulezaz, 2016 | No intervention was directly evaluated in relation to the development or recurrence of knee HO. |
| Brady, 2018 | No separate information for the knee |
| Brance, 2022 | No intervention was directly evaluated in relation to the development or recurrence of knee HO. |
| Brander, 2006 | No separate information for the knee |
| Brien, 1999 | No intervention was directly evaluated in relation to the development or recurrence of knee HO. |
| Bryson, 2009 | No intervention was directly evaluated in relation to the development or recurrence of knee HO. |
| Carpentier, 2019 | No intervention was directly evaluated in relation to the development or recurrence of knee HO. |
| Carulli, 2012 | No HO prophylaxis for the knee was reported |
| Carvalho, 2014 | No intervention was directly evaluated in relation to the development or recurrence of knee HO. |
| Chalidis, 2007 | No separate information for the knee |
| Chan, 2005 | No separate information for the knee |
| Chan, 2014 | No intervention was directly evaluated in relation to the development or recurrence of knee HO. |
| Chen, 2011 | No intervention was directly evaluated in relation to the development or recurrence of knee HO. |
| Choumad, 2026 | No intervention was directly evaluated in relation to the development or recurrence of knee HO. |
| Christakou, 2012 | No intervention was directly evaluated in relation to the development or recurrence of knee HO. |
| Ciriello, 2012 | No intervention was directly evaluated in relation to the development or recurrence of knee HO. |
| Citak, 2009 | No HO prophylaxis used |
| Clements, 1993 | No intervention was directly evaluated in relation to the development or recurrence of knee HO. |
| Coelho, 2009 | No separate information for the knee |
| Cohen, 2018 | No separate information for HO around the knee, only stiffness in general |
| Cook, 2005 | No intervention was directly evaluated in relation to the development or recurrence of knee HO; only pharmacological therapy of established HO |
| Cormick, 1992 | No intervention was directly evaluated in relation to the development or recurrence of knee HO. |
| Cunha, 2016 | No intervention was directly evaluated in relation to the development or recurrence of knee HO. |
| Cyteval, 2016 | No intervention was directly evaluated in relation to the development or recurrence of knee HO. |
| Damrow, 2017 | Non-adult participant |
| Daugherty, 2010 | Abstract only, reports on the same cohort as Daugherty, 2013, which is included |
| Davies, 2011 | Non-adult participant |
| De Brier, 2015 | No separate information for HO around the knee |
| Debbi, 2021 | No intervention was directly evaluated in relation to the development or recurrence of knee HO. |
| Devnani, 2008 | No intervention was directly evaluated in relation to the development or recurrence of knee HO. |
| Dhifallah, 2025 | No intervention was directly evaluated in relation to the development or recurrence of knee HO. |
| Dizdar, 2013 | No separate data for the knee, No intervention was directly evaluated in relation to the development or recurrence of knee HO. |
| Dodds, 2014 | No intervention was directly evaluated in relation to the development or recurrence of knee HO. |
| Engh, 1999 | No intervention was directly evaluated in relation to the development or recurrence of knee HO. |
| Enjalbert, 1995 | No intervention was directly evaluated in relation to the development or recurrence of knee HO; only pharmacological therapy of established HO |
| Erdogan, 2004 | No intervention was directly evaluated in relation to the development or recurrence of knee HO. |
| Erler, 2009 | No intervention was directly evaluated in relation to the development or recurrence of knee HO. |
| Exner, 1983 | Non-adult participant |
| Facchin, 2025 | No separate information for HO around the knee |
| Fachri, 2024 | No intervention was directly evaluated in relation to the development or recurrence of knee HO. |
| Falsetti, 2011 | No intervention was directly evaluated in relation to the development or recurrence of knee HO; only pharmacological therapy of established HO |
| Flin, 2002 | No intervention was directly evaluated in relation to the development or recurrence of knee HO. |
| Forsberg, 2009 | No intervention was directly evaluated in relation to the development or recurrence of knee HO. |
| Furia, 1995 | No intervention was directly evaluated in relation to the development or recurrence of knee HO. |
| Furman, 1970 | No intervention was directly evaluated in relation to the development or recurrence of knee HO. |
| Gagnaire, 1994 | No intervention was directly evaluated in relation to the development or recurrence of knee HO. |
| Garland, 1991 | No separate information for HO prophylaxis around the knee |
| Garland, 1980 | No intervention was directly evaluated in relation to the development or recurrence of knee HO. |
| Genêt, 2012 | No separate information for HO prophylaxis around the knee |
| Genêt, 2011 | No intervention was directly evaluated in relation to the development or recurrence of knee HO. |
| Gerstenberg, 1925 | No intervention was directly evaluated in relation to the development or recurrence of knee HO. |
| Gkiatas, 2021 | No HO prophylaxis, analysis on risk factors for HO around the knee, not included in primary synthesis, but key data on risk factors extracted |
| Gockel, 1961 | No intervention was directly evaluated in relation to the development or recurrence of knee HO. |
| Goodman, 1997 | No intervention was directly evaluated in relation to the development or recurrence of knee HO. |
| Gorgey, 2016 | No intervention was directly evaluated in relation to the development or recurrence of knee HO. |
| Gosselin, 1993 | No intervention was directly evaluated in relation to the development or recurrence of knee HO. |
| Goyal, 2015 | No intervention was directly evaluated in relation to the development or recurrence of knee HO. |
| Graci, 1980 | No intervention was directly evaluated in relation to the development or recurrence of knee HO. |
| Grossfeld, 1995 | No intervention was directly evaluated in relation to the development or recurrence of knee HO. |
| Guo, 2019 | No intervention was directly evaluated in relation to the development or recurrence of knee HO; only pharmacological therapy of established HO |
| Gürcay, 2013 | No intervention was directly evaluated in relation to the development or recurrence of knee HO. |
| Haasper, 2007 | No intervention was directly evaluated in relation to the development or recurrence of knee HO. |
| Hack, 2015 | No intervention was directly evaluated in relation to the development or recurrence of knee HO. |
| Hafer, 1961 | No intervention was directly evaluated in relation to the development or recurrence of knee HO. |
| Hamida, 2004 | No intervention was directly evaluated in relation to the development or recurrence of knee HO; only pharmacological therapy of established HO |
| Hardy, 2005 | No intervention was directly evaluated in relation to the development or recurrence of knee HO. |
| Harris, 2020 | No intervention was directly evaluated in relation to the development or recurrence of knee HO; only treatment of established HO |
| Harwin, 1995 | No intervention was directly evaluated in relation to the development or recurrence of knee HO. |
| Hasegawa, 2002 | No intervention was directly evaluated in relation to the development or recurrence of knee HO. |
| Hemmann, 2022 | HO after proximal hamstring rupture close to the hip, not the knee |
| Henderson, 2005 | No intervention was directly evaluated in relation to the development or recurrence of knee HO. |
| Hernandez, 1978 | No separate information for HO around the knee, No intervention was directly evaluated in relation to the development or recurrence of knee HO. |
| Heuck, 1974 | No intervention was directly evaluated in relation to the development or recurrence of knee HO. |
| Hewitt, 2002 | No separate information for HO around the knee, No intervention was directly evaluated in relation to the development or recurrence of knee HO. |
| Horino, 2023 | No intervention was directly evaluated in relation to the development or recurrence of knee HO. |
| Horne, 1999 | No intervention was directly evaluated in relation to the development or recurrence of knee HO. |
| Hosur Ravishankar, 2020 | No intervention was directly evaluated in relation to the development or recurrence of knee HO. |
| Hua, 2016 | No intervention was directly evaluated in relation to the development or recurrence of knee HO. |
| Hunt, 2006 | No separate information for HO around the knee |
| Hussain, 2017 | No separate information on HO around the knee |
| Iqbal, 2021 | No intervention was directly evaluated in relation to the development or recurrence of knee HO; only therapy of established HO |
| Jamshidi, 2021 | No intervention was directly evaluated in relation to the development or recurrence of knee HO. |
| Jang, 2017 | No HO only calcification, no prophylaxis used |
| Jerabek, 2009 | No intervention was directly evaluated in relation to the development or recurrence of knee HO. |
| Jonasch, 1963 | No intervention was directly evaluated in relation to the development or recurrence of knee HO. |
| Jones, 2024 | No HO prophylaxis, analysis on risk factors for HO around the knee, not included in primary synthesis, but key data on risk factors extracted |
| Joon Cheol, 1999 | No intervention was directly evaluated in relation to the development or recurrence of knee HO. |
| Joseph, 2014 | No intervention was directly evaluated in relation to the development or recurrence of knee HO; only therapy of established HO |
| Jung, 2007 | No intervention was directly evaluated in relation to the development or recurrence of knee HO. |
| Kalbi, 2025 | No separate information for HO around the knee |
| Kambhampati, 2021 | Non-adult participant |
| Kandaz, 2019 | Only information for HO prevention in non-knee joints |
| Kara, 2016 | No intervention was directly evaluated in relation to the development or recurrence of knee HO; only therapy of established HO |
| Karakatsanis, 2025 | No intervention was directly evaluated in relation to the development or recurrence of knee HO. |
| Karimi, 2023 | No intervention was directly evaluated in relation to the development or recurrence of knee HO. |
| Keel, 1999 | No intervention was directly evaluated in relation to the development or recurrence of knee HO. |
| Kelly, 1987 | No intervention was directly evaluated in relation to the development or recurrence of knee HO. |
| Kesikburun, 2011 | No intervention was directly evaluated in relation to the development or recurrence of knee HO. |
| Khan, 2022 | No intervention was directly evaluated in relation to the development or recurrence of knee HO. |
| Kim, 2016 | No intervention was directly evaluated in relation to the development or recurrence of knee HO. |
| Kleipool, 1994 | No intervention was directly evaluated in relation to the development or recurrence of knee HO. |
| Kocaağa, 2007 | No intervention was directly evaluated in relation to the development or recurrence of knee HO. |
| Kossoff, 1979 | No intervention was directly evaluated in relation to the development or recurrence of knee HO. |
| Kowalczyk, 2019 | No intervention was directly evaluated in relation to the development or recurrence of knee HO. |
| Koyuncu, 2016 | No intervention was directly evaluated in relation to the development or recurrence of knee HO; non-knee joint |
| Krishna, 2023 | No intervention was directly evaluated in relation to the development or recurrence of knee HO. |
| Kujawski, 2012 | No intervention was directly evaluated in relation to the development or recurrence of knee HO. |
| Kumar, 2021 | No intervention was directly evaluated in relation to the development or recurrence of knee HO. |
| Kun, 1992 | No intervention was directly evaluated in relation to the development or recurrence of knee HO. |
| Kwai, 2018 | No intervention was directly evaluated in relation to the development or recurrence of knee HO. |
| Laczay, 1973 | No intervention was directly evaluated in relation to the development or recurrence of knee HO. |
| Laczay, 1971 | Non-adult participant |
| Lagier 1991 | No intervention was directly evaluated in relation to the development or recurrence of knee HO. |
| Lane, 2002 | No intervention was directly evaluated in relation to the development or recurrence of knee HO. |
| Larson, 2002 | Non-knee joint |
| Lawand, 2023 | Only mentions the already included study by Kent et al. regarding HO around the knee |
| Lee, 2020 | No intervention was directly evaluated in relation to the development or recurrence of knee HO; only therapy of established HO |
| Lespasio, 2020 | No separate information for HO around the knee |
| Leung, 2010 | Non-adult participant, No intervention was directly evaluated in relation to the development or recurrence of knee HO. |
| Levy, 2010 | No intervention was directly evaluated in relation to the development or recurrence of knee HO. |
| Li, 2021 | No intervention was directly evaluated in relation to the development or recurrence of knee HO. |
| Li, 2018 | No intervention was directly evaluated in relation to the development or recurrence of knee HO. |
| Liechti, 2023 | No intervention was directly evaluated in relation to the development or recurrence of knee HO. |
| Lin, 2016 | Non-adult participant, No intervention was directly evaluated in relation to the development or recurrence of knee HO. |
| Lin, 2011 | No intervention was directly evaluated in relation to the development or recurrence of knee HO. |
| Linan, 2001 | No intervention was directly evaluated in relation to the development or recurrence of knee HO; only therapy of established HO |
| Liu, 2025 | No intervention was directly evaluated in relation to the development or recurrence of knee HO. |
| Lonner, 1995 | No intervention was directly evaluated in relation to the development or recurrence of knee HO. |
| Lovelock, 1984 | No intervention was directly evaluated in relation to the development or recurrence of knee HO. |
| Macurak, 1980 | No intervention was directly evaluated in relation to the development or recurrence of knee HO. |
| Malca, 2018 | No intervention was directly evaluated in relation to the development or recurrence of knee HO. |
| Martin, 2021 | No intervention was directly evaluated in relation to the development or recurrence of knee HO. |
| McClelland, 1986 | No intervention was directly evaluated in relation to the development or recurrence of knee HO. |
| Mendelson, 1975 | No separate information for HO around the knee |
| Meunier, 2009 | Celecoxib used as an analgetic, HO rates not reported |
| Minaire, 1980 | No separate information for HO around the knee |
| Mishra, 2010 | Abstract only, reports on the same cohort as the included full-text Mishra, 2011 |
| Mody, 2021 | No intervention was directly evaluated in relation to the development or recurrence of knee HO. |
| Money, 1972 | No intervention was directly evaluated in relation to the development or recurrence of knee HO. |
| Mundy, 2003 | No intervention was directly evaluated in relation to the development or recurrence of knee HO. |
| Mungalpara, 2023 | No intervention was directly evaluated in relation to the development or recurrence of knee HO. |
| Muñoz-Maldonado, 2021 | No intervention was directly evaluated in relation to the development or recurrence of knee HO. for the knee, only therapy of established HO |
| Nadar, 2023 | No intervention was directly evaluated in relation to the development or recurrence of knee HO. |
| Naftchi, 1979 | No intervention was directly evaluated in relation to the development or recurrence of knee HO; only therapy of established HO |
| Nait Khachat, 2014 | No intervention was directly evaluated in relation to the development or recurrence of knee HO. |
| Nakajima, 2013 | No intervention was directly evaluated in relation to the development or recurrence of knee HO. |
| Ngo, 2010 | No intervention was directly evaluated in relation to the development or recurrence of knee HO. |
| Nicholas, 1984 | No intervention was directly evaluated in relation to the development or recurrence of knee HO. |
| Nota, 2014 | No intervention was directly evaluated in relation to the development or recurrence of knee HO. |
| Ogawa, 2025 | No intervention was directly evaluated in relation to the development or recurrence of knee HO; only therapy of established HO |
| Osnach, 2023 | No intervention was directly evaluated in relation to the development or recurrence of knee HO. |
| Oubadi, 2019 | No intervention was directly evaluated in relation to the development or recurrence of knee HO. |
| Özbek, 2023 | No intervention was directly evaluated in relation to the development or recurrence of knee HO. |
| Ozen, 2020 | No intervention was directly evaluated in relation to the development or recurrence of knee HO. |
| Ozkocak, 2018 | Non-adult participant, No intervention was directly evaluated in relation to the development or recurrence of knee HO. |
| Patton, 2000 | No intervention was directly evaluated in relation to the development or recurrence of knee HO. |
| Pellegrini, 1994 | No separate information for HO around the knee |
| Peylan, 1987 | No intervention was directly evaluated in relation to the development or recurrence of knee HO. |
| Pierreux, 2025 | No intervention was directly evaluated in relation to the development or recurrence of knee HO. |
| Popovic, 2014 | Only mentions the already included studies by Mishra and Daugherty et al. regarding HO around the knee |
| Rajeswaran, 2011 | No intervention was directly evaluated in relation to the development or recurrence of knee HO. |
| Randall, 2024 | No intervention was directly evaluated in relation to the development or recurrence of knee HO. |
| Rawat, 2019 | No intervention was directly evaluated in relation to the development or recurrence of knee HO; no separate information for HO around the knee |
| Rehman, 2019 | No intervention was directly evaluated in relation to the development or recurrence of knee HO. |
| Reynolds, 2021 | No intervention was directly evaluated in relation to the development or recurrence of knee HO; only therapy of established HO |
| Reznik, 2017 | No intervention was directly evaluated in relation to the development or recurrence of knee HO; only therapy of established HO (two publications ecluded, reporting on the same cohort) |
| Richards, 1997 | No intervention was directly evaluated in relation to the development or recurrence of knee HO. |
| Richardson, 2012 | No intervention was directly evaluated in relation to the development or recurrence of knee HO. |
| Ries, 2000 | No intervention was directly evaluated in relation to the development or recurrence of knee HO. |
| Roth, 2014 | No intervention was directly evaluated in relation to the development or recurrence of knee HO. |
| Sacher, 2017 | No intervention was directly evaluated in relation to the development or recurrence of knee HO. |
| Sahin, 2010 | No separate information for HO around the knee |
| Saito, 2004 | No intervention was directly evaluated in relation to the development or recurrence of knee HO; only therapy of established HO |
| Samuel, 2024 | No separate information for HO around the knee |
| Sautter-Bihl, 2001 | Only 3 patients with HO around the knee, in these patients only therapy of established HO |
| Sazbon, 1981 | No intervention was directly evaluated in relation to the development or recurrence of knee HO. |
| Scarcella, 2017 | No intervention was directly evaluated in relation to the development or recurrence of knee HO. |
| Schiavone Panni, 2009 | No intervention was directly evaluated in relation to the development or recurrence of knee HO. |
| Seil, 2011 | No intervention was directly evaluated in relation to the development or recurrence of knee HO. |
| Sharma, 2014 | No intervention was directly evaluated in relation to the development or recurrence of knee HO; only therapy of established HO |
| Sherman, 2022 | No intervention was directly evaluated in relation to the development or recurrence of knee HO. for the knee |
| Shinault, 2007 | No intervention was directly evaluated in relation to the development or recurrence of knee HO. |
| Siqueira, 2012 | No intervention was directly evaluated in relation to the development or recurrence of knee HO. |
| Şirin, 2024 | No intervention was directly evaluated in relation to the development or recurrence of knee HO. |
| Skorochod, 2022 | No separate information for HO around the knee |
| Smith, 2020 | No intervention was directly evaluated in relation to the development or recurrence of knee HO; RT was used to prevent recurrent arthrofibrosis |
| Sneha, 2025 | No intervention was directly evaluated in relation to the development or recurrence of knee HO. for the knee |
| Somani, 2024 | No intervention was directly evaluated in relation to the development or recurrence of knee HO. |
| Stover, 1975 | No intervention was directly evaluated in relation to the development or recurrence of knee HO; only therapy of established HO |
| Sung, 2023 | No intervention was directly evaluated in relation to the development or recurrence of knee HO. |
| Supreeth, 2019 | Non-adult participant |
| Tabert, 2013 | No intervention was directly evaluated in relation to the development or recurrence of knee HO. |
| Taly, 2001 | No separate information for HO around the knee |
| Tan, 2017 | No intervention was directly evaluated in relation to the development or recurrence of knee HO. |
| Tang, 2015 | No intervention was directly evaluated in relation to the development or recurrence of knee HO. |
| Tornetta 3rd, 1992 | No intervention was directly evaluated in relation to the development or recurrence of knee HO. |
| Toyoda, 2003 | No intervention was directly evaluated in relation to the development or recurrence of knee HO. |
| Tsugeno, 2024 | No intervention was directly evaluated in relation to the development or recurrence of knee HO; only therapy of established HO |
| Vaishya, 2016 | No intervention was directly evaluated in relation to the development or recurrence of knee HO; only therapy of established HO |
| Vanden Berge, 2022 | No intervention was directly evaluated in relation to the development or recurrence of knee HO. |
| Vun, 2015 | No intervention was directly evaluated in relation to the development or recurrence of knee HO. |
| Wheeler, 2014 | No intervention was directly evaluated in relation to the development or recurrence of knee HO. |
| Wong, 2020 | No separate information for HO around the knee |
| Yamagami, 2016 | No intervention was directly evaluated in relation to the development or recurrence of knee HO; only therapy of established HO |
| Yamamoto, 2018 | No intervention was directly evaluated in relation to the development or recurrence of knee HO. |
| Yuan, 2021 | No separate information for HO around the knee |
| Zamora, 2022 | No intervention was directly evaluated in relation to the development or recurrence of knee HO. |
| Zhang, D.L., 2024 | No intervention was directly evaluated in relation to the development or recurrence of knee HO. |
| Zhang, F., 2015 | No separate information for HO around the knee |
| Zhang, J., 2020 | No intervention was directly evaluated in relation to the development or recurrence of knee HO. |
| Zhao, E.Z., 2022 | No intervention was directly evaluated in relation to the development or recurrence of knee HO. |
| Zhao, Q.,2022 | No intervention was directly evaluated in relation to the development or recurrence of knee HO. |
| Zhou, 2022 | No separate information for the prophylaxis of HO around the knee |
| Zietek, 2015 | No intervention was directly evaluated in relation to the development or recurrence of knee HO. |

Abbreviations: HO, heterotopic ossification; RT, radiotherapy.
