# Supplementary material for: The prevention of heterotopic ossification around the knee: a scoping review
Source: BMC Musculoskelet Disord. 2026 Aug 1;27:651. doi: 10.1186/s12891-026-10318-w (PMC13428452; doi:10.1186/s12891-026-10318-w)
Supplement: Supplementary file 4 — Supplementary Material 4. [file 12891_2026_10318_MOESM4_ESM.docx]

**Supplement S4:** Expert opinions and recommendations regarding prophylaxis of HO around the knee.

| **First author, year** | **Country** | **Source type** | **Scenario** | **Recommended strategy** | **Recommended modality of HO prophylaxis** | **Details on prophylactic strategy** | **Potential complications mentioned** | **Evidence & strength/ grade** | **Knee specificity** | **Further recommendations and comments** |
| --- | --- | --- | --- | --- | --- | --- | --- | --- | --- | --- |
| Board, 2007(1) | UK (England) | Narrative review | Lower limb arthroplasty | No indication for prophylaxis in routine arthroplasty:  Primary prophylaxis may be used in high-risk patients  Recurrence prophylaxis should be used after removal of HO | RT or NSAID may be used for HO prophylaxis  RT: marginally more effective  NSAID: cheaper, easier to deliver  RT and NSAID may be combined in recurrence prophylaxis | NSAIDs: Indomethacin: 25 mg TID for 5-6 weeks post-op  RT: single fraction 7.0-8.0 Gy, pre-op: <4 h before surgery, or post-op: within 72 h  RT+NSAID: Indomethacin + RT delivered similarly to monotherapy | NSAID: increased risk of major bleeding  RT: potential for inducing malignancies, testicular damage, impaired healing of fractures | Evidence mainly from hip trials  No formal grading/strength provided | Not knee specific, only lower limb arthroplasty in general | Excision of HO should be postponed until maturation |
| Garland, 2000(2) | USA | Letter to the editor | Neurogenic HO around the knee | Recurrence prophylaxis may be used | Indomethacin and RT may be used to prevent recurrence  CPM may assist in maintaining joint motion but does not prevent HO recurrence | NR | NR | Evidence mainly from hip trials  No formal grading/strength provided | Yes | NR |
| Gkiatas, 2021(3) | Greece | Systematic review and meta-analysis | TKA | Primary HO prophylaxis should be considered in high-risk patients | RT or NSAID may be used for HO prophylaxis  Superior effect when RT+NSAID are used | NSAID: dose and schedule  RT: doses: 4,0-8,0 Gy, schedule: NR | NSAID: NR  RT: non-union, wound healing complications, oligospermia, induced malignancies | Evidence mainly from hip trials  No formal grading/strength provided | Yes, specific for TKA | Aspirin used for the prophylaxis of VTE is effective in the prevention of HO |
| Guerin, 2025(4) | USA | Narrative review | Adhesions or HO after MCL reconstruction | HO prophylaxis should be used in all patients | Indomethacin daily for HO prophylaxis  Use of CPM to maintain full ROM | Indomethacin: dose and schedule NR | NR | No evidence cited for HO prophylaxis  No formal grading/strength provided | Yes, specific for HO after MCL reconstruction | NR |
| Iorio, 2002(5) | USA | Narrative review | THA and TKA | No indication for prophylaxis in routine THA or TKA  Recurrence prophylaxis is necessary | NSAIDs or RT are preferred | NSAID: Indomethacin: 25 mg TID for 7-14 days post-op  RT: single fraction 8.0 Gy, pre-op:  <6 h before surgery or single fraction 6.0-8.0 Gy  <5 d post-op | NSAID: increased bleeding risk, adverse effect on bone ingrowth  RT: potential to induce malignancies, non-union adverse effects on bone ingrowth | Evidence mainly from hip trials  No formal grading/strength provided | Not knee specific, combined recommendations for hip and knee arthroplasty | Recommendation against the use of diphosphates, since mineralization is only delayed, but HO formation is not decreased |
| James, 2021(6) | USA | Narrative review | PCL reconstruction | No prophylaxis recommended as RT and NSAIDs have not been investigated in this context | None | NR | NR | No formal grading/strength provided | Yes, specific for PCL reconstruction | Resection of HO can be performed after maturation of HO |
| Lubowitz, 2006(7) | USA | Narrative review | Medial and lateral sided injuries of the knee | Primary prophylaxis can be considered in high-risk patients  Recurrence prophylaxis is generally required | Indomethacin or RT may be used in primary prophylaxis  RT should be used as recurrence prophylaxis | Indomethacin: dose and schedule NR RT: dose and schedule NR | NR | Knee-specific literature (tom, 2003) cited as evidence  No formal grading/strength provided | Yes, medial and lateral sided injuries of the knee | High-risk patients defined as: open-knee dislocation, prior history of HO, and use of debridement and irrigation during trauma management |
| Manrique, 2015(8) | USA | Narrative review | TKA | Primary prophylaxis should be administered in high-risk patients  Recurrence prophylaxis should be administered | NSAIDs or RT or selective COX2-inhibitors may be used | NSAID: Indomethacin: 25 mg TID for 5-6 weeks post-op  RT: single fraction 7.0 Gy, pre-op: <4 h before surgery, or post-op: within 72 h  Selective COX2-inhibitors: Celecoxib, dose and schedule NR | NSAID: increased risk of postoperative bleeding, gastrointestinal issues, renal impairment, and bone nonunion | Evidence mainly from hip trials  No formal grading/strength provided | Yes, specific for TKA | High-risk patients defined as: history of HO requiring surgical excision |
| Nelson, 2005(9) | USA | Narrative review | TKA | Recurrence prophylaxis may be administered | Indomethacin or RT | Indomethacin: dose and schedule NR RT: dose and schedule NR | NR | Knee-specific literature cited as evidence  No formal grading/strength provided | Yes, specific for TKA | NR |
| Rader, 1997(10) | Germany | Author opinion in an obser-vational study | TKA | Primary prophylaxis should be considered in high-risk patients | NR | NR | NR | No evidence cited for HO prophylaxis  No formal grading/strength provided | Yes, specific for TKA | High-risk patients defined as: marked hypertrophic arthrosis or marked periosteal damage to the anterior distal femur |
| Rodriguez, 2022(11) | USA | Narrative review | Medial and lateral knee surgery | No clear recommend-dation on the use of dedicated HO prophylaxis | NSAID or RT | NR | NR | Knee-specific literature cited as evidence  No formal grading/strength provided | Yes, specific for medial and lateral knee surgery | Surgical resection may be required, when knee motion is limited |
| Rodríguez-Merchán, 2021(12) | Spain | Narrative review | Multiligament injuries of the knee joint | Primary prophylaxis can be administered in high-risk patients  Recurrence prophylaxis should be administered | Indomethacin | NR | NR | No evidence cited for HO prophylaxis  No formal grading/strength provided | Yes, specific for MLKI | RT not recommended, due to a lack of evidence in patients with MLKI |
| Stannard, 2002(13) | USA | Author opinion in an obser-vational study | Knee dislocation | Primary prophylaxis should be administered in high-risk patients  Recurrence prophylaxis should be administered | Indomethacin or RT; RT only in recurrence prophylaxis or patients with open knee dislocation | Indomethacin: dose and schedule NR RT: dose and schedule NR | NR | No evidence cited for HO prophylaxis  No formal grading/strength provided | Yes, specific for knee dislocation | High-risk patients defined as: open-knee dislocation, prior history of HO, and use of debridement and irrigation during trauma management |
| Sterner, 2005(14) | Germany | Author opinion in an obser-vational study | Primary TKA | Primary prophylaxis is useful in patients with risk factors for HO | NSAID or RT and additional surgical measures | NSAID: dose and schedule similar to THA RT: dose and schedule similar to THA  Surgical measures: excessive rinsing with water to remove bone particles, avoidance of periosteal stripping of the anterior femur | NR | No evidence cited for HO prophylaxis  No formal grading/strength provided | Yes, specific for primary TKA | Risk factors for HO: ankylosing spondylitis, Paget’s disease, ipsilateral or contralateral pre-existing HO, hypertrophic osteoarthritis, other operations on the knee, posttraumatic arthritis, diffuse idiopathic hyperostosis |
| Tom, 2003(15) | USA | Narrative review | Multiligament injuries of the knee joint | Primary prophylaxis should be considered in high-risk patients  Recurrence prophylaxis should be used | Indomethacin or RT may be used in primary prophylaxis  RT should be used as recurrence prophylaxis | Indomethacin: dose and schedule NR RT: dose and schedule NR | NR | Knee-specific literature (Stannard, 2002) cited as evidence  No formal grading/strength provided | Yes, specific for MLKI | NR |
| Wharton, 2026(16) | USA | Narrative review | Knee dislocation | Primary prophylaxis is advisable | NSAID | NR | NR | No evidence cited for HO prophylaxis  No formal grading/strength provided | Yes, specific for knee dislocation | NR |
| Whelan, 2014(17) | Canada | Narrative review | Knee dislocation | Primary prophylaxis may be appropriate in patients receiving PCL reconstruction | NSAID or RT | NR | NR | No evidence cited for HO prophylaxis  No formal grading/strength provided | Yes, specific for knee dislocation | Prospective studies are needed before a firm recommendation on HO prophylaxis is possible |
| Zeckey, 2011(18) | Germany | Narrative review | Implant surgery | Prophylaxis is not recommended as HO following TKA is believed to be self-limiting. | None | NR | NR | Knee-specific literature cited as evidence  No formal grading/strength provided | Yes, recommendations specific for TKA | HO around the knee is believed to be self-limiting and requires neither prophylaxis nor therapy |

Recommendations, evidence grading, and comments are reproduced as stated in the original sources. Knee specificity indicates whether the recommendation explicitly addresses the knee joint.

Abbreviations: COX2, cyclooxygenase-2; CPM, continuous passive motion; HO, heterotopic ossification; MCL, medial collateral ligament; MLKI, multi-ligament knee injury; NR, not reported; NSAID, non-steroidal anti-inflammatory drug; PCL, posterior cruciate ligament; ROM, range of motion; RT, radiotherapy; THA, total hip arthroplasty; TID, three times daily; TKA, total knee arthroplasty; UK, United Kingdom; USA, United States of America; VTE, venous thromboembolism.

**References**

1. Board TN, Karva A, Board RE, Gambhir AK, Porter ML. The prophylaxis and treatment of heterotopic ossification following lower limb arthroplasty. J Bone Joint Surg Br. 2007;89(4):434-40.

2. Garland DE. Periarticular ossification. J Bone Joint Surg Am. 2000;82(8):1206-7.

3. Gkiatas I, Xiang W, Karasavvidis T, Windsor EN, Malahias MA, Tarity TD, et al. Relatively Low Rate of Heterotopic Ossification Following Primary Total Knee Arthroplasty: A Systematic Review and Meta-analysis. Journal of the American Academy of Orthopaedic Surgeons Global Research and Reviews. 2021;5(7).

4. Guerin G, Keel T, Tollefson L, Shoemaker EP, Slette EL, Jacobson NJ, et al. Excision of Femoral Heterotopic Ossification and Lysis of Adhesions After Medial Collateral Ligament Reconstruction. Arthroscopy Techniques. 2025;14(3).

5. Iorio R, Healy WL. Heterotopic ossification after hip and knee arthroplasty: risk factors, prevention, and treatment. J Am Acad Orthop Surg. 2002;10(6):409-16.

6. James EW, Taber CE, Marx RG. Complications Associated with Posterior Cruciate Ligament Reconstruction and Avoiding Them. Journal of Knee Surgery. 2021;34(06):587-91.

7. Lubowitz JH, Elson W, Guttmann D. Complications in the treatment of medial and lateral sided injuries of the knee joint. Sports Medicine and Arthroscopy Review. 2006;14(1):51-5.

8. Manrique J, Gomez MM, Parvizi J. Stiffness after Total Knee Arthroplasty. Journal of Knee Surgery. 2015;28(2):119-26.

9. Nelson CL, Kim J, Lotke PA. Stiffness After Total Knee Arthroplasty. JBJS. 2005;87(1):264-70.

10. Rader CP, Barthel T, Haase M, Scheidler M, Eulert J. Heterotopic ossification after total knee arthroplasty. 54/615 cases after 1-6 years' follow-up. Acta Orthop Scand. 1997;68(1):46-50.

11. Rodriguez AN, Schleck K, LaPrade RF. Complications of Medial and Lateral Knee Surgery and How to Best Avoid Them. Operative Techniques in Sports Medicine. 2022;30(2).

12. Rodríguez-Merchán EC, De la Corte-Rodríguez H, Encinas-Ullán CA, Gómez-Cardero P. Complications of surgical reconstruction of multiligament injuries of the knee joint: diagnosis, prevention and treatment. Efort Open Reviews. 2021;6(10):973-81.

13. Stannard JP, Wilson TC, Sheils TM, McGwin G, Volgas DA, Alonso JE. Heterotopic ossification associated with knee dislocation. Arthroscopy-the Journal of Arthroscopic and Related Surgery. 2002;18(8):835-9.

14. Sterner T, Saxler G, Barden B. Limited range of motion caused by heterotopic ossifications in primary total knee arthroplasty: a retrospective study of 27/191 cases. Archives of Orthopaedic and Trauma Surgery. 2005;125(3):188-92.

15. Tom JA, Miller MD. Complications in the multiple-ligament-injured knee. Operative Techniques in Sports Medicine. 2003;11(4):302-11.

16. Wharton MG, Shultz CL, Schenck RC, Jr., Richter DL. Evaluation and Management of Knee Dislocations. J Am Acad Orthop Surg. 2026;34(4):e477-e87.

17. Whelan DB, Dold AP, Trajkovski T, Chahal J. Risk Factors for the Development of Heterotopic Ossification After Knee Dislocation. Clinical Orthopaedics and Related Research. 2014;472(9):2698-704.

18. Zeckey C, Hildebrand F, Frink M, Krettek C. Heterotopic ossifications following implant surgery-epidemiology, therapeutical approaches and current concepts. Seminars in Immunopathology. 2011;33(3):273-86.
