# Supplementary material for: The prevention of heterotopic ossification around the knee: a scoping review
Source: BMC Musculoskelet Disord. 2026 Aug 1;27:651. doi: 10.1186/s12891-026-10318-w (PMC13428452; doi:10.1186/s12891-026-10318-w)
Supplement: Supplementary file 5 — Supplementary Material 5. [file 12891_2026_10318_MOESM5_ESM.docx]

**Supplement S5:** Study and participant characteristics of studies evaluating CPM for prophylaxis of HO around the knee.

| **First author, year** | **Country** | **Study type** | **JBI level of evidence** | **Participants receiving prophylaxis for HO around the knee / total enrolled** | **Knees analyzed / knees receiving prophylaxis** | **Index procedure / scenario** | **Indication / HO context (etiology / risk factors)** | **Follow-up (months)** | **Age (years)** | **Sex** |
| --- | --- | --- | --- | --- | --- | --- | --- | --- | --- | --- |
| Abdelfettah, 2012‡[1] | Morocco | Case series | 4.c | NR/17 | 11/11 (100.0%) | Surgery for existing HO | Neurogenic HO | NR | Mean: 31.6 (range: 19-41) | NR |
| Belhaj, 2013‡[2] | Morocco | Case series | 4.c | NR/19 | NR/NR | Surgery for existing HO | Neurogenic HO | NR | Mean: 31.5 (range: 19-44) | Male 16/19 (84.2%) Female 3/19 (15.8%) |
| Bellemans, 1999[3] | Belgium | Case report | 4.d | 1/1 (100.0%) | 1/1 (100.0%) | TKA | Primary prophylaxis, no risk factors reported | 18 | 83 | Female 1/1 (100.0%) |
| Dalury, 2004[4] | USA | Case series | 4.c | 465/465 (100.0%) | 500/500 (100.0%) | TKA | Primary prophylaxis, no risk factors reported | 24 in all patients | Mean: 68.6 (range: 54-84) | Male 176/465 (37.8%) Female 288/465 (61.9%) |
| Ippolito, 1999[5] | Italy | Case series | 4.c | 4/4 (100.0%) | 6/6 (100.0%) | Surgery for existing HO | Neurogenic HO | Mean: 34 (range: 25-60)† | Mean: 28.8 (range: 19-42)† | Male 3/4 (75.0%) Female 1/4 (25.0%) |
| Matsumoto, 1999[6] | Japan | Case report | 4.d | 2/2 (100.0%) | 2/2 (100.0%) | Surgery for existing HO | Posttraumatic HO | 72, 120 | 21, 56 | Male 2/2 (100.0%) |
| Mills, 2003[7] | USA | Case series | 4.c | 35/35 (100.0%) | 36/36 (100.0%) | Knee dislocation | Primary prophylaxis | 12 | NR | NR |
| Pham, 1997[8] | USA | Case report | 4.d | 1/1 (100.0%) | 1/1 (100.0%) | TKA | Primary prophylaxis | 6 | 52 | Male 1/1 (100.0%) |

Values are reported as n/N (%) unless otherwise specified. Continuous variables are preferentially presented as mean (range). If unavailable mean ± SD or median (IQR/range) is reported according to the original publications. “Participants receiving prophylaxis for HO around the knee / total enrolled” denotes the number of participants receiving the prophylaxis modality among all enrolled participants. “Knees analyzed / knees receiving prophylaxis” denotes the number of knees included in the analysis among knees receiving prophylaxis (if reported).

Abbreviations: CPM, continuous passive motion; HO, heterotopic ossification; JBI, Joanna Briggs Institute; NR, not reported; TKA, total knee arthroplasty; USA, United States of America.

† Values calculated from the reported data.

‡ Full text not available; data extracted from abstract only.

§ One non-adult participant excluded

**References:**

1. Abdelfettah Y, Cherqaoui D, El Anbari Y, Khadir A, Lmidmani F, Abdellatif EF (2012) Functional outcomes after surgery for neurogenic heterotopic ossifications: 17 cases collected at the Department of physical medicine and rehabilitation, Casablanca University Hospital. Annals of Physical and Rehabilitation Medicine. 55(e173-e174+e176. doi:10.1016/j.rehab.2012.07.450.

2. Belhaj K, Abdelfattah Y, Khadir A, Kassimi E, Lmidmani F, El Fatimi A (2013) Functional results of surgery neurogenic heterotopic ossification in patients with severe traumatic brain injury: About 19 cases. Annals of Physical and Rehabilitation Medicine. 56(e406. doi:10.1016/j.rehab.2013.07.1043.

3. Bellemans J, Claerhout P, Eid T, Fabry G (1999) Severe heterotopic ossifications after total knee arthroplasty. Acta Orthop Belg. 65(1):98-101.

4. Dalury DF, Jiranek WA (2004) The incidence of heterotopic ossification after total knee arthroplasty. Journal of Arthroplasty. 19(4):447-452. doi:10.1016/j.arth.2003.12.064.

5. Ippolito E, Formisano R, Farsetti P, Caterini R, Penta F (1999) Excision for the treatment of periarticular ossification of the knee in patients who have a traumatic brain injury. Journal of Bone and Joint Surgery-American Volume. 81A(6):783-789. doi:10.2106/00004623-199906000-00005.

6. Matsumoto H, Kawakubo M, Otani T, Fujikawa K (1999) Extensive post-traumatic ossification of the patellar tendon. A report of two cases. J Bone Joint Surg Br. 81(1):34-36. doi:10.1302/0301-620x.81b1.9074.

7. Mills WJ, Tejwani N (2003) Heterotopic ossification after knee dislocation: the predictive value of the injury severity score. J Orthop Trauma. 17(5):338-345. doi:10.1097/00005131-200305000-00004.

8. Pham J, Kumar R (1997) Heterotopic ossification after total knee arthroplasty. Am J Orthop (Belle Mead NJ). 26(2):141-143.
