# Supplementary material for: The prevention of heterotopic ossification around the knee: a scoping review
Source: BMC Musculoskelet Disord. 2026 Aug 1;27:651. doi: 10.1186/s12891-026-10318-w (PMC13428452; doi:10.1186/s12891-026-10318-w)
Supplement: Supplementary file 6 — Supplementary Material 6. [file 12891_2026_10318_MOESM6_ESM.docx]

**Supplement S6:** Treatment characteristics and outcomes of studies evaluating CPM for prophylaxis of HO around the knee.

| **First author, year** | **Further details on dose and schedule** | **Timing and duration** | **Co-interventions** | **Any new HO, n/N (%)** | **Clinically relevant HO, n/N (%)** | **Knees needing further interventions for HO** | **ROM flex-ext** | **PROMs** | **Pain** | **Return to work / activity** | **Adverse events potentially related to prophylaxis** |
| --- | --- | --- | --- | --- | --- | --- | --- | --- | --- | --- | --- |
| Abdelfettah, 2012‡[1] | NR | NR | Physical therapy | NR | NR | Manipulation: NR  revision: NR | NR | NR | NR | Recovered ability to walk: 1/11 (9.1%)  Achieved a good sitting position: 10/11 (90.9%) | NR |
| Belhaj, 2013‡[2] | NR | NR | Physical therapy | NR | NR | Manipulation: NR  revision: NR | Pre-op: mean 45° (10°–80°) post-op: mean improvement: 46° | NR | NR | NR | NR |
| Bellemans, 1999[3] | NR | Started on d3 after surgery | Physical therapy, and during maturation of HO: RT 3x2 Gy and Indomethacin 100 mg per day for 3 months | 1/1 (100.0%) | 1/1 (100.0%) | Manipulation: 0/1 (0.0%)  revision: 0/1 (0.0%) | Pre-op: 110°  post-op: 115° | NR | No pain at final follow-up | Comfortable walking without pain: 1/1 (100.0%) | NR |
| Dalury, 2004[4] | Approximately 8h a day | Started in the recovery room, used until discharge (usually d3) | Cryotherapy and physical therapy | 76/500 (15.2%)§ | 7/500 (1.4%) | Manipulation: 7/500 (1.4%)  revision: 0/500 (0.0%) | Pre-op: mean: 100°†  Post-op: patients without HO: mean: 116°†, HO < 2 cm: mean: 113°†, HO > 2 cm: mean: 114°†, HO > 5cm: mean: 96°† | NR | NR | NR | NR |
| Ippolito, 1999[5] | Continuous, only stopped for active therapy | Started immediately after surgery, used for 6 weeks | Physical therapy | 0/4 (0.0%) | 0/4 (0.0%) | Manipulation: 0/4 (0.0%)  revision: 0/4 (0.0%) | Pre-op: mean: 38.3° (range: 20-70°)†  post-op: mean: 109.2° (90-130°)† | NR | NR | Recovered ability to walk: 4/4 (100.0%) | Wound dehiscence in one case |
| Matsumoto, 1999[6] | NR | NR | NR | 0/2 (0.0%) | 0/2 (0.0%) | Manipulation: 0/2 (0.0%)  revision: 0/2 (0.0%) | Pre-op: 100°, 130°  post-op: 120°, NR | NR | Pain-free at last follow-up 2/2 (100.0%) | Yes: 2/2 (100.0%) | NR |
| Mills, 2003[7] | NR | Started immediately after surgery, unless prohibited by the patient's injuries | Immobilization in extension, if CPM was not possible immediately  Outpatient physical therapy | 15/36 (41.7%) | 6/36 (16.7%) | Manipulation: 5/36 (13.9%)  HO excision with post-operative RT: 3/36 (8.3%) | Pre-op: NR  post-op: mean: 105.2°† | NR | NR | NR | Wound complications in 4/36 (11.1%), with 2 cases of infection and 2 cases of wound dehiscence |
| Pham, 1997[8] | NR | NR | Physical therapy | 1/1 (100.0%) | 1/1 (100.0%) | HO excision with post-operative Indomethacin 1/1 (100.0%) | Pre-op: NR  post-op: 50° | NR | NR | Ambulation limited 1/1 (100.0%) | NR |

Values are reported as n/N (%) unless otherwise specified. Continuous variables are preferentially presented as mean (range). If unavailable mean ± SD or median (IQR/range) is reported according to the original publications. “Any new HO” and “clinically relevant HO” were extracted as defined in the original publications. If “clinically relevant HO” was not explicitly defined by the authors, we considered HO as clinically relevant if it was reported as symptomatic and/or required further intervention. ROM flex-ext indicates flexion–extension range of motion (degrees).

Abbreviations: CPM, continuous passive motion; HO, heterotopic ossification; NR, not reported; PROMs, patient-reported outcome measures; ROM, range of motion; RT, radiotherapy.

† Values calculated from the reported data.

‡ Full text not available; data extracted from abstract only.

§ Only HO >1cm diameter reported

**References**

1. Abdelfettah Y, Cherqaoui D, El Anbari Y, Khadir A, Lmidmani F, Abdellatif EF (2012) Functional outcomes after surgery for neurogenic heterotopic ossifications: 17 cases collected at the Department of physical medicine and rehabilitation, Casablanca University Hospital. Annals of Physical and Rehabilitation Medicine. 55(e173-e174+e176. doi:10.1016/j.rehab.2012.07.450.

2. Belhaj K, Abdelfattah Y, Khadir A, Kassimi E, Lmidmani F, El Fatimi A (2013) Functional results of surgery neurogenic heterotopic ossification in patients with severe traumatic brain injury: About 19 cases. Annals of Physical and Rehabilitation Medicine. 56(e406. doi:10.1016/j.rehab.2013.07.1043.

3. Bellemans J, Claerhout P, Eid T, Fabry G (1999) Severe heterotopic ossifications after total knee arthroplasty. Acta Orthop Belg. 65(1):98-101.

4. Dalury DF, Jiranek WA (2004) The incidence of heterotopic ossification after total knee arthroplasty. Journal of Arthroplasty. 19(4):447-452. doi:10.1016/j.arth.2003.12.064.

5. Ippolito E, Formisano R, Farsetti P, Caterini R, Penta F (1999) Excision for the treatment of periarticular ossification of the knee in patients who have a traumatic brain injury. Journal of Bone and Joint Surgery-American Volume. 81A(6):783-789. doi:10.2106/00004623-199906000-00005.

6. Matsumoto H, Kawakubo M, Otani T, Fujikawa K (1999) Extensive post-traumatic ossification of the patellar tendon. A report of two cases. J Bone Joint Surg Br. 81(1):34-36. doi:10.1302/0301-620x.81b1.9074.

7. Mills WJ, Tejwani N (2003) Heterotopic ossification after knee dislocation: the predictive value of the injury severity score. J Orthop Trauma. 17(5):338-345. doi:10.1097/00005131-200305000-00004.

8. Pham J, Kumar R (1997) Heterotopic ossification after total knee arthroplasty. Am J Orthop (Belle Mead NJ). 26(2):141-143.
