# Supplementary material for: The prevention of heterotopic ossification around the knee: a scoping review
Source: BMC Musculoskelet Disord. 2026 Aug 1;27:651. doi: 10.1186/s12891-026-10318-w (PMC13428452; doi:10.1186/s12891-026-10318-w)
Supplement: Supplementary file 7 — Supplementary Material 7. [file 12891_2026_10318_MOESM7_ESM.docx]

**Supplement S7:** Study and participant characteristics of studies evaluating pharmacological prophylaxis of HO around the knee.

| **First author, year** | **Country** | **Study type** | **JBI level of evidence** | **Participants receiving prophylaxis for HO around the knee / total enrolled** | **Knees analyzed / knees receiving prophylaxis** | **Index procedure / scenario** | **Indication / HO context (etiology / risk factors)** | **Follow-up (months)** | **Age (years)** | **Sex** |
| --- | --- | --- | --- | --- | --- | --- | --- | --- | --- | --- |
| Alturki, 2020[1] | Saudi Arabia | Case report | 4.d | 1/1 (100.0%) | 1/1 (100.0%) | Removal of HO and TKA revision after TKA | Recurrence prophylaxis | 4 | 32 | Female 1/1 (100.0%) |
| Bragg, 2022[2] | USA | Case report | 4.d | 1/1 (100.0%) | 1/1 (100.0%) | Removal of HO after ACL reconstruction | Recurrence prophylaxis | 3 | 32 | Female 1/1 (100.0%) |
| Charnley, 1996[3] | France | Case series | 4.c | 6/6 (100.0%) | 7/7 (100.0%) | Removal of neurogenic HO | Recurrence prophylaxis | Mean: 20.3 (range: 12-36) | Mean: 30.8 (range: 20-43) | Male 6/6 (100.0%) |
| Cho, 2011[4] | South Korea | Case report | 4.d | 1/1 (100.0%) | 1/1 (100.0%) | MLKI with multiple fractures | Primary prophylaxis | 12 | 50 | Female 1/1 (100.0%) |
| Daniilidis, 2013[5] | Germany | Case report | 4.d | 1/1 (100.0%) | 1/1 (100.0%) | Removal of HO after surgery for patellar fracture | Recurrence prophylaxis | 12 | 23 | Male 1/1 (100.0%) |
| Erdil, 2012[6] | Turkey | Case report | 4.d | 1/1 (100.0%) | 1/1 (100.0%) | Removal of HO after previous ACL | Recurrence prophylaxis | 36 | 36 | Male 1/1 (100.0%) |
| Firoozabadi, 2025[7] | Iran | Case report | 4.d | 1/1 (100.0%) | 1/1 (100.0%) | Removal of HO after tibia fracture nailing | Recurrence prophylaxis | 24 | 38 | Male 1/1 (100.0%) |
| Fuller, 2005[8] | USA | Case series | 4.c | 17/17 (100.0%) | 22/22 (100.0%) | Removal of neurogenic HO | Recurrence prophylaxis | Mean: 32 (range: 10-98) | Mean: 32.6 (range: 19-51)† | Male 10/17 (58.8%)  Female 7/17 (41.2%) |
| Hari Krishnan, 2016[9] | India | Case report | 4.d | 1/1 (100.0%) | 1/1 (100.0%) | Removal of HO after intramedullary nailing of the tibia | Recurrence prophylaxis | 6 | 24 | Male 1/1 (100.0%) |
| Hoffer, 2024[10] | USA | Case report | 4.d | 1/1 (100.0%) | 1/1 (100.0%) | Removal of HO after intramedullary nailing of the tibia and femur | Recurrence prophylaxis | 3 | 24 | Female 1/1 (100.0%) |
| Liu, 2022[11] | China | Case report | 4.d | 1/1 (100.0%) | 1/1 (100.0%) | Removal of HO after patellar and tibial tuberosity fracture fixation | Recurrence prophylaxis | 12 | 61 | Male 1/1 (100.0%) |
| Mitsionis, 2009[12] | Greece | Case series | 4.c | 14/14 (100.0%) | 23/23 (100.0%) | Removal of neurogenic HO | Recurrence prophylaxis | Mean: NR (range: 17-52) | Mean: 32 (range: 18-66) | Male 13/14 (92.9%)  Female 1/14 (7.1%) |
| Ogilvie-Harris, 1995[13] | Canada | Case series | 4.c | 4/4 (100.0%) | 4/4 (100.0%) | Removal of HO after ACL reconstruction | Recurrence prophylaxis | Mean: NR (range: 6-NR) | 23, 24, NR, NR | Male NR  Female ≥1/4 (≥25%) |
| Parvizi, 2001[14] | USA | Case series | 4.c | 1/20 (5.0%) | 1/1 (100.0%) | Primary TKA | Primary prophylaxis due to HO after THA | Mean: 134.4 (range: 36-192)†* | Mean: 55 (range: 28-67)* | Male 17/20 (85.0%)*  Female 3/20 (15.0%)* |
| Pham, 1997[15] | USA | Case report | 4.d | 1/1 (100.0%) | 1/1 (100.0%) | Removal of HO after TKA | Recurrence prophylaxis | 19 | 52 | Male 1/1 (100.0%) |
| Rader, 1997[16] | Germany | Case series | 4.c | 2/558 (0.4%) | 2/2 (100.0%) | Removal of HO after TKA | Recurrence prophylaxis | Mean: 28 (range: 12-72)* | Mean: 70 (range: 48-92)* | Male 120/558 (21.5%)*  Female 438/558 (78.5%)* |
| Subbarao, 1987[17] | USA | Case series | 4.c | 1/5 (20.0%) | 2/2 (100.0%) | Removal of neurogenic HO | Recurrence prophylaxis | Mean: 21.4 (range: 8-35)* | Mean: 35.2 (range: 29-41)* | NR |
| Valencia, 2007[18] | Spain | Case report | 4.d | 1/1 (100.0%) | 1/1 (100.0%) | Removal of HO after ACL reconstruction | Recurrence prophylaxis | 6 | 29 | Male 1/1 (100.0%) |
| Van Nest, 2021[19] | USA | Comparative cohort study | 3.c | 747/1051 (71.1%) | 747/747 (100.0%) | TKA | Primary prophylaxis | Mean: 11 (range: NR) | Mean: 67.2 ± 8.8 | Male: 304/747 (40.7%)  Female: 443/747 (59.3%) |
| Wróblewski, 2013[20] | Poland | Case report | 4.d | 1/1 (100.0%) | 1/1 (100.0%) | Removal of HO and revision TKA after TKA infection | Recurrence prophylaxis | 7 | 68 | Female 1/1 (100.0%) |
| Zhang, 2014[21] | China | Case report | 4.d | 1/1 (100.0%) | 2/2 (100.0%) | Removal of neurogenic HO | Recurrence prophylaxis | 18 | 47 | Male 1/1 (100.0%) |

Values are reported as n/N (%) unless otherwise specified. Continuous variables are preferentially presented as mean (range). If unavailable mean ± SD or median (IQR/range) is reported according to the original publications. “Participants receiving prophylaxis for HO around the knee / total enrolled” denotes the number of participants receiving the prophylaxis modality among all enrolled participants. “Knees analyzed / knees receiving prophylaxis” denotes the number of knees included in the analysis among knees receiving prophylaxis (if reported).

Abbreviations: ACL, anterior cruciate ligament; HO, heterotopic ossification; JBI, Joanna Briggs Institute; MLKI, multi-ligament knee injury; NR, not reported; NSAID, non-steroidal anti-inflammatory drug; THA, total hip arthroplasty; TKA, total knee arthroplasty; USA, United States of America.

* Values reported for the entire cohort; no separate data for the prophylaxis subgroup were provided.

† Values calculated from the reported data.

**References:**

1. Alturki AA, Aldeghaither SA, Alhandi AA (2020) Severe heterotopic ossification post total knee arthroplasty in a patient with rheumatoid arthritis: a case report. J Surg Case Rep. 2020(3):rjz390. doi:10.1093/jscr/rjz390.

2. Bragg JT, Hayes-Lattin M, Shields MV, Salzler MJ (2022) Heterotopic Ossification After Anterior Cruciate Ligament Reconstruction With Quadriceps Tendon Autograft: A Case Report. JBJS Case Connect. 12(4):doi:10.2106/jbjs.Cc.22.00445.

3. Charnley G, Judet T, deLoubresse CG, Mollaret O (1996) Excision of heterotopic ossification around the knee following brain injury. Injury-International Journal of the Care of the Injured. 27(2):125-128. doi:10.1016/0020-1383(95)00180-8.

4. Cho SH, Hwang SC, Kim KI, Yoo JH (2011) A case of extensive heterotopic ossification following multiple ligament reconstruction after severe knee trauma. European Journal of Orthopaedic Surgery and Traumatology. 21(6):435-437. doi:10.1007/s00590-010-0730-5.

5. Daniilidis K, Vogt B, Raschke MJ (2013) Symptomatic heterotopic ossification: seven years after patella fracture. Musculoskelet Surg. 97(2):169-171. doi:10.1007/s12306-011-0160-5.

6. Erdil M, Asik M, Sen C, Polat G (2012) Heterotopic bone formation following anterior cruciate ligament reconstruction with BPTB autograft. Acta Orthopaedica Et Traumatologica Turcica. 46(1):72-76. doi:10.3944/aott.2012.2529.

7. Firoozabadi AM, Rezaee H, Razzaghof M, Mortazavi SMJ (2025) Functional outcomes may vary over time after patellar tendon and knee intra-articular heterotopic ossification excision: A case report. Int J Surg Case Rep. 127(110773. doi:10.1016/j.ijscr.2024.110773.

8. Fuller DA, Mark A, Keenan MA (2005) Excision of heterotopic ossification from the knee: a functional outcome study. Clin Orthop Relat Res. 438(197-203. doi:10.1097/00003086-200509000-00033.

9. Hari Krishnan B, Sharma Y, Prabhakara A (2016) A rare case of intra-articular heterotopic ossification of knee following intra-medullary nailing of fracture tibia in a patient with fat embolism. Med J Armed Forces India. 72(Suppl 1):S115-s119. doi:10.1016/j.mjafi.2016.01.004.

10. Hoffer AJ, Lin EA, Kalani MA, Lyons MK, Richardson M (2024) Excision of Intra-articular Knee Heterotopic Ossification Using a 70° Arthroscope. Case Reports in Orthopedics. 2024(doi:10.1155/2024/9998388.

11. Liu Q, Tang D, Zhu W, Chen Y (2022) Patellar Tendon Reconstruction Using Autologous Hamstring Tendons for the Treatment of Extensive Patellar Tendon Ossification. Orthop Surg. 14(11):3119-3124. doi:10.1111/os.13435.

12. Mitsionis GI, Lykissas MG, Kalos N, Paschos N, Beris AE, Georgoulis AD et al (2009) Functional outcome after excision of heterotopic ossification about the knee in ICU patients. International Orthopaedics. 33(6):1619-1625. doi:10.1007/s00264-008-0618-8.

13. Ogilvie-Harris DJ, Sekyi-Otu A (1995) Periarticular heterotopic ossification: a complication of arthroscopic anterior cruciate ligament reconstruction using a two-incision technique. Arthroscopy. 11(6):676-679.

14. Parvizi J, Duffy GP, Trousdale RT (2001) Total knee arthroplasty in patients with ankylosing spondylitis. Journal of Bone and Joint Surgery-American Volume. 83A(9):1312-1316. doi:10.2106/00004623-200109000-00003.

15. Pham J, Kumar R (1997) Heterotopic ossification after total knee arthroplasty. Am J Orthop (Belle Mead NJ). 26(2):141-143.

16. Rader CP, Barthel T, Haase M, Scheidler M, Eulert J (1997) Heterotopic ossification after total knee arthroplasty. 54/615 cases after 1-6 years' follow-up. Acta Orthop Scand. 68(1):46-50. doi:10.3109/17453679709003974.

17. Subbarao JV, Nemchausky BA, Gratzer M (1987) Resection of heterotopic ossification and Didronel therapy--regaining wheelchair independence in the spinal cord injured patient. J Am Paraplegia Soc. 10(1):3-7. doi:10.1080/01952307.1987.11719626.

18. Valencia H, Gavín C (2007) Infrapatellar heterotopic ossification after anterior cruciate ligament reconstruction. Knee Surgery Sports Traumatology Arthroscopy. 15(1):39-42. doi:10.1007/s00167-006-0131-9.

19. Van Nest DS, Clarkson S, Chisari E, Sherman MB, Parvizi J (2021) Low-Dose Aspirin Administered for Venous Thromboembolism Prophylaxis Reduces the Incidence of Heterotopic Ossification in Total Joint Arthroplasty. Journal of Arthroplasty. 36(5):1543-1547. doi:10.1016/j.arth.2020.12.021.

20. Wróblewski R, Pokrzywnicka-Gajek I, Kowalczewski J (2013) Heterotopic ossifications after two-stage septic revision knee arthroplasty in a rheumatoid arthritis patient. Reumatologia. 51(5):389-393. doi:10.5114/reum.2013.38392.

21. Zhang X, Jie S, Liu T, Zhang X (2014) Acquired heterotopic ossification in hips and knees following encephalitis: case report and literature review. BMC Surg. 14(74. doi:10.1186/1471-2482-14-74.
