# Supplementary material for: The prevention of heterotopic ossification around the knee: a scoping review
Source: BMC Musculoskelet Disord. 2026 Aug 1;27:651. doi: 10.1186/s12891-026-10318-w (PMC13428452; doi:10.1186/s12891-026-10318-w)
Supplement: Supplementary file 8 — Supplementary Material 8. [file 12891_2026_10318_MOESM8_ESM.docx]

**Supplement S8.** Treatment characteristics and outcomes of studies evaluating pharmacological prophylaxis of HO around the knee.

| **First author, year** | **Further details on dose and schedule** | **Timing and duration** | **Co-interventions** | **Any new HO, n/N (%)** | **Clinically relevant HO, n/N (%)** | **Knees needing further interventions for HO** | **ROM flex-ext** | **PROMs** | **Pain** | **Return to work / activity** | **Adverse events potentially related to prophylaxis** |
| --- | --- | --- | --- | --- | --- | --- | --- | --- | --- | --- | --- |
| Alturki, 2020[1] | Indomethacin, dose and schedule: NR | Post-op, duration: 6 weeks | Physical therapy, patient received DMARDs due to rheumatoid arthritis | 0/1 (0.0%) | 0/1 (0.0%) | 0/1 (0.0%) | Pre-op: 0°  post-op: 70° | NR | NR | NR | NR |
| Bragg, 2022[2] | Indomethacin, 75 mg daily | Started 1 week before surgery, duration: 7 weeks | ACL-reconstruction specific rehabilitation | 0/1 (0.0%) | 0/1 (0.0%) | 0/1 (0.0%) | Pre-op: 111°  post-op: symmetric ROM, similar to the unaffected knee | NR | Pre-op: yes, intensity NR  post-op: none | NR | NR |
| Charnley, 1996[3] | Indomethacin, dose and schedule: NR | Timing and duration: NR | Physical therapy and cryotherapy in all patients, additional RT in one patient | 0/7 (0.0%) | 0/7 (0.0%) | 0/7 (0.0%) | Pre-op: mean: 55° (range: 30-80°)†  post-op: mean: 128° (range: 105-140°)† | NR | Pre-op: NR  post-op: None 7/7 (100.0%) | Marked improvement in mobility 3/6 (50.0%) | No delayed wound healing 1/1 (100.0%) |
| Cho, 2011[4] | Etidronate, dose and schedule: NR | Post-op, duration: 5 weeks | Knee immobilized in a splint for 2 weeks, afterwards guided rehabilitation | 1/1 (100.0%) | 1/1 (100.0%) | 0/1 (0.0%) | Pre-op: NR  post-op: 50° | NR | NR | Resumed activities of daily living 1/1 (100.0%) | NR |
| Daniilidis, 2013[5] | Indomethacin, 75 mg daily | Post-op, duration: 4 weeks | NR | 0/1 (0.0%) | 0/1 (0.0%) | 0/1 (0.0%) | Pre-op: NR  post-op: NR | NR | Preop: pain prohibiting sport  postop: NR | Return to his previous level of daily activities 1/1 (100.0%) | None 1/1 (100.0%) |
| Erdil, 2012[6] | Indomethacin 35 mg TID | Started post-op, duration: 6 weeks | Immobilization in full extension for 3 days, then physical therapy | 0/1 (0.0%) | 0/1 (0.0%) | 0/1 (0.0%) | Pre-op: 80°  post-op: 135° | Pre-op: Lysholm score 79 points, Tegner activity level: 4 points, IKDC score: D  post-op: Lysholm score 95 points, Tegner activity level: 9 points, IKDC score: A | Pre-op: none  post-op: none | NR | NR |
| Firoozabadi, 2025[7] | Indomethacin 25 mg TID | Started post-op, duration: 6 weeks | Physical therapy (not completed regularly by the patient) | 1/1 (100.0%) | 1/1 (100.0%) | 1/1 (100.0%) | Pre-op: 45°  post-op: 80° | NR | Pain-free at last follow-up | NR | NR |
| Fuller, 2005[8] | Etidronate 20 mg/kg once daily | Post-op, duration: 2 months | Two patients additionally received 1 x 7.0 Gy RT one day post-op | 0/22 (0.0%) | 0/22 (0.0%) | 0/22 (0.0%) | Pre-op: mean: 41° (range: 0-105°)  post-op: mean: 106° (range: 70-150°) | Pre-op sitting ability:  easy: 5/17 (29.4%), difficult: 4/17 (23.5%)  unable: 8/17 (47.1%)  post-op sitting ability:  easy: 16/17 (94.1%), difficult: 1/17 (5.9%) | NR | Pre-op: ambulation:  requiring supervision: 1/17 (5.9%), unable: 16/17 (94.1%)  post-op: ambulation: independent: 10/17 (58.8%), requiring supervision: 1/17 (5.9%)  requiring assistance: 3/17 (17.6%)  unable: 3/17 (17.6%) | Wound infection: 2/22 (9.1%)  No cases of fractures, knee instability, nerve or vascular injuries |
| Hari Krishnan, 2016[9] | Indomethacin dose and schedule: NR | Started post-op, duration: NR | Physical therapy | 0/1 (0.0%) | 0/1 (0.0%) | 0/1 (0.0%) | Pre-op: NR  post-op: normal ROM | NR | NR | NR | NR |
| Hoffer, 2024[10] | Indomethacin 25 mg daily | Started post-op, duration: NR | Thrombosis prophylaxis with aspirin 81 mg daily, physical therapy and resistance training | 0/1 (0.0%) | 0/1 (0.0%) | 0/1 (0.0%) | Pre-op: 130°  post-op: 135° | NR | Pre-op: yes, intensity NR  post-op: none | Return to sports, competing in amateur level CrossFit | No immediate postoperative complications |
| Liu, 2022[11] | Celecoxib 200 mg, TID | Post-op, duration: 2 weeks | Use of a knee brace, passive ROM exercise until week 6, afterwards active ROM exercises | NR | 0/1 (0.0%) | 0/1 (0.0%) | Pre-op: 30°  post-op: 115° | Pre-op: HSS: 48, Lysholm score: 53  post-op: HSS: 95, Lysholm score: 90 | Pre-op: NR  post-op: none | Able to resume daily activities | No gastrointestinal AEs. |
| Mitsionis, 2009[12] | Indomethacin 75 mg daily | Post-op, duration: 15 days | Patients with extensive soft-tissue detachment during surgery received 1 x 7.0 Gy post-op: NR/23 | NR | NR | NR | Pre-op: mean: 34.3° (range: 10-50°)  post-op: mean: 76.9° (range: 20-110°) | Pre-op: sitting: difficult: 10/14 (71.4%)  unable: 4/14 (28.6%)  post-op: sitting: easy: 12/14 (85.7%)  difficult: 2/14 (14.3%) | NR | Pre-op: ambulation:  unable: 14/14 (100.0%)  post-op: ambulation: independent: 8/14 (57.1%), requiring supervision: 1/14 (7.1%)  requiring assistance: 4/14 (28.6%)  unable: 1/14 (7.1%) | NR |
| Ogilvie-Harris, 1995[13] | Indomethacin, dose and schedule: NR | Started pre-op, continued for 3 months post-op | NR | 0/4 (0.0%) | 0/4 (0.0%) | 0/4 (0.0%) | Pre-op: NR 4/4 (100.0%)  post-op: improved ROM 4/4 (100.0%) | NR | Pre-op: yes, intensity NR: NR/4  post-op: none 4/4 (100.0%) | NR | NR |
| Parvizi, 2001[14] | Ibuprofen, dose and schedule: NR | Timing and duration: NR | NR | 0/1 (0.0%) | 0/1 (0.0%) | 0/1 (0.0%) | Pre-op: mean: 84.8° (range: 25°-130°)*  post-op: mean: 86.7° (range: 40°-125°)* | Preop: KSS function: mean: 16.3 (range: 0–40)* post-op: mean: 58.7 (range: 0–85)* | Preop: KSS pain: mean: 14 (range: 0–37)*  post-op: mean: 76.3 (range: 45–100)* | Pre-op: walking:  with crutches full-time: 12/20 (60.0%)*  with walker: 6/20 (30.0%)*  non-ambulatory; 2/20 (10.0%)*  post-op:  without aids: 3/20 (15.0%)*  crutches part-time: 8/20 (40.0%)*  crutches full-time: 3/20 (15.0%)*  with walker: 4/20 (20.0%)*  non-ambulatory: 2/20 (10.0%)* | Superficial wound infection: 1/20 (5.0%)*  Hematoma 1/20 (5.0%)* |
| Pham, 1997[15] | Indomethacin, dose and schedule: NR | Post-op, duration: 2 weeks | Physical therapy | 1/1 (100.0%) | 0/1 (0.0%) | 0/1 (0.0%) | Pre-op: 85°  post-op: 110° | NR | Pre-op: yes, intensity: NR  post-op: none | Regained ability to ambulate, climb and descent stairs without difficulty. | NR |
| Rader, 1997[16] | Indomethacin 50 mg, BID | Post-op, duration: 2 months | NR | 0/2 (0.0%) | 0/2 (0.0%) | 0/2 (0.0%) | NR | NR | NR | NR | NR |
| Subbarao, 1987[17] | Pre-op: etidronate 20 mg/kg once daily  post-op: stopped for 72 h, afterwards: etidronate 10 mg/kg once daily | Pre-op: 10-14 days  post-op: 3-11 months | Physical therapy with passive ROM exercises | NR | NR | NR | Pre-op: NR  post-op: 70°, 90° | NR | NR | Recovered ability to function in a semi-reclining wheelchair | Local suture line infection 3/8 (37.5%)*  Deep seated hematoma 1/8 (12.5%)*  Chronic drainage from the left knee 1/8 (12.5%)*  No etidronate related adverse events |
| Valencia, 2007[18] | Indomethacin, dose and schedule: NR | Post-op, duration: 6 weeks | NR | 0/1 (0.0%) | 0/1 (0.0%) | 0/1 (0.0%) | NR | NR | Pre-op: yes, intensity: NR  post-op: none | Unrestricted working and leisure activities | NR |
| Van Nest, 2021[19] | IG: ASA, dose: 81 mg or 325 mg, TID | IG: Post-op: duration 4 weeks | Tranexamic acid was used more often in the IG: (41.3% vs 31.6%, p = 0.023) | IG: 100/747 (13.4%) | IG: NR | IG: NR | IG: NR | IG: NR | IG: NR | IG: NR | IG: NR |
|  | CG: warfarin, enoxaparin, apixaban, rivaroxaban, dabigatran, and clopidogrel | CG: NR |  | CG: 56/304 (18.4%) | CG: NR | CG: NR | CG: NR | CG: NR | CG: NR | CG: NR | CG: NR |
| Wróblewski, 2013[20] | Indomethacin, dose and schedule: NR | Post-op, duration: NR (terminated early) | Physical therapy with active and passive ROM exercises, cryotherapy | 1/1 (100.0%) | 1/1 (100.0%) | 1/1 (100.0%) | Pre-op: 20°  post-op: 60° | NR | NR | NR | Elevated blood pressure leading to early termination of Indomethacin |
| Zhang, 2014[21] | Celecoxib 200 mg, once daily | Post-op, duration: 8 weeks | Physical therapy with active and passive ROM exercises, cryotherapy | 0/2 (0.0%) | 0/2 (0.0%) | 0/2 (0.0%) | Pre-op: 10°, 15°  post-op: 127°, 125° | NR | Pre-op: yes, intensity: NR  post-op: none | Regained ability to walk independently | NR |

Values are reported as n/N (%) unless otherwise specified. Continuous variables are preferentially presented as mean (range). If unavailable mean ± SD or median (IQR/range) is reported according to the original publications. “Any new HO” and “clinically relevant HO” were extracted as defined in the original publications. If “clinically relevant HO” was not explicitly defined by the authors, we considered HO as clinically relevant if it was reported as symptomatic and/or required further intervention. ROM flex-ext indicates flexion–extension range of motion (degrees).

Abbreviations: ACL, anterior cruciate ligament; ASA, acetylsalicylic acid; BID, twice daily; CG, control group; DMARDs, disease-modifying anti-rheumatic drugs; HO, heterotopic ossification; HSS, Hospital for Special Surgery score; IKDC, International Knee Documentation Committee score; KSS, Knee Society Score; NR, not reported; NSAID, non-steroidal anti-inflammatory drug; PROMs, patient-reported outcome measures; ROM, range of motion; RT, radiotherapy; TID, three times daily; VTE, venous thromboembolism; IG, as defined by original publication.

* Values reported for the entire cohort; no separate data for the prophylaxis subgroup were provided.

† Values calculated from the reported data.

**References:**

1. Alturki AA, Aldeghaither SA, Alhandi AA (2020) Severe heterotopic ossification post total knee arthroplasty in a patient with rheumatoid arthritis: a case report. J Surg Case Rep. 2020(3):rjz390. doi:10.1093/jscr/rjz390.

2. Bragg JT, Hayes-Lattin M, Shields MV, Salzler MJ (2022) Heterotopic Ossification After Anterior Cruciate Ligament Reconstruction With Quadriceps Tendon Autograft: A Case Report. JBJS Case Connect. 12(4):doi:10.2106/jbjs.Cc.22.00445.

3. Charnley G, Judet T, deLoubresse CG, Mollaret O (1996) Excision of heterotopic ossification around the knee following brain injury. Injury-International Journal of the Care of the Injured. 27(2):125-128. doi:10.1016/0020-1383(95)00180-8.

4. Cho SH, Hwang SC, Kim KI, Yoo JH (2011) A case of extensive heterotopic ossification following multiple ligament reconstruction after severe knee trauma. European Journal of Orthopaedic Surgery and Traumatology. 21(6):435-437. doi:10.1007/s00590-010-0730-5.

5. Daniilidis K, Vogt B, Raschke MJ (2013) Symptomatic heterotopic ossification: seven years after patella fracture. Musculoskelet Surg. 97(2):169-171. doi:10.1007/s12306-011-0160-5.

6. Erdil M, Asik M, Sen C, Polat G (2012) Heterotopic bone formation following anterior cruciate ligament reconstruction with BPTB autograft. Acta Orthopaedica Et Traumatologica Turcica. 46(1):72-76. doi:10.3944/aott.2012.2529.

7. Firoozabadi AM, Rezaee H, Razzaghof M, Mortazavi SMJ (2025) Functional outcomes may vary over time after patellar tendon and knee intra-articular heterotopic ossification excision: A case report. Int J Surg Case Rep. 127(110773. doi:10.1016/j.ijscr.2024.110773.

8. Fuller DA, Mark A, Keenan MA (2005) Excision of heterotopic ossification from the knee: a functional outcome study. Clin Orthop Relat Res. 438(197-203. doi:10.1097/00003086-200509000-00033.

9. Hari Krishnan B, Sharma Y, Prabhakara A (2016) A rare case of intra-articular heterotopic ossification of knee following intra-medullary nailing of fracture tibia in a patient with fat embolism. Med J Armed Forces India. 72(Suppl 1):S115-s119. doi:10.1016/j.mjafi.2016.01.004.

10. Hoffer AJ, Lin EA, Kalani MA, Lyons MK, Richardson M (2024) Excision of Intra-articular Knee Heterotopic Ossification Using a 70° Arthroscope. Case Reports in Orthopedics. 2024(doi:10.1155/2024/9998388.

11. Liu Q, Tang D, Zhu W, Chen Y (2022) Patellar Tendon Reconstruction Using Autologous Hamstring Tendons for the Treatment of Extensive Patellar Tendon Ossification. Orthop Surg. 14(11):3119-3124. doi:10.1111/os.13435.

12. Mitsionis GI, Lykissas MG, Kalos N, Paschos N, Beris AE, Georgoulis AD et al (2009) Functional outcome after excision of heterotopic ossification about the knee in ICU patients. International Orthopaedics. 33(6):1619-1625. doi:10.1007/s00264-008-0618-8.

13. Ogilvie-Harris DJ, Sekyi-Otu A (1995) Periarticular heterotopic ossification: a complication of arthroscopic anterior cruciate ligament reconstruction using a two-incision technique. Arthroscopy. 11(6):676-679.

14. Parvizi J, Duffy GP, Trousdale RT (2001) Total knee arthroplasty in patients with ankylosing spondylitis. Journal of Bone and Joint Surgery-American Volume. 83A(9):1312-1316. doi:10.2106/00004623-200109000-00003.

15. Pham J, Kumar R (1997) Heterotopic ossification after total knee arthroplasty. Am J Orthop (Belle Mead NJ). 26(2):141-143.

16. Rader CP, Barthel T, Haase M, Scheidler M, Eulert J (1997) Heterotopic ossification after total knee arthroplasty. 54/615 cases after 1-6 years' follow-up. Acta Orthop Scand. 68(1):46-50. doi:10.3109/17453679709003974.

17. Subbarao JV, Nemchausky BA, Gratzer M (1987) Resection of heterotopic ossification and Didronel therapy--regaining wheelchair independence in the spinal cord injured patient. J Am Paraplegia Soc. 10(1):3-7. doi:10.1080/01952307.1987.11719626.

18. Valencia H, Gavín C (2007) Infrapatellar heterotopic ossification after anterior cruciate ligament reconstruction. Knee Surgery Sports Traumatology Arthroscopy. 15(1):39-42. doi:10.1007/s00167-006-0131-9.

19. Van Nest DS, Clarkson S, Chisari E, Sherman MB, Parvizi J (2021) Low-Dose Aspirin Administered for Venous Thromboembolism Prophylaxis Reduces the Incidence of Heterotopic Ossification in Total Joint Arthroplasty. Journal of Arthroplasty. 36(5):1543-1547. doi:10.1016/j.arth.2020.12.021.

20. Wróblewski R, Pokrzywnicka-Gajek I, Kowalczewski J (2013) Heterotopic ossifications after two-stage septic revision knee arthroplasty in a rheumatoid arthritis patient. Reumatologia. 51(5):389-393. doi:10.5114/reum.2013.38392.

21. Zhang X, Jie S, Liu T, Zhang X (2014) Acquired heterotopic ossification in hips and knees following encephalitis: case report and literature review. BMC Surg. 14(74. doi:10.1186/1471-2482-14-74.
