# Supplementary material for: The prevention of heterotopic ossification around the knee: a scoping review
Source: BMC Musculoskelet Disord. 2026 Aug 1;27:651. doi: 10.1186/s12891-026-10318-w (PMC13428452; doi:10.1186/s12891-026-10318-w)
Supplement: Supplementary file 9 — Supplementary Material 9. [file 12891_2026_10318_MOESM9_ESM.docx]

**Supplement S9:** Study and participant characteristics of studies evaluating radiotherapy for prophylaxis of HO around the knee.

| **First author, year** | **Country** | **Study type** | **JBI level of evidence** | **Participants receiving prophylaxis for HO around the knee / total enrolled** | **Knees analyzed / knees receiving prophylaxis** | **Index procedure / scenario** | **Indication / HO context (etiology / risk factors)** | **Follow-up (months)** | **Age (years)** | **Sex** |
| --- | --- | --- | --- | --- | --- | --- | --- | --- | --- | --- |
| Balen, 2001[1] | USA | Case report | 4.d | 1/1 (100.0%) | 1/1 (100.0%) | Removal of HO after extensive electrical burns | Recurrence prophylaxis | NR | 34 | Male 1/1 (100.0%) |
| Baroudi, 2017[2] | Canada | Case report | 4.d | 1/1 (100.0%) | 1/1 (100.0%) | Removal of HO and TKA revision after TKA | Recurrence prophylaxis | 12 | 67 | Female 1/1 (100.0%) |
| Barrack, 2002[3] | Canada | Case series | 4.c | 1/135 (0.7%) | 1/1 (100.0%) | Removal of HO after revision TKA | Recurrence prophylaxis | NR | NR | NR |
| Brown, 2018[4] | UK | Case report | 4.d | 1/1 (100.0%) | 1/1 (100.0%) | Removal of HO and TKA revision after TKA | Recurrence prophylaxis | 12 | 78 | Male 1/1 (100.0%) |
| Chidel, 2001[5] | USA | Case series | 4.c | 5/5 (100.0%) | 6/6 (100.0%) | Removal of HO after previous TKA | Recurrence prophylaxis | Mean: 6.7 (range: 3-11)† | Mean: 66.2 (range: 37-78)† | Male 4/5 (80.0%)  Female: 1/5 (20.0%) |
| Cipriano, 2009[6] | USA | Case-control study | 3.d | 3/60 (5.0%) | 3/3 (100.0%) | Removal of neurogenic HO | Recurrence prophylaxis | Mean: 12.7 (range: 6-33)* | Mean: 36.7* | Male 23/30 (76.7%)*  Female: 7/30 (23.3%)* |
| Daugherty, 2013[7] | USA | Case series | 4.c | 12/12 (100.0%) | 12/12 (100.0%) | Mixed, including trauma surgery and TKA | Primary prophylaxis | Median: 78 (range: 1–132) | Mean: 45.1 (range: 32-62) † | Male 7/12 (58.3%)  Female: 5/12 (41.7%) |
| Davis, 2012[8] | USA | Case report | 4.d | 1/1 (100.0%) | 1/1 (100.0%) | Removal of HO, potentially caused by an episode of critical illness | Recurrence prophylaxis | 2 | 29 | Female 1/1 (100.0%) |
| Freije, 2021[9] | USA | Case series | 4.c | 1/287 (0.3%) | 1/1 (100.0%) | NR | NR | Median: 17.2 (range: 0-133)* | Median: 42 (range: 15-79)* | Male 186/287 (64.8%)*  Female: 101/287(35.2%)* |
| Gibson, 1997[10] | USA | Case report | 4.d | 1/1 (100.0%) | 1/1 (100.0%) | Removal of HO, caused by drug induced TEN | Recurrence prophylaxis | NR | 49 | Male 1/1 (100.0%) |
| Ivey, 1985[11] | USA | Case report | 4.d | 1/1 (100.0%) | 1/1 (100.0%) | Removal of HO, caused by repeated manipulation | Recurrence prophylaxis | 9 | 22 | Male 1/1 (100.0%) |
| Massaro, 2022[12] | Italy | Case report | 4.d | 1/1 (100.0%) | 1/1 (100.0%) | Removal of HO, after UKA | Recurrence prophylaxis | 9 | 42 | Female 1/1 (100.0%) |
| Mills, 2003[13] | USA | Case series | 4.c | 3/35 (8.6%) | 3/3 (100.0%) | Removal of HO, after surgery for knee dislocation | Recurrence prophylaxis | NR | NR | NR |
| Mishra, 2011[14] | USA | Case series | 4.c | 8/30 (26.7%) | 7/8 (87.5%) | Removal of HO | Recurrence prophylaxis | Median: 16 (range: 2-143)* | Mean: 47.5 (range: 15-78)* | Male 22/30 (73.3%)*  Female: 8/30(26.7%)* |
| Rosenberg, 2019[15] | USA | Case report | 4.d | 1/1 (100.0%) | 1/1 (100.0%) | TKA revision after infection and spacer implantation | Primary prophylaxis | ~60 | 61 | Female 1/1 (100.0%) |
| Ruiz Hernández, 2000[16] | Spain | Case report | 4.d | 1/1 (100.0%) | 2/2 (100.0%) | Removal of neurogenic HO | Recurrence prophylaxis | NR | 48 | Female 1/1 (100.0%) |
| Shah, 2023[17] | India | Case report | 4.d | 1/1 (100.0%) | 1/1 (100.0%) | Removal of HO | Recurrence prophylaxis | 24 | 36 | Male 1/1 (100.0%) |
| Stannard, 2002[18] | USA | Observational cohort study | 3.e | 3/55 (5.5%) | 3/3 (100.0%) | Removal of HO after surgery for MLKI | Recurrence prophylaxis | NR | Mean: 29 (range: 16-49)* | Male 36/55 (65.5%)*  Female: 19/55 (34.5%)* |

Values are reported as n/N (%) unless otherwise specified. Continuous variables are preferentially presented as mean (range). If unavailable mean ± SD or median (IQR/range) is reported according to the original publications. “Participants receiving prophylaxis for HO around the knee / total enrolled” denotes the number of participants receiving the prophylaxis modality among all enrolled participants. “Knees analyzed / knees receiving prophylaxis” denotes the number of knees included in the analysis among knees receiving prophylaxis (if reported).

Abbreviations: HO, heterotopic ossification; JBI, Joanna Briggs Institute; MLKI, multi-ligament knee injury; NR, not reported; RT, radiotherapy; TEN, toxic epidermal necrolysis; TKA, total knee arthroplasty; UK, United Kingdom; UKA, unicompartmental knee arthroplasty; USA, United States of America.

* Values reported for the entire cohort; no separate data for the prophylaxis subgroup were provided.

† Values calculated from the reported data.

**References:**

1. Balen PF, Helms CA (2001) Bony ankylosis following thermal and electrical injury. Skeletal Radiol. 30(7):393-397. doi:10.1007/s002560100342.

2. Baroudi M, Derome P, Malo M (2017) Severe heterotopic ossification and stiffness after revision knee surgery for a periprosthetic fracture. Arthroplast Today. 3(3):147-150. doi:10.1016/j.artd.2016.11.001.

3. Barrack RL, Brumfield CS, Rorabeck CH, Cleland D, Myers L (2002) Heterotopic ossification after revision total knee arthroplasty. Clinical Orthopaedics and Related Research. 404):208-213. doi:10.1097/01.blo.0000030497.43495.3f.

4. Brown A, Banerjee RD (2018) Severe heterotopic ossification following total knee replacement. Annals of the Royal College of Surgeons of England. 100(6):E150-E153. doi:10.1308/rcsann.2018.0075.

5. Chidel MA, Suh JH, Matejczyk MB (2001) Radiation prophylaxis for heterotopic ossification of the knee. Journal of Arthroplasty. 16(1):1-6. doi:10.1054/arth.2001.16492.

6. Cipriano C, Pill SG, Rosenstock J (2009) Radiation Therapy for Preventing Recurrence of Neurogenic Heterotopic Ossification. Orthopedics (Online). 32(9):685-689. doi:<https://doi.org/10.3928/01477447-20090728-33>.

7. Daugherty LC, Bell JR, Fisher BJ, Sankhla N, Tzou K, Troicki F et al (2013) Radiation prophylaxis as primary prevention of heterotopic ossification of the knee: Classification of disease and indications for treatment. Journal of Radiation Oncology. 2(1):87-94. doi:10.1007/s13566-012-0077-0.

8. Davis C, Kolovich GP, Scharschmidt TJ (2012) Atraumatic heterotopic ossification in the setting of prolonged intubation because of H1N1 influenza: a case report. Orthop Surg. 4(4):258-262. doi:10.1111/os.12009.

9. Freije SL, Kushdilian MV, Burney HN, Zang Y, Saito NG (2021) A Retrospective Analysis of 287 Patients Undergoing Prophylactic Radiation Therapy for the Prevention of Heterotopic Ossification. Adv Radiat Oncol. 6(3):100625. doi:10.1016/j.adro.2020.11.010.

10. Gibson CJ, Poduri KR (1997) Heterotopic ossification as a complication of toxic epidermal necrolysis. Archives of Physical Medicine and Rehabilitation. 78(7):774-776. doi:10.1016/s0003-9993(97)90088-5.

11. Ivey M (1985) Myositis ossificans of the thigh following manipulation of the knee. A case report. Clin Orthop Relat Res. 198):102-105.

12. Massaro M, Mela F, Esposito R, Maiorano E, Laskow G (2022) Severe Quadriceps Heterotopic Ossification after Knee Revision Arthroplasty in a 42-Year-Old Suffering from Rheumatoid Arthritis: A Case Report. Osteology. 2(4):161-165.

13. Mills WJ, Tejwani N (2003) Heterotopic ossification after knee dislocation: the predictive value of the injury severity score. J Orthop Trauma. 17(5):338-345. doi:10.1097/00005131-200305000-00004.

14. Mishra MV, Austin L, Parvizi J, Ramsey M, Showalter TN (2011) Safety and efficacy of radiation therapy as secondary prophylaxis for heterotopic ossification of non-hip joints. J Med Imaging Radiat Oncol. 55(3):333-336. doi:10.1111/j.1754-9485.2011.02275.x.

15. Rosenberg DM, Onderdonk B, Majeed NK, Guzman G, Farid Y, Connell PP et al (2019) Radiation-Induced Sarcoma After Heterotopic Ossification Prophylaxis: A Case Report. JBJS Case Connect. 9(4):e0146. doi:10.2106/jbjs.Cc.19.00146.

16. Ruiz Hernández G, Mínguez Rey MF, Gomar Sancho F, Balaguer Martínez JV, Castillo Pallarés FJ (2000) [Periarticular heterotopic ossification secondary to central neurogenic dysfunction]. Rev Esp Med Nucl. 19(7):495-499. doi:10.1016/s0212-6982(00)71919-7.

17. Shah SP, Kulshrestha A, Patel M, Mehta M, Kunikullaya S, Sharma A (2023) Radiation therapy in non-traumatic myositis ossificans of popliteal region: a case report. Journal of Radiotherapy in Practice. 22(doi:10.1017/s1460396923000341.

18. Stannard JP, Wilson TC, Sheils TM, McGwin G, Volgas DA, Alonso JE (2002) Heterotopic ossification associated with knee dislocation. Arthroscopy-the Journal of Arthroscopic and Related Surgery. 18(8):835-839. doi:10.1053/jars.2002.32842.
