# Supplementary material for: The prevention of heterotopic ossification around the knee: a scoping review
Source: BMC Musculoskelet Disord. 2026 Aug 1;27:651. doi: 10.1186/s12891-026-10318-w (PMC13428452; doi:10.1186/s12891-026-10318-w)
Supplement: Supplementary file 10 — Supplementary Material 10. [file 12891_2026_10318_MOESM10_ESM.docx]

**Supplement S10:** Treatment characteristics and outcomes of studies evaluating radiotherapy for prophylaxis of HO around the knee.

| **First author, year** | **Further details on dose and schedule** | **Timing and duration** | **Co-interventions** | **Any new HO, n/N (%)** | **Clinically relevant HO, n/N (%)** | **Knees needing further interventions for HO** | **ROM flex-ext** | **PROMs** | **Pain** | **Return to work / activity** | **Adverse events potentially related to prophylaxis** |
| --- | --- | --- | --- | --- | --- | --- | --- | --- | --- | --- | --- |
| Balen, 2001[1] | NR | Post-op | NR | NR | NR | NR | Pre-op: NR post-op: good ROM reported | NR | NR | NR | NR |
| Baroudi, 2017[2] | Single fraction, dose and schedule: NR | Post-op on day 1 | Routine rehabilitation protocol | 0/1 (0.0%) | 0/1 (0.0%) | 0/1 (0.0%) | Pre-op: 10°  post-op: 100° | NR | NR | NR | Lateral patellar dislocation, requiring surgery, secondary to medial capsular rupture after a traumatic episode, 4 weeks post-op |
| Barrack, 2002[3] | NR | NR | NR | NR | NR | NR | NR | Level of function improved | Symptoms improved | NR | NR |
| Brown, 2018[4] | 1 x 7.0 Gy | Post-op on day 1 | NR | NR | 0/1 (0.0%) | 0/1 (0.0%) | Pre-op: 20°  post-op: 80° | Patient fully satisfied with the treatment-result | NR | NR | NR |
| Chidel, 2001[5] | 1 x 7.0 Gy | Post-op on day 1 | NR | 2/4 (50.0%)§  NR: 2) | 0/6 (0.0%) | 0/6 (0.0%) | Pre-op: 35°: 1/6 (16.7%), 45°:1/6 (16.7%), full ROM 1/6 (17%), near ankylosis: 3/6 (50.0%)  post-op: Mean: 68.8° (range 35-95°): 4/6 (66.7%)†  Full ROM: 2/6 (33.3%) | NR | NR | NR | Hematoma and super infection necessitating revision TKA: 1/6 (16.7%)  None: 1/6 (16.7%)  NR: 4/6 (66.7%) |
| Cipriano, 2009[6]‡ | 1 x 7.0 Gy | Post-op, mean: 1.18 days (range: 1-4 days) after surgery | NR | IG: NR | IG: 6/37 (16.2%)* | IG: 6/37 (16.2%)* | IG: pre-op: mean: 81.3° (range: NR)*  post-op: mean: 117.5° (range: NR)* | IG: NR | IG: NR | IG: NR | IG: delayed wound healing: 12.5%*  No other AE observed* |
|  |  |  |  | CG: NR | CG: 2/35 (5.7%)* | CG: 2/35 (5.7%)* | CG: pre-op: mean: 84.2° (range: NR)*  post-op: mean: 146.7° (range: NR)* | CG: NR | CG: NR | CG: NR | CG: delayed wound healing: 12.8%*  No other AE observed |
| Daugherty, 2013[7] | 1 x 7.0 Gy | Post-op, within 72 hours after surgery | NR | 0/12 (0.0%) | 0/12 (0.0%) | 0/12 (0.0%) | Pre-op: NR  post-op: full ROM 11/12 (91.7%)  limited: 1/12 (8.3%) | NR | NR | NR | None 12/12 (100.0%) |
| Davis, 2012[8] | 1 x 7.0 Gy | Post-op on day 1 | NR | 0/1 (0.0%) | 0/1 (0.0%) | 0/1 (0.0%) | Pre-op: 100°  post-op: 85° | NR | Pre-op: yes, intensity NR  post-op: reduced intensity NR | NR | NR |
| Freije, 2021[9] | 1 x 7.0-8.0 Gy* | Pre-op: 11/287 (3.8%)*  post-op: 276/287 (96.2%)* | NR | 0/1 (0.0%) | 0/1 (0.0%) | 0/1 (0.0%) | Pre-op: NR  post-op: NR | NR | NR | NR | Acute complication (infection, sensory deficit, trochanteric bursitis): 29/287 (10.1%)*  Chronic complications (infection, sensory deficit, posttraumatic osteoarthritis trochanteric bursitis) 58/287 (20.2%)*  Acute and chronic: 12/287 (4.2%)* |
| Gibson, 1997[10] | NR | Post-op | Physical. and occupational therapy | NR | NR | NR | Pre-op: 30°  post-op: NR | NR | NR | Functional recovery to independence in activities of daily living and mobility. | NR |
| Ivey, 1985[11] | 10 x 2.0 Gy | Post-op, beginning on day 1 after surgery | NR | 0/1 (0.0%) | 0/1 (0.0%) | 0/1 (0.0%) | Pre-op: NR (before the last manipulation: 35°)  post-op: 50° | NR | NR | Patient walked with a mild limp | NR |
| Massaro, 2022[12] | 3 x 7.0 Gy | Post-op | Physical therapy, duration: 2 months | 0/1 (0.0%) | 0/1 (0.0%) | 0/1 (0.0%) | Pre-op: 55°  post-op: 90° | NR | NR | NR | NR |
| Mills, 2003[13] | NR | Post-op | NR | NR | 0/3 (0.0%) | 0/3 (0.0%) | Pre-op: mean: 0° (range: 0°)  post-op: mean: 76.7° (range: 0°-130°)† | NR | NR | NR | Wound infection with synovial-cutaneous fistula requiring knee fusion: 1/3 (33.3%) |
| Mishra, 2011[14] | 1 x 7.0 Gy | Pre-op: 1/30 (3.3%)*  post-op: 29/30 (96.7%)* | No patient was treated with additional NSAIDs. | NR | 0/7 (0.0%) | 0/7 (0.0%) | NR | NR | NR | NR | Wound infection: 2/26 (7.7%)*  Fracture in the treatment field 1/26 (3.8%)*  No case of non-union, mal-union or malignancy. |
| Rosenberg, 2019[15] | 1 x 7.0 Gy | Pre-op | NR | NR | NR | NR | NR | NR | NR | NR | Lethal radiation induced sarcoma five years after RT |
| Ruiz Hernández, 2000[16] | fractionated RT, dose and schedule: NR | Post-op | NR | NR | NR | NR | NR | NR | NR | NR | NR |
| Shah, 2023[17] | 2 x 5.0 Gy | Post-op, 2 weeks after surgery | NR | NR | 0/1 (0.0%) | 0/1 (0.0%) | NR | Pre-op: impaired mobility  post-op: no restricted mobility | Pre-op: yes, NRS 10  post-op: no, NRS 0 | NR | No acute toxicities |
| Stannard, 2002[18] | single-fraction RT, dose and schedule: NR | NR | NR | 0/3 (0.0%) | 0/3 (0.0%) | 0/3 (0.0%) | NR | NR | NR | NR | NR |

Values are reported as n/N (%) unless otherwise specified. Continuous variables are preferentially presented as mean (range). If unavailable mean ± SD or median (IQR/range) is reported according to the original publications. “Any new HO” and “clinically relevant HO” were extracted as defined in the original publications. If “clinically relevant HO” was not explicitly defined by the authors, we considered HO as clinically relevant if it was reported as symptomatic and/or required further intervention. ROM flex-ext indicates flexion–extension range of motion (degrees).

Abbreviations: AE, adverse events; CG, control group; HO, heterotopic ossification; NR, not reported; NRS, numeric rating scale; PROMs, patient-reported outcome measures; ROM, range of motion; RT, radiotherapy; TKA, total knee arthroplasty; IG, as defined by original publication.

* Values reported for the entire cohort; no separate data for the prophylaxis subgroup were provided.

† Values calculated from the reported data.

‡ Comparator-group data are shown for context where reported in the original publication.

§ HO was diagnosed in 2/6 treated and 2/4 analyzed knees, since HO status was unavailable for both knees of one patient.

**References:**

1. Balen PF, Helms CA (2001) Bony ankylosis following thermal and electrical injury. Skeletal Radiol. 30(7):393-397. doi:10.1007/s002560100342.

2. Baroudi M, Derome P, Malo M (2017) Severe heterotopic ossification and stiffness after revision knee surgery for a periprosthetic fracture. Arthroplast Today. 3(3):147-150. doi:10.1016/j.artd.2016.11.001.

3. Barrack RL, Brumfield CS, Rorabeck CH, Cleland D, Myers L (2002) Heterotopic ossification after revision total knee arthroplasty. Clinical Orthopaedics and Related Research. 404):208-213. doi:10.1097/01.blo.0000030497.43495.3f.

4. Brown A, Banerjee RD (2018) Severe heterotopic ossification following total knee replacement. Annals of the Royal College of Surgeons of England. 100(6):E150-E153. doi:10.1308/rcsann.2018.0075.

5. Chidel MA, Suh JH, Matejczyk MB (2001) Radiation prophylaxis for heterotopic ossification of the knee. Journal of Arthroplasty. 16(1):1-6. doi:10.1054/arth.2001.16492.

6. Cipriano C, Pill SG, Rosenstock J (2009) Radiation Therapy for Preventing Recurrence of Neurogenic Heterotopic Ossification. Orthopedics (Online). 32(9):685-689. doi:<https://doi.org/10.3928/01477447-20090728-33>.

7. Daugherty LC, Bell JR, Fisher BJ, Sankhla N, Tzou K, Troicki F et al (2013) Radiation prophylaxis as primary prevention of heterotopic ossification of the knee: Classification of disease and indications for treatment. Journal of Radiation Oncology. 2(1):87-94. doi:10.1007/s13566-012-0077-0.

8. Davis C, Kolovich GP, Scharschmidt TJ (2012) Atraumatic heterotopic ossification in the setting of prolonged intubation because of H1N1 influenza: a case report. Orthop Surg. 4(4):258-262. doi:10.1111/os.12009.

9. Freije SL, Kushdilian MV, Burney HN, Zang Y, Saito NG (2021) A Retrospective Analysis of 287 Patients Undergoing Prophylactic Radiation Therapy for the Prevention of Heterotopic Ossification. Adv Radiat Oncol. 6(3):100625. doi:10.1016/j.adro.2020.11.010.

10. Gibson CJ, Poduri KR (1997) Heterotopic ossification as a complication of toxic epidermal necrolysis. Archives of Physical Medicine and Rehabilitation. 78(7):774-776. doi:10.1016/s0003-9993(97)90088-5.

11. Ivey M (1985) Myositis ossificans of the thigh following manipulation of the knee. A case report. Clin Orthop Relat Res. 198):102-105.

12. Massaro M, Mela F, Esposito R, Maiorano E, Laskow G (2022) Severe Quadriceps Heterotopic Ossification after Knee Revision Arthroplasty in a 42-Year-Old Suffering from Rheumatoid Arthritis: A Case Report. Osteology. 2(4):161-165.

13. Mills WJ, Tejwani N (2003) Heterotopic ossification after knee dislocation: the predictive value of the injury severity score. J Orthop Trauma. 17(5):338-345. doi:10.1097/00005131-200305000-00004.

14. Mishra MV, Austin L, Parvizi J, Ramsey M, Showalter TN (2011) Safety and efficacy of radiation therapy as secondary prophylaxis for heterotopic ossification of non-hip joints. J Med Imaging Radiat Oncol. 55(3):333-336. doi:10.1111/j.1754-9485.2011.02275.x.

15. Rosenberg DM, Onderdonk B, Majeed NK, Guzman G, Farid Y, Connell PP et al (2019) Radiation-Induced Sarcoma After Heterotopic Ossification Prophylaxis: A Case Report. JBJS Case Connect. 9(4):e0146. doi:10.2106/jbjs.Cc.19.00146.

16. Ruiz Hernández G, Mínguez Rey MF, Gomar Sancho F, Balaguer Martínez JV, Castillo Pallarés FJ (2000) [Periarticular heterotopic ossification secondary to central neurogenic dysfunction]. Rev Esp Med Nucl. 19(7):495-499. doi:10.1016/s0212-6982(00)71919-7.

17. Shah SP, Kulshrestha A, Patel M, Mehta M, Kunikullaya S, Sharma A (2023) Radiation therapy in non-traumatic myositis ossificans of popliteal region: a case report. Journal of Radiotherapy in Practice. 22(doi:10.1017/s1460396923000341.

18. Stannard JP, Wilson TC, Sheils TM, McGwin G, Volgas DA, Alonso JE (2002) Heterotopic ossification associated with knee dislocation. Arthroscopy-the Journal of Arthroscopic and Related Surgery. 18(8):835-839. doi:10.1053/jars.2002.32842.
