# Supplementary material for: The prevention of heterotopic ossification around the knee: a scoping review
Source: BMC Musculoskelet Disord. 2026 Aug 1;27:651. doi: 10.1186/s12891-026-10318-w (PMC13428452; doi:10.1186/s12891-026-10318-w)
Supplement: Supplementary file 11 — Supplementary Material 11. [file 12891_2026_10318_MOESM11_ESM.docx]

**Supplement S11:** Study and participant characteristics of studies evaluating surgical risk factors for HO around the knee.

| **First author, year** | **Country** | **Study type** | **JBI level of evidence** | **Participants analyzed / participants enrolled** | **Index procedure / scenario** | **Indication / HO context (etiology / risk factors)** | **Follow-up (months)** | **Age (years)** | **Sex** |
| --- | --- | --- | --- | --- | --- | --- | --- | --- | --- |
| Ayhan, 2026 [1] | USA | Case control study | 3.d | 100/128 (78.1%) | Multiligament knee injury | Primary prophylaxis | Mean: 35.2 ± 34.7 | Mean: 35.5 ± 13.6 | Male 73/100 (73.0%) Female 27/100 (27.0%) |
| Gkiatas, 2021[2] | USA | Prospective cohort study | 3.c | 74/87 (85.1%) | TKA revision for stiffness or instability and aseptic loosening | Mixed: primary prophylaxis: 70/87 (80.5%) and recurrence prophylaxis: 17/87 (19.5%) | 12 months of follow-up in 74/87 (85.1%) | Mean: 67.6 (range: NR) | Male 24/87 (27.6%) Female 63/87 (72.4%) |
| Jones, 2024[3] | USA | Retrospective cohort study | 3.c | 213/213 (100.0%) | Reamed tibial nailing | Primary prophylaxis | Mean: 9.5 (range: 1.4-54.1) | Mean: 39.25 ± 15.83 | Male 171/213 (80.3%) Female 42/213 (19.7%) |

Values are reported as n/N (%) unless otherwise specified. Continuous variables are preferentially presented as mean (range). If unavailable, mean ± SD or median (IQR/range) is reported according to the original publications. “Participants analyzed / participants enrolled” denotes the number of participants included in the analysis among all enrolled participants.

Abbreviations: HO, heterotopic ossification; IQR, interquartile range; JBI, Joanna Briggs Institute; NR, not reported; SD, standard deviation; TKA, total knee arthroplasty; USA, United States of America.

**References:**

1. Ayhan EM, Levitt SJ, Nair M, Park N, Moran J, Katz L et al (2026) Heterotopic Ossification After Multiligament Knee Injury Is Associated With Knee-Spanning External Fixation and Central Nervous System Trauma. Orthopaedic Journal of Sports Medicine. 14(2):doi:10.1177/23259671261416523.

2. Gkiatas I, Xiang W, Nocon AA, Youssef MP, Tarity TD, Sculco PK (2021) Heterotopic Ossification Negatively Influences Range of Motion After Revision Total Knee Arthroplasty. Journal of Arthroplasty. 36(8):2907-2912. doi:10.1016/j.arth.2021.03.023.

3. Jones CA, Aspang JSU, Holmes JS, Zamanzadeh RS, Phen HM, Baker JL et al (2024) Incidence and Risk Factors of Heterotopic Ossification in the Knee After Reamed Tibial Nailing. Journal of the American Academy of Orthopaedic Surgeons Global Research and Reviews. 8(2):doi:10.5435/JAAOSGlobal-D-23-00258.
