# Supplementary material for: The prevention of heterotopic ossification around the knee: a scoping review
Source: BMC Musculoskelet Disord. 2026 Aug 1;27:651. doi: 10.1186/s12891-026-10318-w (PMC13428452; doi:10.1186/s12891-026-10318-w)
Supplement: Supplementary file 12 — Supplementary Material 12. [file 12891_2026_10318_MOESM12_ESM.docx]

**Supplement S12:** Treatment characteristics and outcomes of studies evaluating surgical risk factors for HO around the knee.

| **First author, year** | **Intervention** | **Co-interventions** | **Any new HO, n/N (%)** | **Clinically relevant HO, n/N (%)** | **Knees needing further interventions** | **ROM flex-ext** | **PROMs** | **Pain** | **Return to work / activity** | **Adverse events potentially related to prophylaxis** | **Further comments** |
| --- | --- | --- | --- | --- | --- | --- | --- | --- | --- | --- | --- |
| Ayhan, 2026 [1] | Risk factors were compared between patients who did and did not develop HO | Use of a hinged knee brace and physical therapy | 35/100 (35.0%) | NR | NR | NR | NR | NR | NR | NR | The use of knee-spanning external fixation (OR: 8.51) and central nervous system trauma (OR: 6.63) were both identified as independent risk factors for HO in patients with MLKI. |
| Gkiatas, 2021[2] | NSAID: used in 97%, post-op, substance, dose and schedule: NR | Physical therapy at home for 2 weeks advised, afterwards outpatient physical therapy | NR | NR | NR | Group 1: pre-op: mean: 60° ± 25°  post-op: 85° ± 28°  Group 2: pre-op: mean: 111° ± 13°  post-op: 114° ± 20° | NR | NR | NR | NR | Stiffness-revision cohort had more preop HO (30% vs 10.6%) and worse 1-year ROM (85° vs 114°); HO presence in stiffness patients linked to lower ROM despite improvement. |
|  | RT: used in 3/87 (3.4%), post-op, dose and schedule: NR |  |  |  |  |  |  |  |  |  |  |
| Jones, 2024[3] | Different surgical approaches and risk factors were compared | Additional postoperative use of ketorolac in 6 patients | 32/213 (15.0%) | NR | NR | NR | NR | NR | NR | NR | Postsurgical retroinfrapatellar reaming debris (OR: 4.73), and retrograde femoral IMN (OR: 5.08) showed a significant association with HO development. There was no significant association between HO and ketorolac use supra- or infrapatellar approach. |

Values are reported as n/N (%) unless otherwise specified. Continuous variables are preferentially presented as mean (range). If unavailable, mean ± SD or median (IQR/range) is reported according to the original publications. “Any new HO” and “clinically relevant HO” are as defined by the original publications. If “clinically relevant HO” was not explicitly defined by the authors, HO was considered clinically relevant if it was reported as symptomatic and/or required further intervention. ROM flex-ext indicates flexion–extension range of motion (degrees).

Abbreviations: AE, adverse events; HO, heterotopic ossification; IMN, intramedullary nailing; IQR, interquartile range; MLKI, multiligament knee injury; NR, not reported; NSAID, non-steroidal anti-inflammatory drug; OR, odds ratio; post-op, postoperative; pre-op, preoperative; PROMs, patient-reported outcome measures; ROM, range of motion; RT, radiotherapy; SD, standard deviation.

**References:**

1. Ayhan EM, Levitt SJ, Nair M, Park N, Moran J, Katz L et al (2026) Heterotopic Ossification After Multiligament Knee Injury Is Associated With Knee-Spanning External Fixation and Central Nervous System Trauma. Orthopaedic Journal of Sports Medicine. 14(2):doi:10.1177/23259671261416523.

2. Gkiatas I, Xiang W, Nocon AA, Youssef MP, Tarity TD, Sculco PK (2021) Heterotopic Ossification Negatively Influences Range of Motion After Revision Total Knee Arthroplasty. Journal of Arthroplasty. 36(8):2907-2912. doi:10.1016/j.arth.2021.03.023.

3. Jones CA, Aspang JSU, Holmes JS, Zamanzadeh RS, Phen HM, Baker JL et al (2024) Incidence and Risk Factors of Heterotopic Ossification in the Knee After Reamed Tibial Nailing. Journal of the American Academy of Orthopaedic Surgeons Global Research and Reviews. 8(2):doi:10.5435/JAAOSGlobal-D-23-00258.
