# Supplementary material for: The prevention of heterotopic ossification around the knee: a scoping review
Source: BMC Musculoskelet Disord. 2026 Aug 1;27:651. doi: 10.1186/s12891-026-10318-w (PMC13428452; doi:10.1186/s12891-026-10318-w)
Supplement: Supplementary file 13 — Supplementary Material 13. [file 12891_2026_10318_MOESM13_ESM.docx]

**Supplement S13:** Study and participant characteristics of studies evaluating surgical techniques for prophylaxis of HO around the knee.

| **First author, year** | **Country** | **Study type** | **JBI level of evidence** | **Participants receiving prophylaxis for HO around the knee / total enrolled** | **Knees analyzed / knees receiving prophylaxis** | **Index procedure / scenario** | **Indication / HO context (etiology / risk factors)** | **Follow-up (months)** | **Age (years)** | **Sex** |
| --- | --- | --- | --- | --- | --- | --- | --- | --- | --- | --- |
| Berven, 2018[1] | Denmark | Case-control study | 3.d | 71/154 (46.1%) | 62/71 (87.3%) | Proximal tibia fractures with a complete metaphyseal component | Primary prophylaxis | 6 | Mean: 53.0 ± 14.7† | Male 30/62 (48.4%) Female 32/62 (51.6%) |
| Bhandary, 2013[2] | India | Randomized controlled trial | 1.c | 130/285 (45.6%) | 130/130 (100.0%) | Anterior cruciate ligament reconstruction | Primary prophylaxis | 3 | Mean: 32.2 ± 7.8 | NR |
| Kent, 2018[3] | USA | Comparative cohort study | 3.c | 7/25 (28.0%) | 7/7 (100.0%) | Tibial and femoral nailing after floating knee injury | Primary prophylaxis | Mean: 16.7 ± 19.9 | Mean: 25.0 ± 9.9 | Male: 5/7 (71.4%) Female 2/7 (28.6%) |

Values are reported as n/N (%) unless otherwise specified. Continuous variables are preferentially presented as mean (range). If unavailable mean ± SD or median (IQR/range) is reported according to the original publications. “Participants receiving prophylaxis for HO around the knee/total enrolled” denotes the number of participants receiving a prophylactic intervention among all enrolled participants.

Abbreviations: HO, heterotopic ossification; JBI, Joanna Briggs Institute; NR, not reported; USA, United States of America.

† Values calculated from the reported data.

**References:**

1. Berven H, Brix M, Izadpanah K, Kubosch EJ, Schmal H (2018) Comparing case-control study for treatment of proximal tibia fractures with a complete metaphyseal component in two centers with different distinct strategies: fixation with Ilizarov frame or locking plates. Journal of Orthopaedic Surgery and Research. 13(doi:10.1186/s13018-018-0792-3.

2. Bhandary B, Shetty S, Bangera VV, R Y, Kassim MS, Alva K et al (2013) To study the incidence of heterotopic ossification after anterior cruciate ligament reconstruction. J Clin Diagn Res. 7(5):888-891. doi:10.7860/jcdr/2013/5348.2970.

3. Kent WT, Shelton TJ, Eastman J (2018) Heterotopic ossification around the knee after tibial nailing and ipsilateral antegrade and retrograde femoral nailing in the treatment of floating knee injuries. International Orthopaedics. 42(6):1379-1385. doi:10.1007/s00264-018-3845-7.
