# Supplementary material for: The prevention of heterotopic ossification around the knee: a scoping review
Source: BMC Musculoskelet Disord. 2026 Aug 1;27:651. doi: 10.1186/s12891-026-10318-w (PMC13428452; doi:10.1186/s12891-026-10318-w)
Supplement: Supplementary file 14 — Supplementary Material 14. [file 12891_2026_10318_MOESM14_ESM.docx]

**Supplement S14:** Treatment characteristics and outcomes of studies evaluating surgical approaches for prophylaxis of HO around the knee.

| **First author, year** | **Intervention** | **Co-interventions** | **Any new HO, n/N (%)** | **Clinically relevant HO, n/N (%)** | **Knees needing further interventions** | **ROM flex-ext** | **PROMs** | **Pain** | **Return to work / activity** | **Adverse events potentially related to prophylaxis** | **Further comments** |
| --- | --- | --- | --- | --- | --- | --- | --- | --- | --- | --- | --- |
| Berven, 2018[1] | Group A: external fixation | NR | Group A: 1/62 (1.6%) | NR | Group A: reoperation: 8/62 (12.9%) | Group A: postop median: 107.5 ± 19° | NR | NR | NR | Higher rate of superficial infections (40.4% vs 2.9%, p = 0.000) and longer time of healing (p = 0.041) for external fixation. | Significantly higher HO prevalence after plate fixation (p = 0.013)  No difference regarding deep infections (p = 0.634), reoperation rate (p = 0.766), peroneal nerve paresis (p = 0.106), and deep venous thrombosis (p = 0.465). |
|  | Group B: plate fixation‡ | NR | Group B: 9/68 (13.2%) | NR | Group B: reoperation: 10/68 (14.7%) | Group B: postop median: 117.5 ± 20.9° | NR | NR | NR |  |  |
| Bhandary, 2013[2] | Group A: copious lavage of the femoral canal following reaming, hemostasis, and nibbling of excessive femoral bone plug | Physical therapy | Group A: 2/130 (1.5%) | NR | NR | NR | NR | NR | NR | NR | The authors report a significant reduction of HO incidence in Group A due to preventive measures. However, there are no information regarding the statistical tests used for the analysis. |
|  | Group B: no measures to prevent HO‡ | Physical therapy | Group B: 4/155 (2.6%) | NR | NR | NR | NR | NR | NR | NR |  |
| Kent, 2018[3] | Group A: antegrade femoral nailing | NR | Group A: 3/7 (42.9%) | Group A: 0/7 (0.0%) | NR | Group A: postop mean: 128 ± 14°† | NR | NR | NR | NR | Significantly higher HO prevalence (p = 0.028) and severity (p = 0.004) in group B. No significant difference regarding knee ROM (p = 0.439) between both groups. |
|  | Group B: retrograde femoral nailing‡ | NR | Group B: 17/19 (89.5%)§ | Group B: 1/19 (5.3%)§ | NR | Group B: postop mean: 121 ± 16°† | NR | NR | NR | NR |  |

Values are reported as n/N (%) unless otherwise specified. Continuous variables are preferentially presented as mean (range). If unavailable mean ± SD or median (IQR/range) is reported according to the original publications. “Any new HO” and “clinically relevant HO” were extracted as defined in the original publications. If “clinically relevant HO” was not explicitly defined by the authors, we considered HO as clinically relevant if it was reported as symptomatic and/or required further intervention. ROM flex-ext indicates flexion–extension range of motion (degrees).

Abbreviations: HO, heterotopic ossification; NR, not reported; OR, odds ratio; PROMs, patient-reported outcome measures; ROM, range of motion.

† Values calculated from the reported data.

‡ Comparator-group data are shown for context where reported in the original publication.

§ 19 knees in 18 patients

**References:**

1. Berven H, Brix M, Izadpanah K, Kubosch EJ, Schmal H (2018) Comparing case-control study for treatment of proximal tibia fractures with a complete metaphyseal component in two centers with different distinct strategies: fixation with Ilizarov frame or locking plates. Journal of Orthopaedic Surgery and Research. 13(doi:10.1186/s13018-018-0792-3.

2. Bhandary B, Shetty S, Bangera VV, R Y, Kassim MS, Alva K et al (2013) To study the incidence of heterotopic ossification after anterior cruciate ligament reconstruction. J Clin Diagn Res. 7(5):888-891. doi:10.7860/jcdr/2013/5348.2970.

3. Kent WT, Shelton TJ, Eastman J (2018) Heterotopic ossification around the knee after tibial nailing and ipsilateral antegrade and retrograde femoral nailing in the treatment of floating knee injuries. International Orthopaedics. 42(6):1379-1385. doi:10.1007/s00264-018-3845-7.
