# Supplementary material for: The prevention of heterotopic ossification around the knee: a scoping review
Source: BMC Musculoskelet Disord. 2026 Aug 1;27:651. doi: 10.1186/s12891-026-10318-w (PMC13428452; doi:10.1186/s12891-026-10318-w)
Supplement: Supplementary file 15 — Supplementary Material 15. [file 12891_2026_10318_MOESM15_ESM.docx]

**Supplement S15:** Study and participant characteristics of studies evaluating combined prophylaxis strategies for HO around the knee.

| **First author, year** | **Country** | **Study type** | **JBI level of evidence** | **Participants receiving prophylaxis for HO around the knee / total enrolled** | **Knees analyzed / knees receiving prophylaxis** | **Index procedure / scenario** | **Indication / HO context (etiology / risk factors)** | **Follow-up (months)** | **Age (years)** | **Sex** |
| --- | --- | --- | --- | --- | --- | --- | --- | --- | --- | --- |
| Anderson, 2004[1] | USA | Case report | 4.d | 1/1 (100.0%) | 1/1 (100.0%) | Removal of neurogenic HO | Recurrence prophylaxis | 7 | 20 | Male 1/1 (100.0%) |
| Camillieri, 2013[2] | Italy | Case report | 4.d | 1/1 (100.0%) | 1/1 (100.0%) | Removal of HO after ACL reconstruction | Recurrence prophylaxis | 36 | 42 | Male 1/1 (100.0%) |
| Choi, 2022[3] | USA | Case report | 4.d | 1/1 (100.0%) | 1/1 (100.0%) | Primary TKA | Recurrent HO around the contralateral knee resulting in complete ankylosis | 12 | 63 | Female 1/1 (100.0%) |
| Espandar, 2010[4] | Iran | Case report | 4.d | 1/1 (100.0%) | 2/2 (100.0%) | Removal of neurogenic HO | Recurrence prophylaxis | 6 | 39 | Male 1/1 (100.0%) |
| Estel, 2024[5] | USA | Case report | 4.d | 1/1 (100.0%) | 1/1 (100.0%) | Removal of neurogenic HO | Recurrence prophylaxis | 48 | 31 | Male 1/1 (100.0%) |
| Iida, 2021[6] | Japan | Case report | 4.d | 1/1 (100.0%) | 2/2 (100.0%) | Removal of neurogenic HO | Recurrence prophylaxis | 36 | 31 | Female 1/1 (100.0%) |
| Jacobs, 1999[7] | Netherlands | Case report | 4.d | 1/1 (100.0%) | 2/2 (100.0%) | Removal of HO caused by an episode of critical illness due to pancreatitis | Recurrence prophylaxis | 12 | 52 | Female 1/1 (100.0%) |
| Karthik, 2025[8] | India | Case report | 4.d | 1/1 (100.0%) | 1/1 (100.0%) | Removal of HO after TKA | Recurrence prophylaxis | 8 | 67 | Female 1/1 (100.0%) |
| Kerdoncuff, 2002[9] | France | Case series | 4.c | 1/3 (33.3%) | 1/1 (100.0%) | Removal of neurogenic HO | Recurrence prophylaxis | 14 | 49 | Female 1/1 (100.0%) |
| Kolessar, 1996[10] | USA | Case series | 4.c | 3/17 (17.6%) | 3/3 (100.0%) | Removal of neurogenic HO | Recurrence prophylaxis | Mean: 22.3 (range: 18-26) | Mean: 35 (range: 24-55)* | Male 14/17 (82.4%)*  Female 3/17 (17.6%)* |
| Papadopoulos, 2004[11] | Greece | Case report | 4.d | 1/1 (100.0%) | 1/1 (100.0%) | Removal of HO after previous internal fixation of a Hoffa fracture | Recurrence prophylaxis | 24 | 39 | Male 1/1 (100.0%) |
| Sugita, 2005[12] | Japan | Case report | 4.d | 1/1 (100.0%) | 2/2 (100.0%) | Removal of neurogenic HO | Recurrence prophylaxis | 54 | 42 | Male 1/1 (100.0%) |
| Takemoto, 2011[13] | USA | Case report | 4.d | 1/1 (100.0%) | 1/1 (100.0%) | Removal of post-traumatic HO | Recurrence prophylaxis | 1 | 32 | Male 1/1 (100.0%) |
| Thienpont, 2006[14] | Belgium | Case report | 4.d | 1/1 (100.0%) | 1/1 (100.0%) | Removal of HO after TKA | Recurrence prophylaxis | 24 | 58 | Male 1/1 (100.0%) |

Values are reported as n/N (%) unless otherwise specified. Continuous variables are preferentially presented as mean (range). If unavailable mean ± SD or median (IQR/range) is reported according to the original publications. “Participants receiving prophylaxis for HO around the knee / total enrolled” denotes the number of participants receiving the prophylaxis modality among all enrolled participants. “Knees analyzed / knees receiving prophylaxis” denotes the number of knees included in the analysis among knees receiving prophylaxis (if reported).

Abbreviations: ACL, anterior cruciate ligament; HO, heterotopic ossification; JBI, Joanna Briggs Institute; TKA, total knee arthroplasty; USA, United States of America.

* Values reported for the entire cohort; no separate data for the prophylaxis subgroup were provided.

**References:**

1. Anderson MC, Lais RL (2004) Excision of heterotopic ossification of the popliteal space following traumatic brain injury. Journal of Orthopaedic Trauma. 18(3):190-192. doi:10.1097/00005131-200403000-00013.

2. Camillieri G, Di Sanzo V, Ferretti M, Calderaro C, Calvisi V (2013) Patellar tendon ossification after anterior cruciate ligament reconstruction using bone - patellar tendon - bone autograft. Bmc Musculoskeletal Disorders. 14(doi:10.1186/1471-2474-14-164.

3. Choi JH, Levens B, Fox J, Kamara E (2022) Successful Total Knee Arthroplasty in a Patient With Contralateral Ankylosis Due to Severe Heterotopic Ossification. Cureus. 14(5):e24941. doi:10.7759/cureus.24941.

4. Espandar R, Haghpanah B (2010) Acceptable outcome following resection of bilateral large popliteal space heterotopic ossification masses in a spinal cord injured patient: a case report. Journal of Orthopaedic Surgery and Research. 5(doi:10.1186/1749-799x-5-39.

5. Estel K, Back DA, Scheuermann-Poley C, Willy C (2024) Fulminant Heterotopic Ossification of the Lower Extremity After Gunshot Injury and Blunt Trauma: A Case Report. Military Medicine. 189(7-8):e1826-e1831. doi:10.1093/milmed/usae109.

6. Iida K, Hashimoto Y, Okazaki S, Nishida Y, Nakamura H (2021) Surgical excision of heterotopic ossification associated with anti-N-methyl-d-aspartate receptor encephalitis: A case report. Int J Surg Case Rep. 89(106643. doi:10.1016/j.ijscr.2021.106643.

7. Jacobs JWG, De Sonnaville PBJ, Hulsmans HMJ, Van Rinsum AC, Bijlsma JWJ (1999) Polyarticular heterotopic ossification complicating critical illness. Rheumatology. 38(11):1145-1149. doi:10.1093/rheumatology/38.11.1145.

8. Karthik MS, Mohammed A, Parthasarathy A (2025) Rare Case of Heterotopic Ossification Impinging on the Quadriceps Mechanism Following Total Knee Replacement: A Case Report. J Orthop Case Rep. 15(9):83-87. doi:10.13107/jocr.2025.v15.i09.6024.

9. Kerdoncuff V, Sauleau P, Petrilli S, Duruflé A, Ben Beroukh K, Brissot R et al (2002) Heterotopic ossification in Guillain-Barré syndrome. Annales de Readaptation et de Medecine Physique. 45(5):198-203. doi:10.1016/S0168-6054(02)00203-9.

10. Kolessar DJ, Katz SD, Keenan MA (1996) Functional outcome following surgical resection of heterotopic ossification in patients with brain injury. Journal of Head Trauma Rehabilitation. 11(4):78-87. doi:10.1097/00001199-199608000-00010.

11. Papadopoulos AX, Panagopoulos A, Karageorgos A, Tyllianakis M (2004) Operative treatment of unilateral bicondylar Hoffa fractures. Journal of Orthopaedic Trauma. 18(2):119-122. doi:10.1097/00005131-200402000-00012.

12. Sugita A, Hashimoto J, Maeda A, Kobayashi J, Hirao M, Masuhara K et al (2005) Heterotopic ossification in bilateral knee and hip joints after long-term sedation. Journal of Bone and Mineral Metabolism. 23(4):329-332. doi:10.1007/s00774-005-0608-5.

13. Takemoto RC, Epstein D, McLaurin TM (2011) Intra- and Periarticular Heterotopic Ossification in the Knee After a Low-Velocity Gunshot Wound Treated With Retrograde Intramedullary Nailing of the Femur. Journal of Orthopaedic Trauma. 25(7):E77-E82. doi:10.1097/BOT.0b013e3181f981e1.

14. Thienpont E, Schmalzried T, Bellemans J (2006) Ankylosis due to heterotopic ossification following primary total knee arthroplasty. Acta Orthop Belg. 72(4):502-506.
