# Supplementary material for: The prevention of heterotopic ossification around the knee: a scoping review
Source: BMC Musculoskelet Disord. 2026 Aug 1;27:651. doi: 10.1186/s12891-026-10318-w (PMC13428452; doi:10.1186/s12891-026-10318-w)
Supplement: Supplementary file 16 — Supplementary Material 16. [file 12891_2026_10318_MOESM16_ESM.docx]

**Supplement S16:** Treatment characteristics and outcomes of studies evaluating combined prophylaxis strategies for HO around the knee.

| **First author, year** | **Inter-vention for HO prophy-laxis** | **Further details on dose and schedule** | **Timing and duration** | **Co-inter-ventions** | **Any new HO, n/N (%)** | **Clinically relevant HO, n/N (%)** | **Knees needing further inter-ventions for HO** | **ROM flex-ext** | **PROMs** | **Pain** | **Return to work / activity** | **Adverse events potentially related to prophylaxis** |
| --- | --- | --- | --- | --- | --- | --- | --- | --- | --- | --- | --- | --- |
| Anderson, 2004[1] | CPM | NR | From d1 to d3 after surgery | Outpatient physical therapy, 3 times weekly for 4.5 months | 0/1 (0.0%) | 0/1 (0.0%) | 0/1 (0.0%) | Pre-op: 0°  post-op: 115° | NR | NR | Ambulating without any assistive device | NR |
|  | NSAID | Indomethacin 75 mg daily | Post-op for 6 weeks |  |  |  |  |  |  |  |  |  |
|  | RT | 1 x 8.0 Gy | Delivered on d1 after surgery |  |  |  |  |  |  |  |  |  |
| Camillieri, 2013[2] | CPM | Flexion 0-90° TID | Post-op duration: 6 weeks | Initially: rigid orthosis locked in extension  physical therapy for 6 weeks | 0/1 (0.0%) | 0/1 (0.0%) | 0/1 (0.0%) | Pre-op: 110°  post-op: no limitation reported | Pre-op: IKDC score: 41.1  post-op: IKDC score: 90.8 | Pre-op: yes, intensity NR  post-op: none. | Return to normal activity after 6 weeks | NR |
|  | NSAID | Ibuprofen 200 mg TID | Post-op duration: 1 week |  |  |  |  |  |  |  |  |  |
| Choi, 2022[3] | NSAID | Indomethacin 25 mg TID | Post-op for 3 months | NR | 0/1 (0.0%) | 0/1 (0.0%) | 0/1 (0.0%) | Pre-op: 110°  post-op: 110° | NR | Pre-op: yes, intensity NR  post-op: reduced | NR | NR |
|  | RT | 1 x 7.0 Gy | Pre-op 1 day before TKA |  |  |  |  |  |  |  |  |  |
|  | Bisphos-phonates | Etidronate 400mg QD | Started 1 month post-op for 3 months |  |  |  |  |  |  |  |  |  |
| Espandar, 2010[4] | NSAID | Indomethacin 75 mg daily | Post-op for 6 weeks | Hinged knee brace in 60° flexion for two days, afterwards passive ROM exercises | NR | 0/2 (0.0%) | 0/2 (0.0%) | Pre-op: 0°, 0°  post-op: 80°, 75° | NR | NR | NR | Deep vein thrombosis of the calf |
|  | RT | 1 x 7.0 Gy | Delivered on day 1 after surgery |  |  |  |  |  |  |  |  |  |
| Estel, 2024[5] | NSAID | Indomethacin 25 mg TID | Post-op for 6 weeks | Intensive pain therapy to allow early functional physio-therapeutic exercise | NR | 0/1 (0.0%) | 0/1 (0.0%) | Pre-op: 0°  post-op: 50° | NR | Pre-op: NR  post-op: largely pain-free | Recovered ability to walk upright without walking aid | NR |
|  | RT | 1 x 7.0 Gy | Perioperative |  |  |  |  |  |  |  |  |  |
| Iida, 2021[6] | NSAID | Loxoprofen 180 mg once daily | Post-operative for 12 weeks | NR | 0/2 (0.0%) | 0/2 (0.0%) | 0/2 (0.0%) | Pre-op: 30°, 40°  post-op: 130°, 130° | NR | Pre-op: yes, intensity NR  post-op: none | Recovered the ability to walk independently | NR |
|  | Bisphos-phonates | Etidronate 1000mg once daily | Post-operative for 12 weeks |  |  |  |  |  |  |  |  |  |
| Jacobs, 1999[7] | NSAID | Drug, dose and schedule NR | Post-operative for 6 weeks | NR | 0/1 (0.0%) | 0/1 (0.0%) | 0/1 (0.0%) | Pre-op: 0°, 10°  post-op: 90°, 100° | NR | Pre-op: yes, intensity NR  post-op: NR | Ability to walk improved | NR |
|  | Bisphos-phonates | Drug, dose and schedule NR | Post-operative for 6 weeks |  |  |  |  |  |  |  |  |  |
| Karthik, 2025[8] | NSAID | Indomethacin 75 mg once daily | Post-operative for 3 months | NR | 0/1 (0.0%) | 0/1 (0.0%) | 0/1 (0.0%) | Pre-op: 50°  post-op: 80° | Post-op: symptoms improved | Pre-op: yes, occasio-nally  post-op: NR | NR | Wound healed uneventfully. |
|  | RT | 1 x 0.75 Gy | Post-op |  |  |  |  |  |  |  |  |  |
| Kerdoncuff, 2002[9] | NSAID | Indomethacin, dose and schedule NR | NR | NR | NR | NR | NR | Pre-op: reduced  post-op: NR | NR | Pre-op: yes, intensity: NR  post-op: NR | Recovered ability to climb stairs and walk a short distance | NR |
|  | Bisphos-phonates | Etidronate, dose and schedule NR | Started 1.5 months pre-op, duration: NR |  |  |  |  |  |  |  |  |  |
| Kolessar, 1996[10] | NSAID | Indomethacin at least 75 mg per day | Timing: NR, duration: typically 3 months | Physical therapy | 5/21 (23.8%)* | 1/21 (4.8%)* | 0/3 (0.0%) | Pre-op: mean: 47° (range: 20-95°)  post-op: mean: 98° (range: 85-125°) | NR | Improved pain post-op: 1/3 (33.3%)  NR: 2/3 (66.7%) | Failure to improve ambulation: 1/3 (33.3%)  Achieved improved sitting in a wheelchair: 1/3 (33.3%)  NR: 1/3 (33.3%) | Adverse events that potentially occurred in knee patients: Nerve palsy 1/21 (4.8%)*  Infection requiring debridement 1/21 (4.8%)* |
|  | Bisphos-phonates | Etidronate 20 mg/kg per day | Timing: NR, duration: typically 3 months |  |  |  |  |  |  |  |  |  |
| Papadopoulos, 2004[11] | CPM | NR | NR | NR | NR | 0/1 (0.0%) | 0/1 (0.0%) | Pre-op: 80°  post-op: 145° | No complaints noted | None | Regained all his daily and working abilities | NR |
|  | NSAID | Indomethacin 75 mg, once daily | Post-op, duration: 2 weeks |  |  |  |  |  |  |  |  |  |
| Sugita, 2005[12] | NSAID | Indomethacin 50 mg per day | Started pre-op 7 months before surgery, continued for 6 months post-op | Physical therapy with ROM exercises, from day 1 post-op, duration: 6 months | 0/2 (0.0%) | 0/2 (0.0%) | 0/2 (0.0%) | Pre-op: 20°, 20°  post-op: 115°, 100° | NR | Pre-op: none  post-op: NR | Resumed office work 6 months after HO resection. | NR |
|  | Bisphos-phonates | Etidronate 1000 mg per day | Started pre-op 7 months before surgery, continued for 6 months post-op |  |  |  |  |  |  |  |  |  |
| Takemoto, 2011[13] | CPM | NR | Started immediately after surgery, used for 3d | NR | 0/1 (0.0%) | 0/1 (0.0%) | 0/1 (0.0%) | Pre-op: 75°  post-op: 95° | NR | NR | NR | None |
|  | NSAID | Indomethacin 75 mg daily | Post-operative for 6 weeks | NR |  |  |  |  |  |  |  |  |
| Thienpont, 2006[14] | NSAID | Indomethacin, dose and schedule: NR | Post-op, duration: 6 weeks | NR | 0/1 (0.0%) | 0/1 (0.0%) | 0/1 (0.0%) | Pre-op: 0°  post-op: 115° | NR | NR | NR | NR |
|  | RT | 1 x 7.0 Gy | Pre-op |  |  |  |  |  |  |  |  |  |

Values are reported as n/N (%) unless otherwise specified. Continuous variables are preferentially presented as mean (range). If unavailable mean ± SD or median (IQR/range) is reported according to the original publications. “Any new HO” and “clinically relevant HO” were extracted as defined in the original publications. If “clinically relevant HO” was not explicitly defined by the authors, we considered HO as clinically relevant if it was reported as symptomatic and/or required further intervention. ROM flex-ext indicates flexion–extension range of motion (degrees).

Abbreviations: CPM, continuous passive motion; HO, heterotopic ossification; IKDC, International Knee Documentation Committee score; NR, not reported; NSAID, non-steroidal anti-inflammatory drug; PROMs, patient-reported outcome measures; QD, once daily; ROM, range of motion; RT, radiotherapy; TID, three times daily; TKA, total knee arthroplasty.

* Values reported for the entire cohort; no separate data for the prophylaxis subgroup were provided.

**References:**

1. Anderson MC, Lais RL (2004) Excision of heterotopic ossification of the popliteal space following traumatic brain injury. Journal of Orthopaedic Trauma. 18(3):190-192. doi:10.1097/00005131-200403000-00013.

2. Camillieri G, Di Sanzo V, Ferretti M, Calderaro C, Calvisi V (2013) Patellar tendon ossification after anterior cruciate ligament reconstruction using bone - patellar tendon - bone autograft. Bmc Musculoskeletal Disorders. 14(doi:10.1186/1471-2474-14-164.

3. Choi JH, Levens B, Fox J, Kamara E (2022) Successful Total Knee Arthroplasty in a Patient With Contralateral Ankylosis Due to Severe Heterotopic Ossification. Cureus. 14(5):e24941. doi:10.7759/cureus.24941.

4. Espandar R, Haghpanah B (2010) Acceptable outcome following resection of bilateral large popliteal space heterotopic ossification masses in a spinal cord injured patient: a case report. Journal of Orthopaedic Surgery and Research. 5(doi:10.1186/1749-799x-5-39.

5. Estel K, Back DA, Scheuermann-Poley C, Willy C (2024) Fulminant Heterotopic Ossification of the Lower Extremity After Gunshot Injury and Blunt Trauma: A Case Report. Military Medicine. 189(7-8):e1826-e1831. doi:10.1093/milmed/usae109.

6. Iida K, Hashimoto Y, Okazaki S, Nishida Y, Nakamura H (2021) Surgical excision of heterotopic ossification associated with anti-N-methyl-d-aspartate receptor encephalitis: A case report. Int J Surg Case Rep. 89(106643. doi:10.1016/j.ijscr.2021.106643.

7. Jacobs JWG, De Sonnaville PBJ, Hulsmans HMJ, Van Rinsum AC, Bijlsma JWJ (1999) Polyarticular heterotopic ossification complicating critical illness. Rheumatology. 38(11):1145-1149. doi:10.1093/rheumatology/38.11.1145.

8. Karthik MS, Mohammed A, Parthasarathy A (2025) Rare Case of Heterotopic Ossification Impinging on the Quadriceps Mechanism Following Total Knee Replacement: A Case Report. J Orthop Case Rep. 15(9):83-87. doi:10.13107/jocr.2025.v15.i09.6024.

9. Kerdoncuff V, Sauleau P, Petrilli S, Duruflé A, Ben Beroukh K, Brissot R et al (2002) Heterotopic ossification in Guillain-Barré syndrome. Annales de Readaptation et de Medecine Physique. 45(5):198-203. doi:10.1016/S0168-6054(02)00203-9.

10. Kolessar DJ, Katz SD, Keenan MA (1996) Functional outcome following surgical resection of heterotopic ossification in patients with brain injury. Journal of Head Trauma Rehabilitation. 11(4):78-87. doi:10.1097/00001199-199608000-00010.

11. Papadopoulos AX, Panagopoulos A, Karageorgos A, Tyllianakis M (2004) Operative treatment of unilateral bicondylar Hoffa fractures. Journal of Orthopaedic Trauma. 18(2):119-122. doi:10.1097/00005131-200402000-00012.

12. Sugita A, Hashimoto J, Maeda A, Kobayashi J, Hirao M, Masuhara K et al (2005) Heterotopic ossification in bilateral knee and hip joints after long-term sedation. Journal of Bone and Mineral Metabolism. 23(4):329-332. doi:10.1007/s00774-005-0608-5.

13. Takemoto RC, Epstein D, McLaurin TM (2011) Intra- and Periarticular Heterotopic Ossification in the Knee After a Low-Velocity Gunshot Wound Treated With Retrograde Intramedullary Nailing of the Femur. Journal of Orthopaedic Trauma. 25(7):E77-E82. doi:10.1097/BOT.0b013e3181f981e1.

14. Thienpont E, Schmalzried T, Bellemans J (2006) Ankylosis due to heterotopic ossification following primary total knee arthroplasty. Acta Orthop Belg. 72(4):502-506.
