# Supplementary material for: The prevention of heterotopic ossification around the knee: a scoping review
Source: BMC Musculoskelet Disord. 2026 Aug 1;27:651. doi: 10.1186/s12891-026-10318-w (PMC13428452; doi:10.1186/s12891-026-10318-w)
Supplement: Supplementary file 17 — Supplementary Material 17. [file 12891_2026_10318_MOESM17_ESM.docx]

**Supplement S17.** Risk of bias assessment of the studies

| **First author, year** | **General assessment and inclusion (yes/no/unclear)** | **Limits in the following domains** | **Overall rating (Yes, No, Unclear / (Total - NA))** | **JBI level of evidence** | **Reported conflicts of interest and funding** | **Notable concerns due to conflicts of interest or funding** |
| --- | --- | --- | --- | --- | --- | --- |
| **Randomized controlled trials** | | | | | |  |
| Bhandary,  2013 [1] | Yes | Randomization process and allocation concealment unclear; treating surgeons could not be blinded; participant and outcome-assessor blinding unclear; completeness of follow-up unclear; statistical analysis insufficiently reported, with unsupported claims of significance. | Yes 6/13, No 2/13, Unclear 5/13 | 1.c | None reported | No |
| **Cohort studies** | | | | | |  |
| Kent, 2018 [2] | Yes | Confounding factors were identified, but not adjusted for; patients with insufficient follow-up were excluded from analysis; no strategies employed to address incomplete follow-up | Yes 8/11, No 3/11, Unclear 0/11 | 3.c | None reported | No |
| Van Nest, 2021 [3] | Yes | Patients with insufficient follow-up were excluded from analysis; no strategies employed to address incomplete follow-up | Yes 9/11, No 2/11, Unclear 0/11 | 3.c | One author disclosed royalties from Corentec; paid consultancy for multiple medical-device and biotechnology companies; stock or stock options in several companies; and royalties or other support from multiple publishers. The other authors report no conflicts of interest. | No |
| Stannard, 2002 [4] | Yes | Confounding factors were identified, but not adjusted for; completeness of follow-up unclear; no strategies employed to address incomplete follow-up | Yes 8/11, No 2/11, Unclear 1/11 | 3.e | NR | - |
| **Case-control studies** | | | | | |  |
| Berven,  2018 [5] | Yes | No matching procedure was performed; Overall: relevant risk for confounding and selection bias due to non-existent matching | Yes 9/10, No 1/10, Unclear 0/10 | 3.d | None reported | No |
| Cipriano,  2009 [6] | Yes | Relevant differences between intervention and control group regarding recurrence risk, resulting in major confounding by indication for effectiveness of RT; no sufficient explanation of statistical tests used; regarding AEs: between-group differences might increase risk of AE in the intervention group slightly; Overall: high risk for confounding and selection bias due to non-existent matching and relevant differences between both groups | Yes 6/10, No 3/10, Unclear 1/10 | 3.d | None reported | No |
| **Case series** | | | | | | |
| Abdelfettah, 2012 [7] | Yes | Abstract only, consecutive and complete inclusion unclear, method for identification unclear | Yes 6/9, No 0/9, Unclear 3/9 | 4.c | NR | - |
| Barrack, 2002 [8] | Yes | No complete inclusion of patients with 16/151 patients missing from follow-up | Yes 9/10, No 1/10, Unclear 0/10 | 4.c | NR | - |
| Belhaj, 2013 [9] | Yes | Abstract only, consecutive and complete inclusion unclear, unclear, if condition was measured in a standard, reliable way, reporting sites' demographics unclear | Yes 5/9, No 0/9, Unclear 4/9 | 4.c | NR | - |
| Charnley, 1996 [10] | Yes | Complete inclusion unclear, reporting sites' demographics unclear | Yes 7/9, No 0/9, Unclear 2/9 | 4.c | NR | - |
| Chidel, 2001 [11] | Yes | Consecutive and complete inclusion unclear, reporting sites' demographics unclear | Yes 6/9, No 0/9, Unclear 3/9 | 4.c | None reported | No |
| Dalury, 2004 [12] | Yes | - | Yes 9/9, No 0/9, Unclear 0/9 | 4.c | None reported | No |
| Daugherty, 2013 [13] | Yes | - | Yes 9/9, No 0/9, Unclear 0/9 | 4.c | None reported | No |
| Freije, 2021 [14] | Yes | - | Yes 10/10, No 0/10, Unclear 0/10 | 4.c | None reported | No |
| Fuller, 2005 [15] | Yes | - | Yes 10/10, No 0/10, Unclear 0/10 | 4.c | None reported | No |
| Ippolito, 1999 [16] | Yes | - | Yes 9/9, No 0/9, Unclear 0/9 | 4.c | None reported | No |
| Kerdoncuff, 2002 [17] | Yes | Complete and consecutive inclusion unclear, reporting sites' demographics unclear | Yes 6/9, No 0/9, Unclear 3/9 | 4.c | NR | - |
| Kolessar, 1996 [18] | Yes | - | Yes 9/9, No 0/9, Unclear 0/9 | 4.c | NR | - |
| Mills, 2003 [19] | Yes | Complete inclusion unclear | Yes 9/10, No 0/10, Unclear 1/10 | 4.c | NR | - |
| Mishra, 2011 [20] | Yes | Complete and consecutive inclusion unclear; information regarding any HO unclear | Yes 6/9, No 0/9, Unclear 3/9 | 4.c | None reported | No |
| Mitsionis, 2009 [21] | Yes | - | Yes 9/9, No 0/9, Unclear 0/9 | 4.c | NR | - |
| Ogilvie-Harris, 1995 [22] | Yes | Complete and consecutive inclusion unclear, patients' demographic information not available in sufficient detail, reporting sites' demographics unclear | Yes 5/9, No 1/9, Unclear 3/9 | 4.c | NR | - |
| Parvizi, 2001 [23] | Yes | - | Yes 10/10, No 0/10, Unclear 0/10 | 4.c | None reported | No |
| Rader, 1997 [24] | Yes | Complete and consecutive inclusion unclear, use of the F-test for ordinal HO grades not justified | Yes 7/10, No 0/10, Unclear 3/10 | 4.c | NR | - |
| Subbarao, 1987 [25] | Yes | - | Yes 9/9, No 0/9, Unclear 0/9 | 4.c | NR | - |
| **Case reports** | | | | | | |
| Alturki, 2020 [26] | Yes | Takeaway lesson unclear | Yes 7/8, No 0/8, Unclear 1/8 | 4.d | None reported | No |
| Anderson, 2004 [27] | Yes | Takeaway lesson unclear | Yes 7/8, No 0/8, Unclear 1/8 | 4.d | None reported | No |
| Balen, 2001 [28] | Yes | Intervention details unclear | Yes 7/8, No 0/8, Unclear 1/8 | 4.d | NR | - |
| Baroudi, 2017 [29] | Yes | - | Yes 8/8, No 0/8, Unclear 0/8 | 4.d | None reported | No |
| Bellemans, 1999 [30] | Yes | - | Yes 8/8, No 0/8, Unclear 0/8 | 4.d | NR | - |
| Bragg, 2022 [31] | Yes | - | Yes 8/8, No 0/8, Unclear 0/8 | 4.d | None reported | No |
| Brown, 2018 [32] | Yes | - | Yes 8/8, No 0/8, Unclear 0/8 | 4.d | NR | - |
| Camillieri, 2013 [33] | Yes | Unclear if any adverse events occurred | Yes 7/8, No 0/8, Unclear 1/8 | 4.d | None reported | No |
| Cho, 2011 [34] | Yes | - | Yes 8/8, No 0/8, Unclear 0/8 | 4.d | None reported | No |
| Choi, 2022 [35] | Yes | - | Yes 8/8, No 0/8, Unclear 0/8 | 4.d | None reported | No |
| Daniilidis, 2013 [36] | Yes | Patient's history and timeline remain unclear due to a lack of details | Yes 7/8, No 0/8, Unclear 1/8 | 4.d | None reported | No |
| Davis, 2012 [37] | Yes | Unclear if any adverse events occurred | Yes 7/8, No 0/8, Unclear 1/8 | 4.d | None reported | No |
| Erdil, 2012 [38] | Yes | - | Yes 8/8, No 0/8, Unclear 0/8 | 4.d | None reported | No |
| Espandar, 2010 [39] | Yes | - | Yes 8/8, No 0/8, Unclear 0/8 | 4.d | None reported | No |
| Estel, 2024 [40] | Yes | - | Yes 8/8, No 0/8, Unclear 0/8 | 4.d | None reported | No |
| Firoozabadi, 2025 [41] | Yes | - | Yes 8/8, No 0/8, Unclear 0/8 | 4.d | None reported | No |
| Gibson, 1997 [42] | Yes | Post-intervention clinical condition due to a lack of details | Yes 7/8, No 0/8, Unclear 1/8 | 4.d | None reported | No |
| Hari Krishnan, 2016 [43] | Yes | Adverse events of initial treatment clearly reported, but occurrence of adverse events after HO removal remains unclear | Yes 7/8, No 0/8, Unclear 1/8 | 4.d | None reported | No |
| Hoffer, 2024 [44] | Yes | - | Yes 8/8, No 0/8, Unclear 0/8 | 4.d | The authors declare that there are no financial conflicts or competing interests regarding publication of this article. Dr. Lyons is an Academic Editor for Case Reports in Orthopedics. | No |
| Iida, 2021 [45] | Yes | - | Yes 8/8, No 0/8, Unclear 0/8 | 4.d | None reported | No |
| Ivey, 1985 [46] | Yes | Adverse events of initial treatment clearly reported, but occurrence of adverse events after HO removal remains unclear | Yes 7/8, No 0/8, Unclear 1/8 | 4.d | NR | - |
| Jacobs, 1999 [47] | Yes | Intervention details, especially medication dose and duration remain unclear, Treatment results unclear due to lack of details, Occurrence of adverse events after HO removal remains unclear | Yes 5/8, No 0/8, Unclear 3/8 | 4.d | NR | - |
| Karthik, 2025 [48] | Yes | - | Yes 8/8, No 0/8, Unclear 0/8 | 4.d | None reported | No |
| Liu, 2022[49] | Yes | - | Yes 8/8, No 0/8, Unclear 0/8 | 4.d | This work was supported by the Natural Science Foundation of Hunan Province (2019JJ30035 and 2018JJ2590). | No |
| Massaro, 2022 [50] | Yes | Occurrence of adverse events after HO removal remains unclear | Yes 7/8, No 0/8, Unclear 1/8 | 4.d | None reported | No |
| Matsumoto, 1999 [51] | Yes | Occurrence of adverse events after HO removal remains unclear, Takeaway lesson unclear | Yes 6/8, No 0/8, Unclear 2/8 | 4.d | None reported | No |
| Papadopoulos, 2004 [52] | Yes | Occurrence of adverse events after HO removal remains unclear | Yes 7/8, No 0/8, Unclear 1/8 | 4.d | None reported | No |
| Pham, 1997 [53] | Yes | Occurrence of adverse events after HO removal remains unclear | Yes 7/8, No 0/8, Unclear 1/8 | 4.d | NR | - |
| Rosenberg, 2019 [54] | Yes | Treatment details unclear with lacking details on RT | Yes 7/8, No 0/8, Unclear 1/8 | 4.d | None reported | No |
| Ruiz Hernández, 2000 [55] | Yes | Intervention and results unclear due to lack of details, Occurrence of adverse events after HO removal remains unclear, Takeaway lesson unclear | Yes 4/8, No 0/8, Unclear 4/8 | 4.d | NR | - |
| Shah, 2023 [56] | Yes | Results unclear- unclear if any progression after resection occurred | Yes 7/8, No 0/8, Unclear 1/8 | 4.d | None reported | No |
| Sugita, 2005 [57] | Yes | Occurrence of adverse events after HO removal remains unclear | Yes 7/8, No 0/8, Unclear 1/8 | 4.d | NR | - |
| Takemoto, 2011 [58] | Yes | - | Yes 8/8, No 0/8, Unclear 0/8 | 4.d | None reported | No |
| Thienpont, 2006 [59] | Yes | Occurrence of adverse events after HO removal remains unclear | Yes 7/8, No 0/8, Unclear 1/8 | 4.d | NR | - |
| Valencia, 2007 [60] | Yes | Occurrence of adverse events after HO removal remains unclear, Takeaway lesson unclear | Yes 6/8, No 0/8, Unclear 2/8 | 4.d | NR | - |
| Wróblewski, 2013 [61] | Yes | - | Yes 8/8, No 0/8, Unclear 0/8 | 4.d | NR | - |
| Zhang, 2014 [62] | Yes | - | Yes 8/8, No 0/8, Unclear 0/8 | 4.d | None reported | No |

Randomized controlled trials, cohort studies, case-control studies, case series, and case reports evaluating heterotopic ossification around the knee were assessed using the corresponding JBI critical appraisal tools. For each study, the table presents the overall inclusion decision, methodological limitations identified across the applicable appraisal domains, the number of checklist items rated Yes, No, or Unclear (excluding items rated not applicable), the corresponding JBI level of evidence, reported conflicts of interest or funding, and whether these disclosures raised notable concerns regarding the interpretation of the study.

Abbreviations: AE, adverse event; COI, conflict of interest; HO, heterotopic ossification; JBI, Joanna Briggs Institute; NA, not applicable; NR, not reported; RoB, risk of bias; RT, radiotherapy.

**References**

1. Bhandary, B., et al., *To study the incidence of heterotopic ossification after anterior cruciate ligament reconstruction.* J Clin Diagn Res, 2013. **7**(5): p. 888-91.

2. Kent, W.T., T.J. Shelton, and J. Eastman, *Heterotopic ossification around the knee after tibial nailing and ipsilateral antegrade and retrograde femoral nailing in the treatment of floating knee injuries.* International Orthopaedics, 2018. **42**(6): p. 1379-1385.

3. Van Nest, D.S., et al., *Low-Dose Aspirin Administered for Venous Thromboembolism Prophylaxis Reduces the Incidence of Heterotopic Ossification in Total Joint Arthroplasty.* Journal of Arthroplasty, 2021. **36**(5): p. 1543-1547.

4. Stannard, J.P., et al., *Heterotopic ossification associated with knee dislocation.* Arthroscopy-the Journal of Arthroscopic and Related Surgery, 2002. **18**(8): p. 835-839.

5. Berven, H., et al., *Comparing case-control study for treatment of proximal tibia fractures with a complete metaphyseal component in two centers with different distinct strategies: fixation with Ilizarov frame or locking plates.* Journal of Orthopaedic Surgery and Research, 2018. **13**.

6. Cipriano, C., S.G. Pill, and J. Rosenstock, *Radiation Therapy for Preventing Recurrence of Neurogenic Heterotopic Ossification.* Orthopedics (Online), 2009. **32**(9): p. 685-689.

7. Abdelfettah, Y., et al., *Functional outcomes after surgery for neurogenic heterotopic ossifications: 17 cases collected at the Department of physical medicine and rehabilitation, Casablanca University Hospital.* Annals of Physical and Rehabilitation Medicine, 2012. **55**: p. e173-e174+e176.

8. Barrack, R.L., et al., *Heterotopic ossification after revision total knee arthroplasty.* Clinical Orthopaedics and Related Research, 2002(404): p. 208-213.

9. Belhaj, K., et al., *Functional results of surgery neurogenic heterotopic ossification in patients with severe traumatic brain injury: About 19 cases.* Annals of Physical and Rehabilitation Medicine, 2013. **56**: p. e406.

10. Charnley, G., et al., *Excision of heterotopic ossification around the knee following brain injury.* Injury-International Journal of the Care of the Injured, 1996. **27**(2): p. 125-128.

11. Chidel, M.A., J.H. Suh, and M.B. Matejczyk, *Radiation prophylaxis for heterotopic ossification of the knee.* Journal of Arthroplasty, 2001. **16**(1): p. 1-6.

12. Dalury, D.F. and W.A. Jiranek, *The incidence of heterotopic ossification after total knee arthroplasty.* Journal of Arthroplasty, 2004. **19**(4): p. 447-452.

13. Daugherty, L.C., et al., *Radiation prophylaxis as primary prevention of heterotopic ossification of the knee: Classification of disease and indications for treatment.* Journal of Radiation Oncology, 2013. **2**(1): p. 87-94.

14. Freije, S.L., et al., *A Retrospective Analysis of 287 Patients Undergoing Prophylactic Radiation Therapy for the Prevention of Heterotopic Ossification.* Adv Radiat Oncol, 2021. **6**(3): p. 100625.

15. Fuller, D.A., A. Mark, and M.A. Keenan, *Excision of heterotopic ossification from the knee: a functional outcome study.* Clin Orthop Relat Res, 2005. **438**: p. 197-203.

16. Ippolito, E., et al., *Excision for the treatment of periarticular ossification of the knee in patients who have a traumatic brain injury.* Journal of Bone and Joint Surgery-American Volume, 1999. **81A**(6): p. 783-789.

17. Kerdoncuff, V., et al., *Heterotopic ossification in Guillain-Barré syndrome.* Annales de Readaptation et de Medecine Physique, 2002. **45**(5): p. 198-203.

18. Kolessar, D.J., S.D. Katz, and M.A. Keenan, *Functional outcome following surgical resection of heterotopic ossification in patients with brain injury.* Journal of Head Trauma Rehabilitation, 1996. **11**(4): p. 78-87.

19. Mills, W.J. and N. Tejwani, *Heterotopic ossification after knee dislocation: the predictive value of the injury severity score.* J Orthop Trauma, 2003. **17**(5): p. 338-45.

20. Mishra, M.V., et al., *Safety and efficacy of radiation therapy as secondary prophylaxis for heterotopic ossification of non-hip joints.* J Med Imaging Radiat Oncol, 2011. **55**(3): p. 333-6.

21. Mitsionis, G.I., et al., *Functional outcome after excision of heterotopic ossification about the knee in ICU patients.* International Orthopaedics, 2009. **33**(6): p. 1619-1625.

22. Ogilvie-Harris, D.J. and A. Sekyi-Otu, *Periarticular heterotopic ossification: a complication of arthroscopic anterior cruciate ligament reconstruction using a two-incision technique.* Arthroscopy, 1995. **11**(6): p. 676-9.

23. Parvizi, J., G.P. Duffy, and R.T. Trousdale, *Total knee arthroplasty in patients with ankylosing spondylitis.* Journal of Bone and Joint Surgery-American Volume, 2001. **83A**(9): p. 1312-1316.

24. Rader, C.P., et al., *Heterotopic ossification after total knee arthroplasty. 54/615 cases after 1-6 years' follow-up.* Acta Orthop Scand, 1997. **68**(1): p. 46-50.

25. Subbarao, J.V., B.A. Nemchausky, and M. Gratzer, *Resection of heterotopic ossification and Didronel therapy--regaining wheelchair independence in the spinal cord injured patient.* J Am Paraplegia Soc, 1987. **10**(1): p. 3-7.

26. Alturki, A.A., S.A. Aldeghaither, and A.A. Alhandi, *Severe heterotopic ossification post total knee arthroplasty in a patient with rheumatoid arthritis: a case report.* J Surg Case Rep, 2020. **2020**(3): p. rjz390.

27. Anderson, M.C. and R.L. Lais, *Excision of heterotopic ossification of the popliteal space following traumatic brain injury.* Journal of Orthopaedic Trauma, 2004. **18**(3): p. 190-192.

28. Balen, P.F. and C.A. Helms, *Bony ankylosis following thermal and electrical injury.* Skeletal Radiol, 2001. **30**(7): p. 393-7.

29. Baroudi, M., P. Derome, and M. Malo, *Severe heterotopic ossification and stiffness after revision knee surgery for a periprosthetic fracture.* Arthroplast Today, 2017. **3**(3): p. 147-150.

30. Bellemans, J., et al., *Severe heterotopic ossifications after total knee arthroplasty.* Acta Orthop Belg, 1999. **65**(1): p. 98-101.

31. Bragg, J.T., et al., *Heterotopic Ossification After Anterior Cruciate Ligament Reconstruction With Quadriceps Tendon Autograft: A Case Report.* JBJS Case Connect, 2022. **12**(4).

32. Brown, A. and R.D. Banerjee, *Severe heterotopic ossification following total knee replacement.* Annals of the Royal College of Surgeons of England, 2018. **100**(6): p. E150-E153.

33. Camillieri, G., et al., *Patellar tendon ossification after anterior cruciate ligament reconstruction using bone - patellar tendon - bone autograft.* Bmc Musculoskeletal Disorders, 2013. **14**.

34. Cho, S.H., et al., *A case of extensive heterotopic ossification following multiple ligament reconstruction after severe knee trauma.* European Journal of Orthopaedic Surgery and Traumatology, 2011. **21**(6): p. 435-437.

35. Choi, J.H., et al., *Successful Total Knee Arthroplasty in a Patient With Contralateral Ankylosis Due to Severe Heterotopic Ossification.* Cureus, 2022. **14**(5): p. e24941.

36. Daniilidis, K., B. Vogt, and M.J. Raschke, *Symptomatic heterotopic ossification: seven years after patella fracture.* Musculoskelet Surg, 2013. **97**(2): p. 169-71.

37. Davis, C., G.P. Kolovich, and T.J. Scharschmidt, *Atraumatic heterotopic ossification in the setting of prolonged intubation because of H1N1 influenza: a case report.* Orthop Surg, 2012. **4**(4): p. 258-62.

38. Erdil, M., et al., *Heterotopic bone formation following anterior cruciate ligament reconstruction with BPTB autograft.* Acta Orthopaedica Et Traumatologica Turcica, 2012. **46**(1): p. 72-76.

39. Espandar, R. and B. Haghpanah, *Acceptable outcome following resection of bilateral large popliteal space heterotopic ossification masses in a spinal cord injured patient: a case report.* Journal of Orthopaedic Surgery and Research, 2010. **5**.

40. Estel, K., et al., *Fulminant Heterotopic Ossification of the Lower Extremity After Gunshot Injury and Blunt Trauma: A Case Report.* Military Medicine, 2024. **189**(7-8): p. e1826-e1831.

41. Firoozabadi, A.M., et al., *Functional outcomes may vary over time after patellar tendon and knee intra-articular heterotopic ossification excision: A case report.* Int J Surg Case Rep, 2025. **127**: p. 110773.

42. Gibson, C.J. and K.R. Poduri, *Heterotopic ossification as a complication of toxic epidermal necrolysis.* Archives of Physical Medicine and Rehabilitation, 1997. **78**(7): p. 774-776.

43. Hari Krishnan, B., Y. Sharma, and A. Prabhakara, *A rare case of intra-articular heterotopic ossification of knee following intra-medullary nailing of fracture tibia in a patient with fat embolism.* Med J Armed Forces India, 2016. **72**(Suppl 1): p. S115-s119.

44. Hoffer, A.J., et al., *Excision of Intra-articular Knee Heterotopic Ossification Using a 70° Arthroscope.* Case Reports in Orthopedics, 2024. **2024**.

45. Iida, K., et al., *Surgical excision of heterotopic ossification associated with anti-N-methyl-d-aspartate receptor encephalitis: A case report.* Int J Surg Case Rep, 2021. **89**: p. 106643.

46. Ivey, M., *Myositis ossificans of the thigh following manipulation of the knee. A case report.* Clin Orthop Relat Res, 1985(198): p. 102-5.

47. Jacobs, J.W.G., et al., *Polyarticular heterotopic ossification complicating critical illness.* Rheumatology, 1999. **38**(11): p. 1145-1149.

48. Karthik, M.S., A. Mohammed, and A. Parthasarathy, *Rare Case of Heterotopic Ossification Impinging on the Quadriceps Mechanism Following Total Knee Replacement: A Case Report.* J Orthop Case Rep, 2025. **15**(9): p. 83-87.

49. Liu, Q., et al., *Patellar Tendon Reconstruction Using Autologous Hamstring Tendons for the Treatment of Extensive Patellar Tendon Ossification.* Orthop Surg, 2022. **14**(11): p. 3119-3124.

50. Massaro, M., et al., *Severe Quadriceps Heterotopic Ossification after Knee Revision Arthroplasty in a 42-Year-Old Suffering from Rheumatoid Arthritis: A Case Report.* Osteology, 2022. **2**(4): p. 161-165.

51. Matsumoto, H., et al., *Extensive post-traumatic ossification of the patellar tendon. A report of two cases.* J Bone Joint Surg Br, 1999. **81**(1): p. 34-6.

52. Papadopoulos, A.X., et al., *Operative treatment of unilateral bicondylar Hoffa fractures.* Journal of Orthopaedic Trauma, 2004. **18**(2): p. 119-122.

53. Pham, J. and R. Kumar, *Heterotopic ossification after total knee arthroplasty.* Am J Orthop (Belle Mead NJ), 1997. **26**(2): p. 141-3.

54. Rosenberg, D.M., et al., *Radiation-Induced Sarcoma After Heterotopic Ossification Prophylaxis: A Case Report.* JBJS Case Connect, 2019. **9**(4): p. e0146.

55. Ruiz Hernández, G., et al., *[Periarticular heterotopic ossification secondary to central neurogenic dysfunction].* Rev Esp Med Nucl, 2000. **19**(7): p. 495-9.

56. Shah, S.P., et al., *Radiation therapy in non-traumatic myositis ossificans of popliteal region: a case report.* Journal of Radiotherapy in Practice, 2023. **22**.

57. Sugita, A., et al., *Heterotopic ossification in bilateral knee and hip joints after long-term sedation.* Journal of Bone and Mineral Metabolism, 2005. **23**(4): p. 329-332.

58. Takemoto, R.C., D. Epstein, and T.M. McLaurin, *Intra- and Periarticular Heterotopic Ossification in the Knee After a Low-Velocity Gunshot Wound Treated With Retrograde Intramedullary Nailing of the Femur.* Journal of Orthopaedic Trauma, 2011. **25**(7): p. E77-E82.

59. Thienpont, E., T. Schmalzried, and J. Bellemans, *Ankylosis due to heterotopic ossification following primary total knee arthroplasty.* Acta Orthop Belg, 2006. **72**(4): p. 502-6.

60. Valencia, H. and C. Gavín, *Infrapatellar heterotopic ossification after anterior cruciate ligament reconstruction.* Knee Surgery Sports Traumatology Arthroscopy, 2007. **15**(1): p. 39-42.

61. Wróblewski, R., I. Pokrzywnicka-Gajek, and J. Kowalczewski, *Heterotopic ossifications after two-stage septic revision knee arthroplasty in a rheumatoid arthritis patient.* Reumatologia, 2013. **51**(5): p. 389-393.

62. Zhang, X., et al., *Acquired heterotopic ossification in hips and knees following encephalitis: case report and literature review.* BMC Surg, 2014. **14**: p. 74.
