# Supplementary material for: The prevention of heterotopic ossification around the knee: a scoping review
Source: BMC Musculoskelet Disord. 2026 Aug 1;27:651. doi: 10.1186/s12891-026-10318-w (PMC13428452; doi:10.1186/s12891-026-10318-w)
Supplement: Supplementary file 18 — Supplementary Material 18. [file 12891_2026_10318_MOESM18_ESM.docx]

**Supplement S18:** Modality-specific summary of heterotopic ossification requiring further intervention.

| **Prophylactic modality** | **Studies reporting the outcome, n/N (%)** | **Studies contributing population-specific data, n/N (%)** | **Knees with available population-specific data, n** | **Knees requiring further intervention due to HO, n/N (%)** |
| --- | --- | --- | --- | --- |
| Continuous passive motion | 6/8 (75.0%) | 6/8 (75.0%) | 544 | 16/544 (2.9%) |
| Pharmacological prophylaxis | 18/21 (85.7%) | 18/21 (85.7%) | 50 | 2/50 (4.0%) |
| Radiotherapy | 13/18 (72.2%) | 12/18 (66.7%)* | 38 | 0/38 (0.0%) |
| Surgical techniques | 0/3 (0.0%)† | 0/3 (0.0%) | NR | NR |
| Combination therapy | 13/14 (92.9%) | 13/14 (92.9%) | 18 | 0/18 (0.0%) |

Values represent crude reported event proportions based only on studies with extractable, population-specific numerators and denominators. Studies reporting only overall-cohort data were counted as reporting the outcome but did not contribute to the quantitative summary. Reported event proportions should not be compared directly across modalities because of substantial clinical and methodological heterogeneity.

* Cipriano et al. reported further interventions for the overall study groups, but knee-specific data were unavailable; the study was therefore not included in the quantitative denominator.

† Berven et al. reported overall reoperation rates after external and internal fixation, but did not attribute these reoperations specifically to HO. These data were therefore not classified as further interventions due to HO. Bhandary et al. and Kent et al. did not report this outcome.
