# Supplementary material for: The prevention of heterotopic ossification around the knee: a scoping review
Source: BMC Musculoskelet Disord. 2026 Aug 1;27:651. doi: 10.1186/s12891-026-10318-w (PMC13428452; doi:10.1186/s12891-026-10318-w)
Supplement: Supplementary file 19 — Supplementary Material 19. [file 12891_2026_10318_MOESM19_ESM.docx]

**Supplement S19:** Sensitivity analysis restricted to full-text reports.

| **Modality** | **Original evidence base** | **Full-text-only evidence base** | **Any HO: original / sensitivity** | **Clinically relevant HO: original / sensitivity** | **Further intervention: original / sensitivity** |
| --- | --- | --- | --- | --- | --- |
| CPM | 8 studies, 557 knees | 6 studies, 546 knees | 93/544 (17.1%) / 93/544 (17.1%) | 15/544 (2.8%) / 15/544 (2.8%) | 16/544 (2.9%) / 16/544 (2.9%) |
| Pharmacological prophylaxis | 21 studies, 822 knees | Unchanged | 104/796 (13.1%) / unchanged | 3/50 (6.0%) / unchanged | 2/50 (4.0%) / unchanged |
| Radiotherapy | 18 studies, 47 analyzed knees | Unchanged | 2/24 (8.3%) / unchanged | 0/38 (0.0%) / unchanged | 0/38 (0.0%) / unchanged |
| Surgical techniques | 3 studies, 199 analyzed knees in the intervention groups | Unchanged | Not aggregated because of substantial heterogeneity | Not aggregated | Not aggregated |
| Combination therapy | 14 studies, 20 knees | Unchanged | 0/11 (0.0%) / unchanged | 0/15 (0.0%) / unchanged | 0/18 (0.0%) / unchanged |

Values represent crude reported event proportions based only on studies with extractable, population-specific numerators and denominators. Studies reporting only overall-cohort data were counted as reporting the outcome but did not contribute to the quantitative summary. The sensitivity analysis excluded abstract-only reports. Reported event proportions should not be compared directly across modalities because of substantial clinical and methodological heterogeneity.
